# Supplementary material for: scapGNN: A graph neural network–based framework for active pathway and gene module inference from single-cell multi-omics data
Source: PLoS Biol. 2023 Nov 13;21(11):e3002369. doi: 10.1371/journal.pbio.3002369 (PMC10681325; doi:10.1371/journal.pbio.3002369)
Supplement: S1 Text — (DOCX) [file pbio.3002369.s001.docx]

**S1 Text. Supplementary Methods**

*Evaluating the running time*

We selected GSE75748 (cell lines), the scRNA-seq dataset with the highest number of genes detected, as the test data (S2 Table). Different numbers of cell samples (including 100, 300, 500, 700, and 1000 cells) were randomly selected to construct different scales of data sets. KEGG pathway data (including 186 biological pathways) from the C2 gene set of the MSigDB database were used. The runtime for pathway activity score calculation of AUCell, Pagoda2, UniPath, and scapGNN and the GNN module of scapGNN were counted on the same device (Windows 10 system, 4 cores, 16 G RAM). The default parameters of each method are used.

*Data sets*

For the evaluation of cell clustering performance, 16 scRNA-seq datasets from different platforms (including CEL-Seq2, 10x Genomics, SMARTer, Fluidigm C1, inDrop, and Smart-seq2), species (human and mouse), and tissues (including blood, lung, choroids, brain, breast, stem cell, midbrain, liver, pancreas, and hypothalamus) constitute the cell clustering benchmark datasets (S2 Table). The Cell type dataset [1] determines the transcriptomes of pancreatic cells from two strains of mice. Cell types include rare ghrelin-expressing epsilon-cells, exocrine cell types, vascular cells, Schwann cells, quiescent and activated pancreatic stellate cells, and immune cells. Cell subtype dataset detects the transcriptomes of the human embryonic stem cell (ESC)-derived dopaminergic neurons by 10x Genomics single-cell sequencing [2]. Time series dataset profiled temporal scRNA-seq profiling along DEC (definitive endoderm cell) differentiation [2]. The adult mouse brain dataset that was used for the single-cell multi-omics data integration evaluation profiled 10309 nuclei from adult mouse brains using SNARE-seq, a droplet-based method to simultaneously profile gene expression and chromatin accessibility in each of thousands of single nuclei [3].

K562 dataset, A549 dataset, T cell dataset, and B cell dataset are homogeneous datasets containing only one cell type. GM12878 dataset, ESC dataset, and T cell and B cell dataset are heterogeneous datasets that contain multiple cell types. These data were used to systematically assess the accuracy of pathway activity identification.

For the mouse spermatogenesis dataset, Chen Y et al. develop an approach to purify all types of homogeneous spermatogenic cells by combining transgenic labeling and synchronization of the cycle of the seminiferous epithelium, and subsequent single-cell RNA-sequencing [4]. Mouse early embryo development dataset profiles RNA transcription of mouse blastomeres from seven-time points during mouse preimplantation development via scNOME-seq [5]. Mouse cortical brain dataset and PBMC dataset were used to analyze the performance of scapGNN on scATAC-seq data.

*Calculating the importance of genes in the pathway*

We use the genes in a pathway as restart nodes in the gene-cell association network. When the diffusion of the RWR algorithm reaches a steady state, the stationary probability values of these seed genes were used as the relative importance between them in the pathway. We finally normalized the stationary probability values to be between 0 and 1 (divided by the maximum value). At the same time, we constructed gene association networks for each pathway using the normalized stationary probability values of genes as node weights and their connections in gene-cell association networks as edges.

*Ablation experiment*

We constructed two protocols of ablation experiments to test the uniformity of scapGNN. First, we constructed DNNAE that discards the graph autoencoder and integrates the results of the cell correlation network, the gene correlation network, and the result of the deep neural network autoencoder into a gene-cell network. Secondly, we ignore the deep neural network autoencoder and use the signals of LTMG to fill in the link between genes and cells (GAE+LTMG). The product of the TRSs matrix of LTMG and the corresponding gene-cell expression matrix is used as the weight of the gene-cell edge. It is subsequently integrated with the cell-cell association network and the gene-gene association network obtained by the graph autoencoder into a gene-cell network. Third, we remove the LTMG regularisation using only the DNNAE and GAE results (scapGNN-LTMG). For each gene-cell network, the pathway activity score is calculated by the RWR algorithm.

*Evaluation of the pseudotime inference performance for scapGNN*

For the single-cell pathway activity score matrix, Monocle 3 (v1.2.7) with default parameters was used to cluster cells and construct single-cell trajectories. For the Time series dataset, we selected three points in the 0 hour cell population as the root nodes of the single-cell trajectories for scapGNN, AUCell, Pagoda2, and UniPath and calculated the pseudotime of the cells using the order_cells function of Monocle 3 (S4A Fig). The mminjk.pw function of the R package mpmi is used to calculate the mixed-pair Bias Corrected Mutual Information (BCMI) between pseudotime inferred based on pathway activity scores (continuous variables) and true cellular timestamps (discrete variables) [6]. Let *X* be the true cellular timestamps, the probability $P\left( X=x_{i} \right)=p_{i}, i\in\{1,\ldots,g\}$, *g* denotes the number of groups in *X*. Let Y be the inferred pseudotime with density function $f(y)$. For each $x_{i}$ , we have a continuous density function $f_{Y|X=x_{i}}=x_{i}\left( y \right),$ is simply denoted as $f_{i}(y)$. The mutual information is calculated as follows:

$I\left( X, Y \right)=\sum_{i=1}^{g} p_{i}\int_{y\mathfrak{\in R}}f_{i}(y)log(\frac{f_{i}(y)}{f(y)})dy$ (S1)

The jackknife, which is a nonparametric statistical procedure that can be thought of as a computational simplification of the bootstrap, was used to gives a correction for estimation bias. A high BCMI value indicates the pseudotime is more similar to the true time series.

For single-cell multi-omics data integration in time-series analyses using mouse skin dataset, the pseudotime provided by the Jones study, the scapGNN, and scDART inferred were continuous. Therefore, the Pearson correlation was used to assess the similarity.

*Functionally modular evaluation for cell-phenotype-associated gene modules*

We have performed gene ontology (GO) enrichment for gene modules of activated stellate cells in the cell type dataset, eProg1b cells in the cell subtype dataset, and 36 h cells in the time series dataset respectively. If two genes have a common GO term, they will be labeled as functionally linked genes. We use the proportion of functionally linked genes in this gene module as an indicator. As a control, we randomly selected genes that were equal to the number of genes contained in the gene module from the corresponding single-cell dataset and counted the proportion of functionally linked genes. The random sampling process was repeated 10 times and the mean value was taken.

**References**

1. Baron M, Veres A, Wolock SL, Faust AL, Gaujoux R, Vetere A, et al. A Single-Cell Transcriptomic Map of the Human and Mouse Pancreas Reveals Inter- and Intra-cell Population Structure. Cell systems. 2016;3(4):346-60.e4. Epub 2016/10/28. doi: 10.1016/j.cels.2016.08.011. PubMed PMID: 27667365; PubMed Central PMCID: PMCPMC5228327.

2. La Manno G, Gyllborg D, Codeluppi S, Nishimura K, Salto C, Zeisel A, et al. Molecular Diversity of Midbrain Development in Mouse, Human, and Stem Cells. Cell. 2016;167(2):566-80.e19. Epub 2016/10/08. doi: 10.1016/j.cell.2016.09.027. PubMed PMID: 27716510; PubMed Central PMCID: PMCPMC5055122.

3. Chen S, Lake BB, Zhang K. High-throughput sequencing of the transcriptome and chromatin accessibility in the same cell. Nat Biotechnol. 2019;37(12):1452-7. Epub 2019/10/16. doi: 10.1038/s41587-019-0290-0. PubMed PMID: 31611697; PubMed Central PMCID: PMCPMC6893138.

4. Chen Y, Zheng Y, Gao Y, Lin Z, Yang S, Wang T, et al. Single-cell RNA-seq uncovers dynamic processes and critical regulators in mouse spermatogenesis. Cell Research. 2018;28(9):879-96. doi: 10.1038/s41422-018-0074-y.

5. Wang Y, Yuan P, Yan Z, Yang M, Huo Y, Nie Y, et al. Single-cell multiomics sequencing reveals the functional regulatory landscape of early embryos. Nat Commun. 2021;12(1):1247. Epub 2021/02/25. doi: 10.1038/s41467-021-21409-8. PubMed PMID: 33623021; PubMed Central PMCID: PMCPMC7902657.

6. Pardy C, Galbraith S, Wilson SR. Integrative exploration of large high-dimensional datasets. The Annals of Applied Statistics. 2018;12:178-99.
