# Supplementary material for: scapGNN: A graph neural network–based framework for active pathway and gene module inference from single-cell multi-omics data
Source: PLoS Biol. 2023 Nov 13;21(11):e3002369. doi: 10.1371/journal.pbio.3002369 (PMC10681325; doi:10.1371/journal.pbio.3002369)
Supplement: S1 Fig — First, the GNN model of scapGNN constructs a gene–cell association network for gene expression profiles of scRNA-seq data and gene activity matrix of scATAC-seq data, respectively. Second, Brown’s method integrates 2 gene–cell association networks into a combined gene–cell association network. Finally, the RWR algorithm is used to calculate pathway activity scores and identify cell phenotype–associated gene modules with multi-omics information. GNN, graph neural network; RWR, random walk with restart; scATAC-seq, single-cell ATAC sequencing; scRNA-seq, single-cell RNA sequencing. (PDF) [file pbio.3002369.s002.pdf]

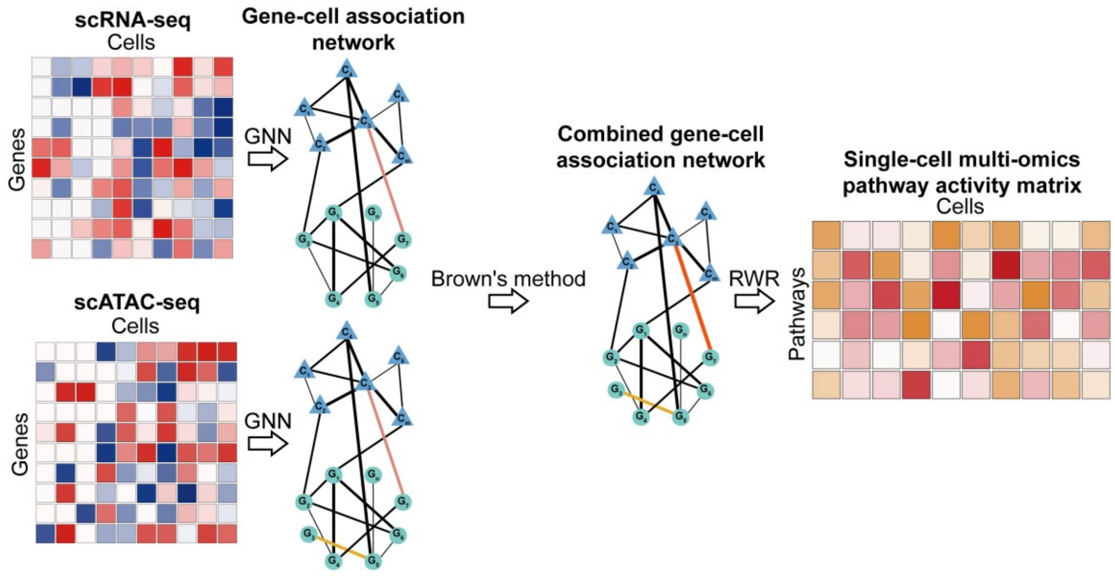

S1 Fig. The workflow of integrating single-cell multi-omics data by scapGNN. First, the GNN model of scapGNN constructs a gene–cell association network for gene expression profiles of scRNA-seq data and gene activity matrix of scATAC-seq data, respectively. Second, Brown's method integrates two gene–cell association networks into a combined gene–cell association network. Finally, the RWR algorithm is used to calculate pathway activity scores and identify cell phenotype–associated gene modules with multi-omics information.
