# Supplementary material for: scapGNN: A graph neural network–based framework for active pathway and gene module inference from single-cell multi-omics data
Source: PLoS Biol. 2023 Nov 13;21(11):e3002369. doi: 10.1371/journal.pbio.3002369 (PMC10681325; doi:10.1371/journal.pbio.3002369)
Supplement: S2 Fig — tSNE visualizations of cell type data (A), cell subtype data (B), and time series data (C) based on pathway activity scores using the 4 pathway enrichment methods (AUCell, Pagoda2, UniPath, and scapGNN). The data underlying this figure can be found in S1 Data. (PDF) [file pbio.3002369.s003.pdf]

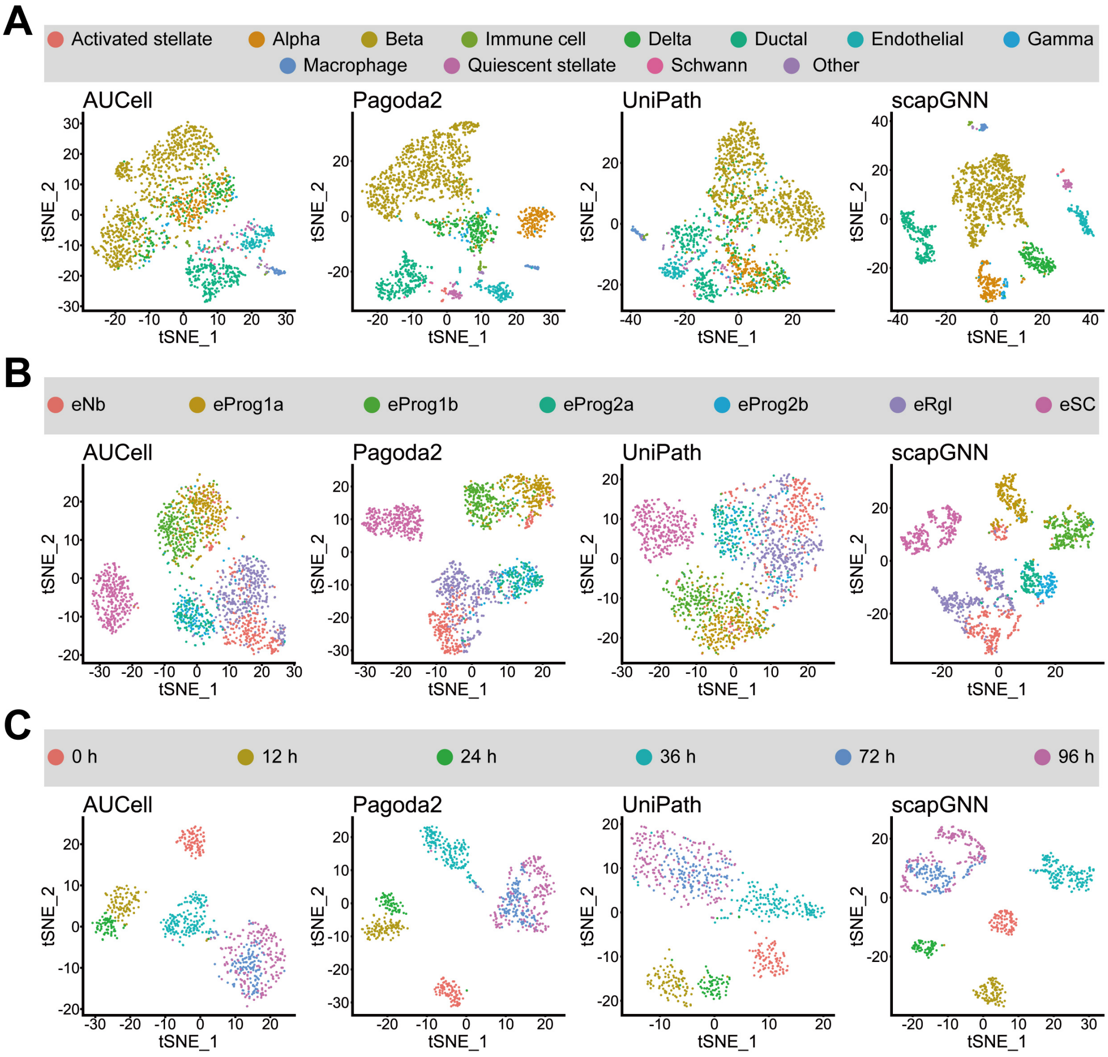

S2 Fig. Nonlinear dimensional reduction visualization of pathway activity scores and difference analysis of the pathway activity. tSNE visualizations of cell type data (**A**), cell subtype data (**B**), and time series data (**C**) based on pathway activity scores using the four pathway enrichment methods (AUCell, Pagoda2, UniPath, and scapGNN). The data underlying this figure can be found in S1 Data.
