# Supplementary material for: scapGNN: A graph neural network–based framework for active pathway and gene module inference from single-cell multi-omics data
Source: PLoS Biol. 2023 Nov 13;21(11):e3002369. doi: 10.1371/journal.pbio.3002369 (PMC10681325; doi:10.1371/journal.pbio.3002369)
Supplement: S3 Fig — The data underlying this figure can be found in S7 Data. ARI, adjusted rand index; NMI, normalized mutual information; scRNA-seq, single-cell RNA sequencing; SW, silhouette width. (PDF) [file pbio.3002369.s004.pdf]

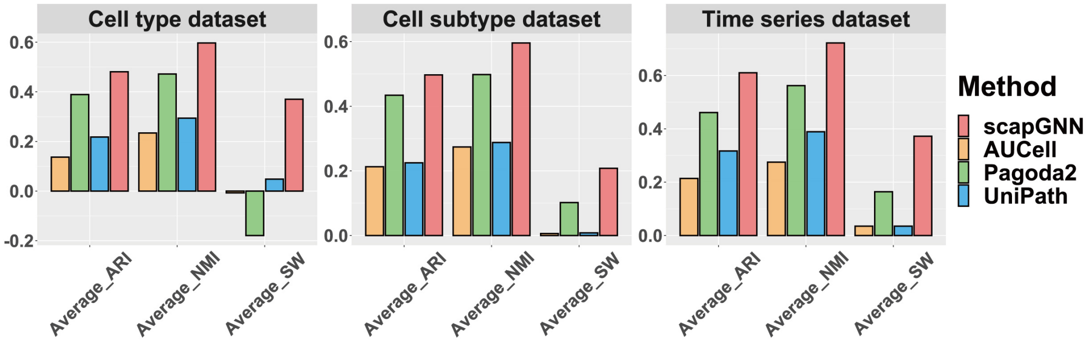

S3 Fig. Bar graphs of three cell clustering accuracy indicators (ARI, NMI, and SW) that evaluated the cell clustering results of AUCell, Pagoda2, UniPath, and scapGNN in the three scRNA-seq datasets using the 10 cell clustering methods. The data underlying this figure can be found in S7 Data.
