# Supplementary material for: scapGNN: A graph neural network–based framework for active pathway and gene module inference from single-cell multi-omics data
Source: PLoS Biol. 2023 Nov 13;21(11):e3002369. doi: 10.1371/journal.pbio.3002369 (PMC10681325; doi:10.1371/journal.pbio.3002369)
Supplement: S4 Fig — Three starting points from the 0-h cell population in the time series dataset were selected to infer the pseudotime for AUCell (A), Pagoda2 (B), UniPath (C), and scapGNN (D). (E) Bar graphs of BCMI between pseudotimes inferred based on pathway activity scores and true cellular timestamps of the time series dataset. The data underlying this figure can be found in S7 Data. (PDF) [file pbio.3002369.s005.pdf]

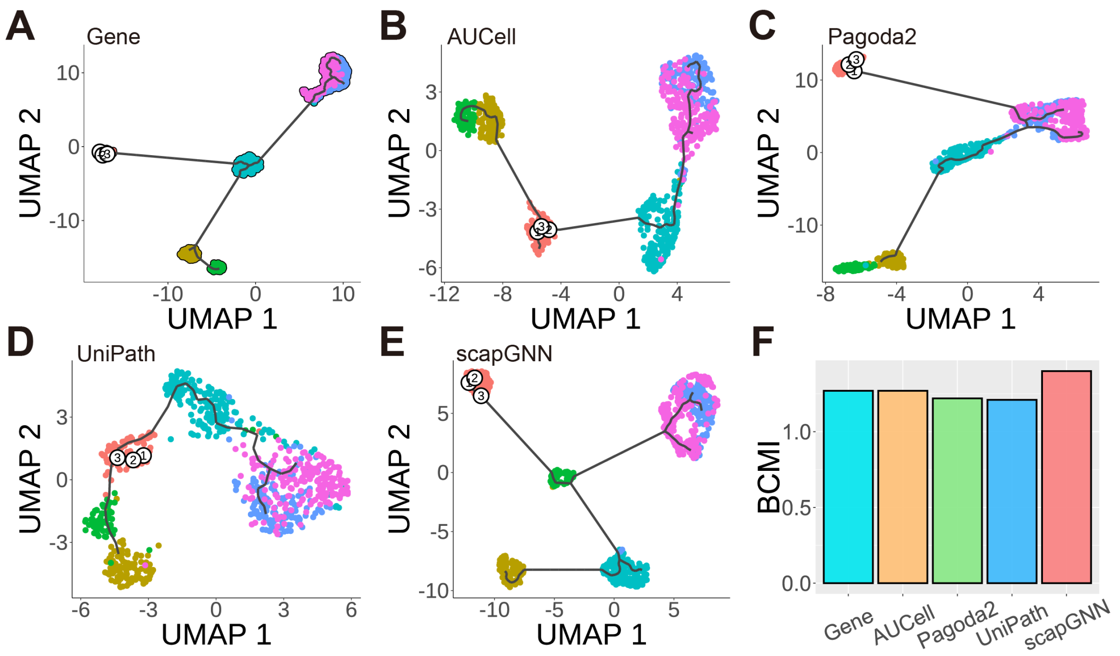

S4 Fig. Three starting points from the 0-h cell population in the time series dataset were selected to infer the pseudotime for AUCell (**A**), Pagoda2 (**B**), UniPath (**C**), and scapGNN (**D**). (**E**) Bar graphs of BCMI between pseudotimes inferred based on pathway activity scores and true cellular timestamps of the time series dataset. The data underlying this figure can be found in S7 Data.
