# Supplementary material for: scapGNN: A graph neural network–based framework for active pathway and gene module inference from single-cell multi-omics data
Source: PLoS Biol. 2023 Nov 13;21(11):e3002369. doi: 10.1371/journal.pbio.3002369 (PMC10681325; doi:10.1371/journal.pbio.3002369)
Supplement: S5 Fig — (PDF) [file pbio.3002369.s006.pdf]

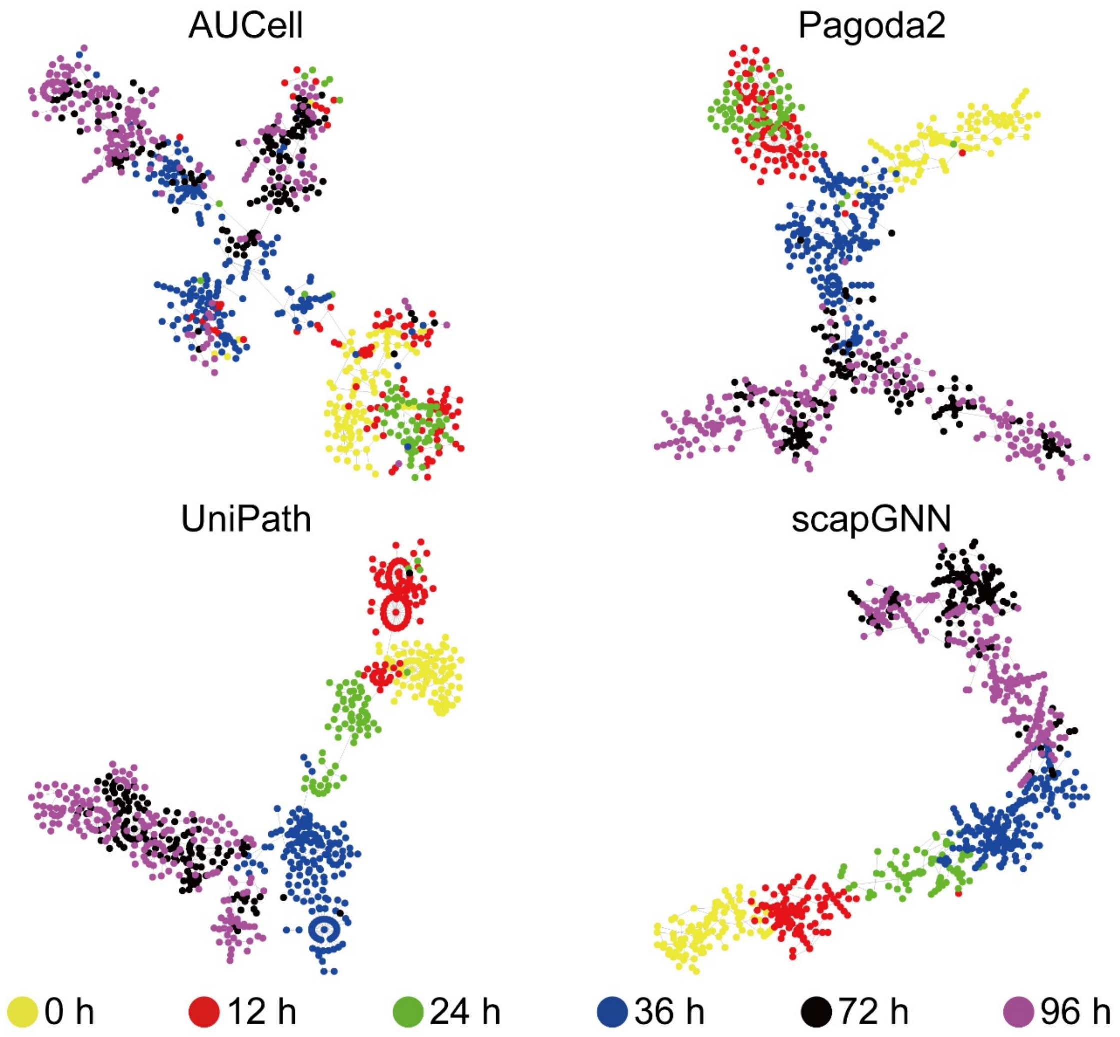

S5 Fig. Temporal-ordering analysis of AUCell, Pagoda2, UniPath, and scapGNN for the time series dataset following the strategy of UniPath.
