# Supplementary material for: scapGNN: A graph neural network–based framework for active pathway and gene module inference from single-cell multi-omics data
Source: PLoS Biol. 2023 Nov 13;21(11):e3002369. doi: 10.1371/journal.pbio.3002369 (PMC10681325; doi:10.1371/journal.pbio.3002369)
Supplement: S6 Fig — (A) A cell community network merged cell nodes of the same cell type. (B) Cell communities of the cell–cell association network were identified, and cell nodes of the same cell community were merged. (PDF) [file pbio.3002369.s007.pdf]

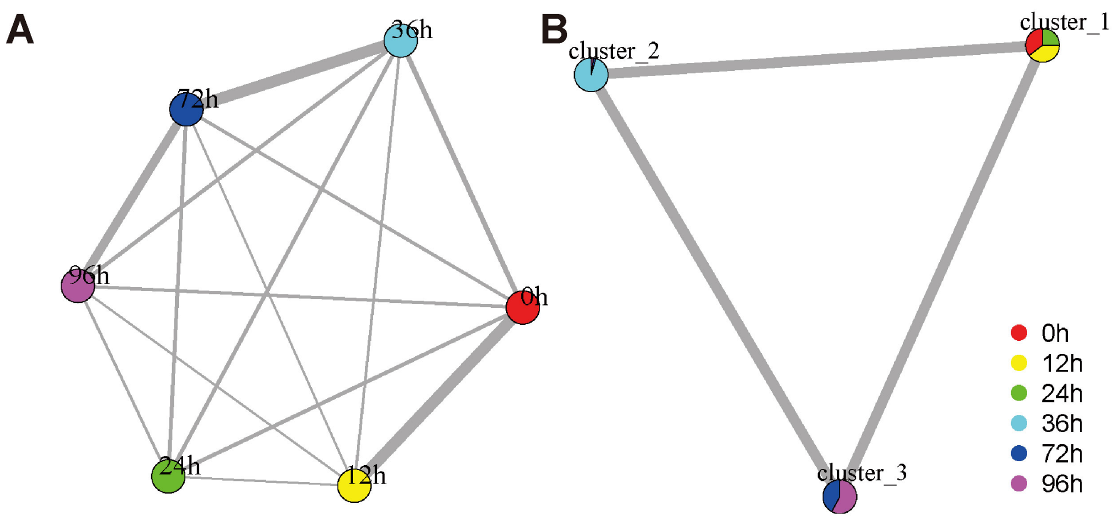

S6 Fig. Cell community network of the time series dataset. (**A**) A cell community network merged cell nodes of the same cell type. (**B**) Cell communities of the cell–cell association network were identified, and cell nodes of the same cell community were merged.
