# Supplementary material for: scapGNN: A graph neural network–based framework for active pathway and gene module inference from single-cell multi-omics data
Source: PLoS Biol. 2023 Nov 13;21(11):e3002369. doi: 10.1371/journal.pbio.3002369 (PMC10681325; doi:10.1371/journal.pbio.3002369)
Supplement: S7 Fig — Box plot of average ARI, average NMI, and average SW of the pathway level from ssGSEA (A) and GSVA (B) using 10 state-of-the-art single-cell clustering methods on 16 scRNA-seq data sets. The data underlying this figure can be found in S1 Data. ARI, adjusted rand index; GSVA, gene set variation analysis; NMI, normalized mutual information; scRNA-seq, single-cell RNA sequencing; ssGSEA, single-sample gene set enrichment analysis; SW, silhouette width. (PDF) [file pbio.3002369.s008.pdf]

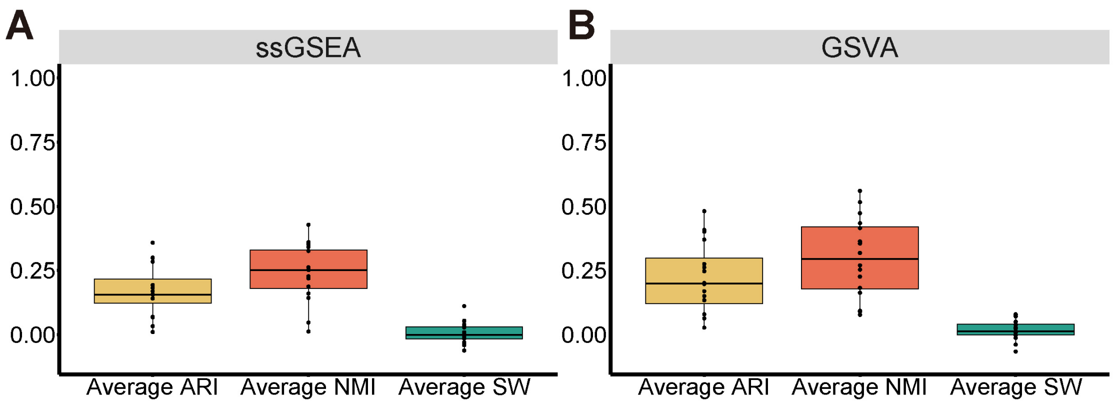

S7 Fig. Application of traditional bulky RNA-seq pathway enrichment analysis methods to scRNA-seq data. Box plot of average ARI, average NMI, and average SW of the pathway level from ssGSEA (**A**) and GSVA (**B**) using 10 state-of-the-art single-cell clustering methods on 16 scRNA-seq data sets. The data underlying this figure can be found in S1 Data.
