# Supplementary material for: scapGNN: A graph neural network–based framework for active pathway and gene module inference from single-cell multi-omics data
Source: PLoS Biol. 2023 Nov 13;21(11):e3002369. doi: 10.1371/journal.pbio.3002369 (PMC10681325; doi:10.1371/journal.pbio.3002369)
Supplement: S8 Fig — (A) Box plots of the average number of genes detected in cells for the high–gene number group datasets and the low–gene number group datasets. (B) Box plots of cell clustering accuracy indicators (ARI, NMI, and SW) for the 4 single-cell pathway activity scoring methods in the 2-group benchmark datasets. The data underlying this figure can be found in S7 Data. ARI, adjusted rand index; NMI, normalized mutual information; SW, silhouette width. (PDF) [file pbio.3002369.s009.pdf]

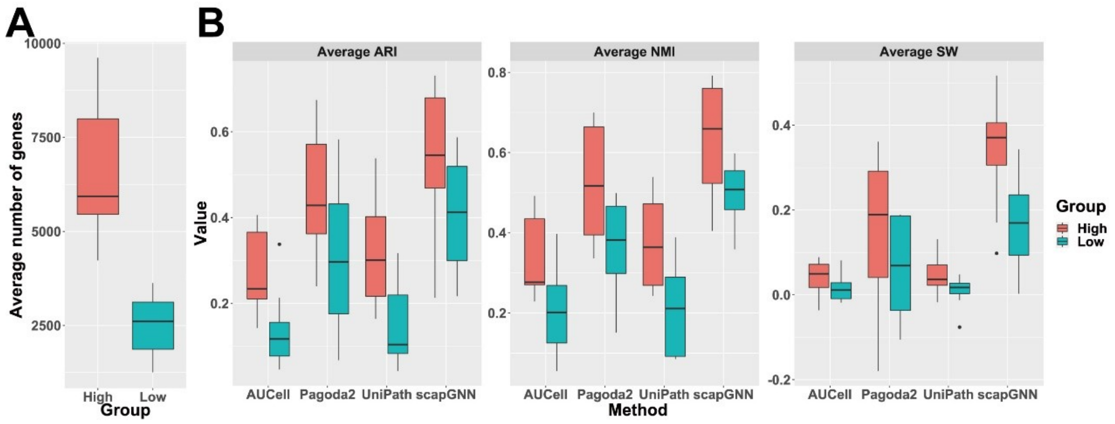

S8 Fig. Performance of cell clustering was assessed according to detected gene numbers. (**A**) Box plots of the average number of genes detected in cells for the high– gene number group datasets and the low–gene number group datasets. (**B**) Box plots of cell clustering accuracy indicators (ARI, NMI, and SW) for the four single-cell pathway activity scoring methods in the two-group benchmark datasets. The data underlying this figure can be found in S7 Data.
