# Supplementary material for: scapGNN: A graph neural network–based framework for active pathway and gene module inference from single-cell multi-omics data
Source: PLoS Biol. 2023 Nov 13;21(11):e3002369. doi: 10.1371/journal.pbio.3002369 (PMC10681325; doi:10.1371/journal.pbio.3002369)
Supplement: S9 Fig — The data underlying this figure can be found in S7 Data. (PDF) [file pbio.3002369.s010.pdf]

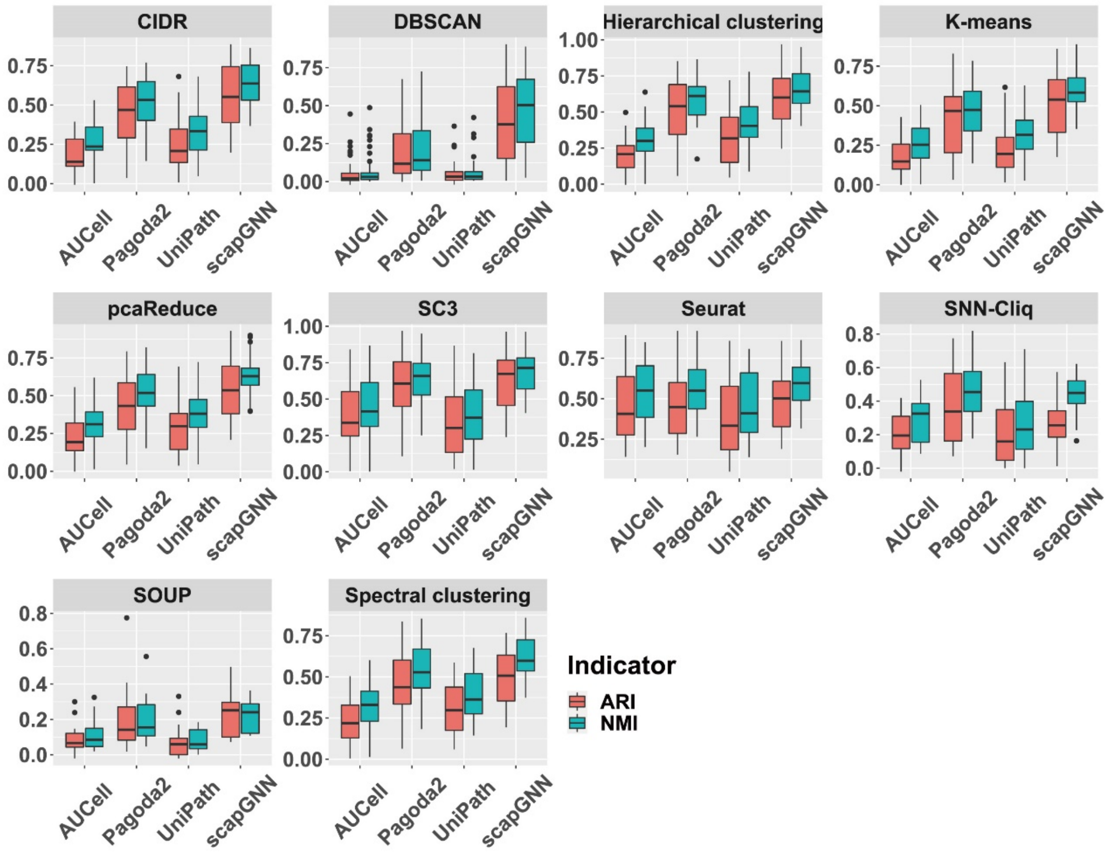

S9 Fig. Box plot of cell clustering accuracy indicators (ARI and NMI) for AUCell, Pagoda2, UniPath, and scapGNN using the 10 cell clustering methods. The data underlying this figure can be found in S7 Data.
