# Supplementary material for: scapGNN: A graph neural network–based framework for active pathway and gene module inference from single-cell multi-omics data
Source: PLoS Biol. 2023 Nov 13;21(11):e3002369. doi: 10.1371/journal.pbio.3002369 (PMC10681325; doi:10.1371/journal.pbio.3002369)
Supplement: S10 Fig — (A) UMAP visualizations included raw gene expression data, integrated gene expression data, and the pathway activity score matrix after data integration using Seurat v4. The graphs show the UMAP plots separated by cell type. (B) Bar plots of 3 cell clustering accuracy indicators (ARI, NMI, and SW) for using scapGNN, AUCell, Pagoda2, and UniPath on the integrated gene expression data. (C) Proportion of A549 cells that detected the corresponding correct cell type marker gene set in the top 1 to 5 of the pathway scores. The data underlying this figure can be found in S7 Data. ARI, adjusted rand index; NMI, normalized mutual information; scRNA-seq, single-cell RNA sequencing; SW, silhouette width; UMAP, Uniform Manifold Approximation and Projection. (PDF) [file pbio.3002369.s011.pdf]

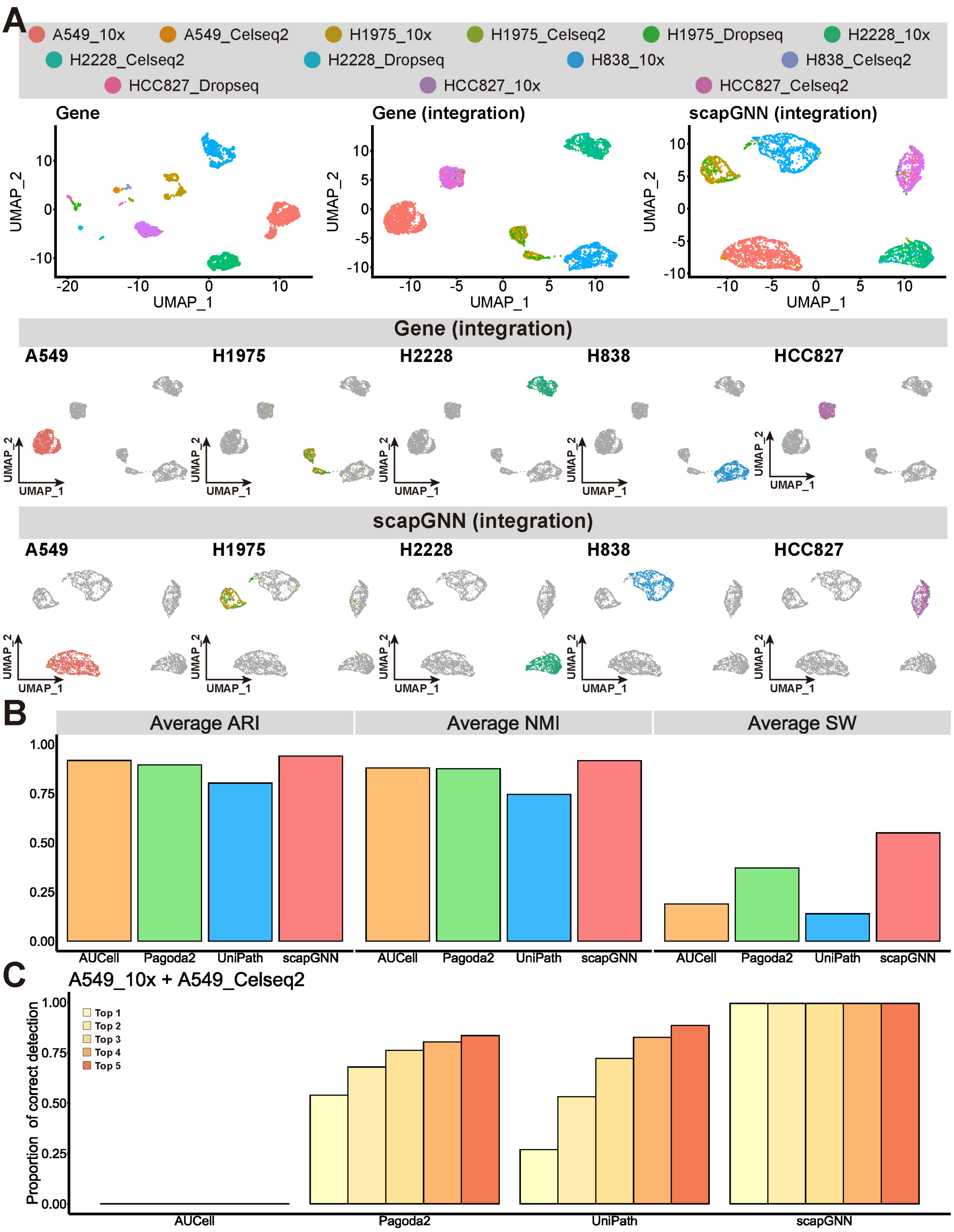

S10 Fig. Performance evaluation of scapGNN on scRNA-seq data with batch effects. (**A**) UMAP visualizations included raw gene expression data, integrated gene expression data, and the pathway activity score matrix after data integration using Seurat v4. The graphs show the UMAP plots separated by cell type. (**B**) Bar plots of three cell clustering accuracy indicators (ARI, NMI, and SW) for using scapGNN, AUCell, Pagoda2, and UniPath on the integrated gene expression data. (**C**) Proportion of A549 cells that detected the corresponding correct cell type marker gene set in the top one to five of the pathway scores. The data underlying this figure can be found in S7 Data.
