# Supplementary material for: scapGNN: A graph neural network–based framework for active pathway and gene module inference from single-cell multi-omics data
Source: PLoS Biol. 2023 Nov 13;21(11):e3002369. doi: 10.1371/journal.pbio.3002369 (PMC10681325; doi:10.1371/journal.pbio.3002369)
Supplement: S11 Fig — (A) Cell clustering tSNE of AUCell, Pagoda2, UniPath, and scapGNN on the mouse cell atlas dataset. (B) Bar graph of cell clustering accuracy indicators (ARI, NMI, and purity). The data underlying this figure can be found in S7 Data. ARI, adjusted rand index; NMI, normalized mutual information; tSNE, t-distributed stochastic neighbor embedding. (PDF) [file pbio.3002369.s012.pdf]

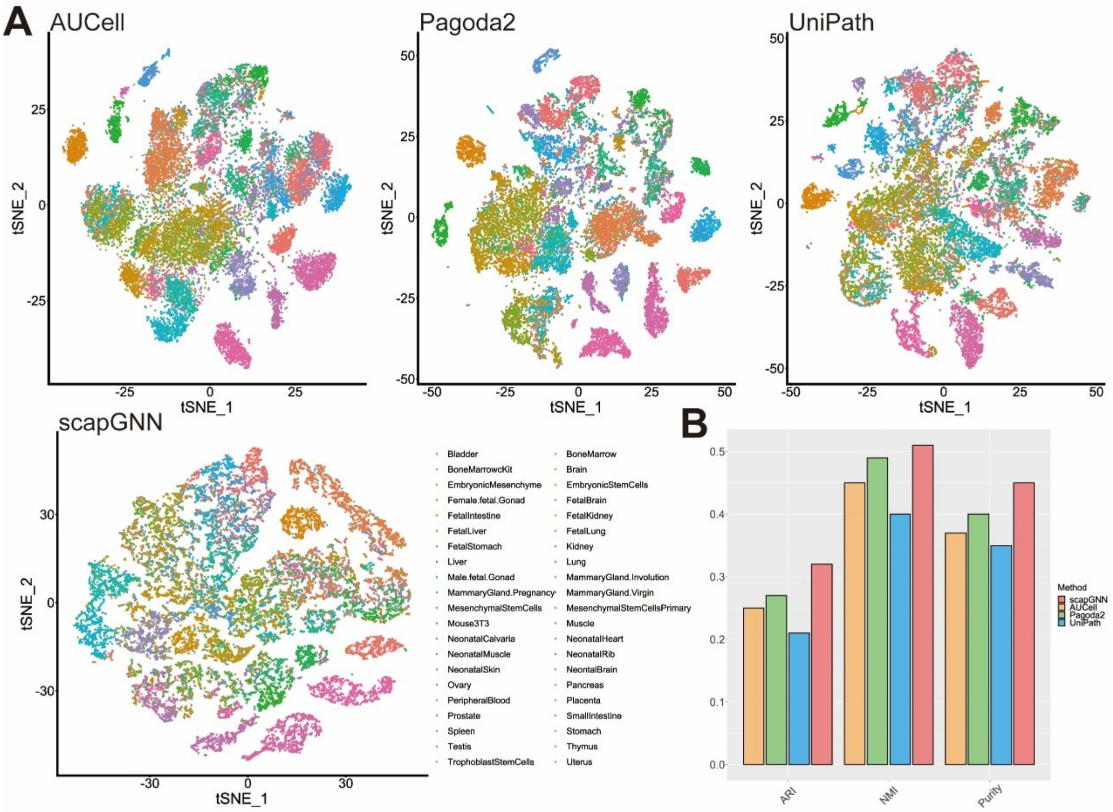

S11 Fig. Evaluation of performance on large-scale single-cell data. (**A**) Cell clustering tSNE of AUCell, Pagoda2, UniPath, and scapGNN on the mouse cell atlas dataset. (**B**) Bar graph of cell clustering accuracy indicators (ARI, NMI, and purity). The data underlying this figure can be found in S7 Data.
