# Supplementary material for: scapGNN: A graph neural network–based framework for active pathway and gene module inference from single-cell multi-omics data
Source: PLoS Biol. 2023 Nov 13;21(11):e3002369. doi: 10.1371/journal.pbio.3002369 (PMC10681325; doi:10.1371/journal.pbio.3002369)
Supplement: S12 Fig — The data underlying this figure can be found in S8 Data. (PDF) [file pbio.3002369.s013.pdf]

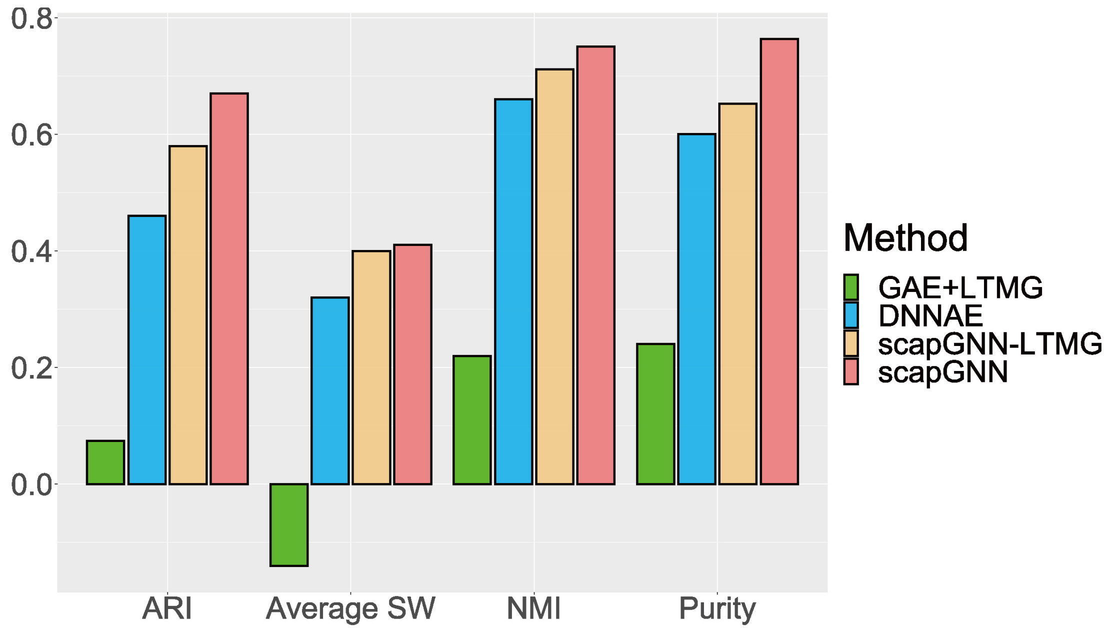

S12 Fig. Evaluation of scapGNN uniformity by ablation experiments on cell type dataset. The data underlying this figure can be found in S8 Data.
