# Supplementary material for: scapGNN: A graph neural network–based framework for active pathway and gene module inference from single-cell multi-omics data
Source: PLoS Biol. 2023 Nov 13;21(11):e3002369. doi: 10.1371/journal.pbio.3002369 (PMC10681325; doi:10.1371/journal.pbio.3002369)
Supplement: S13 Fig — (A) Score distribution of T-cell receptor signaling pathway activity for T cells with T-cell receptor signaling pathway ranked in the top 1 to 5. (B) Score distribution of B cell receptor signaling pathway activity for B cells with B cell receptor signaling pathway ranked in the top 1 to 5. Seurat was used to identify the top 5 B (C) and T (D) cell marker pathways. P_val_adj, adjusted p-value. (PDF) [file pbio.3002369.s014.pdf]

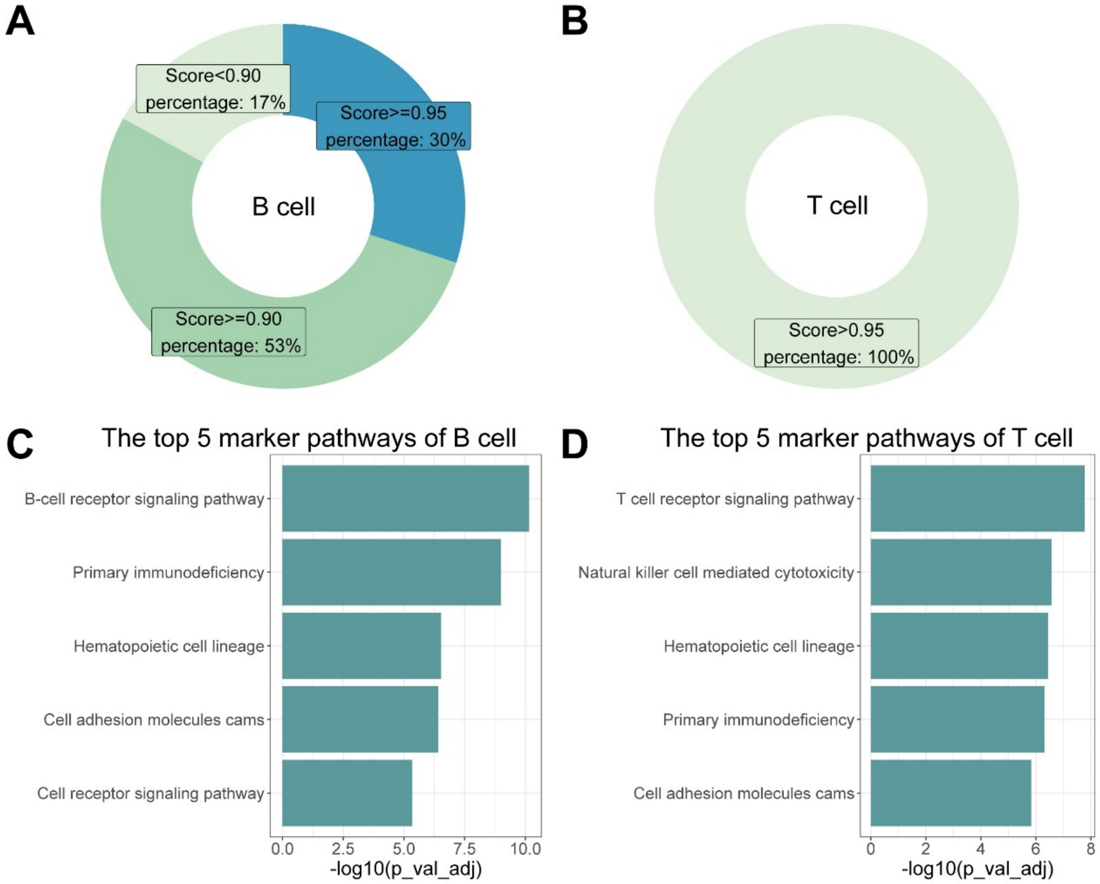

S13 Fig. Pathway score distribution and cell marker pathways in the T-cell and B-cell datasets. (**A**) Score distribution of T-cell receptor signaling pathway activity for T cells with T-cell receptor signaling pathway ranked in the top one to five. (**B**) Score distribution of B-cell receptor signaling pathway activity for B cells with B-cell receptor signaling pathway ranked in the top one to five. Seurat was used to identify the top five B (**C**) and T (**D**) cell marker pathways. P_val_adj, adjusted p-value.
