# Supplementary material for: scapGNN: A graph neural network–based framework for active pathway and gene module inference from single-cell multi-omics data
Source: PLoS Biol. 2023 Nov 13;21(11):e3002369. doi: 10.1371/journal.pbio.3002369 (PMC10681325; doi:10.1371/journal.pbio.3002369)
Supplement: S14 Fig — (A) Proportion of T cells with T-cell receptor signaling pathway or B cells with B cell receptor signaling pathway appeared in the top 5 enriched terms when T cells or B cells were grouped with epithelial cells in the T cell and B cell datasets. (B) Proportion of T cells with T-cell receptor signaling pathway or B cells with B cell receptor signaling pathway appeared in the top 5 enriched terms when T cells or B cells were grouped with monocytes in the scRNA-seq data of the PBMC multi-omics dataset. Untreated means that the original data were used. The data underlying this figure can be found in S2 Data. (PDF) [file pbio.3002369.s015.pdf]

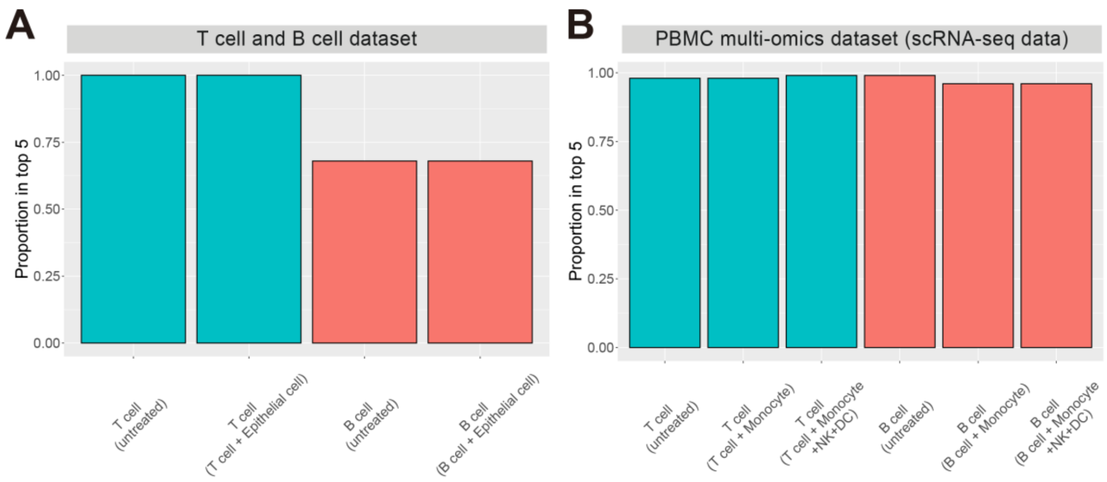

S14 Fig. Stability analysis of the scapGNN identity marker pathway in the scRNA-seq data. (**A**) Proportion of T cells with T-cell receptor signaling pathway or B cells with B-cell receptor signaling pathway appeared in the top five enriched terms when T cells or B cells were grouped with epithelial cells in the T-cell and B-cell datasets. (**B**) Proportion of T cells with T-cell receptor signaling pathway or B cells with B-cell receptor signaling pathway appeared in the top five enriched terms when T cells or B cells were grouped with monocytes in the scRNA-seq data of the PBMC multi-omics dataset. Untreated means that the original data were used. The data underlying this figure can be found in S2 Data.
