# Supplementary material for: scapGNN: A graph neural network–based framework for active pathway and gene module inference from single-cell multi-omics data
Source: PLoS Biol. 2023 Nov 13;21(11):e3002369. doi: 10.1371/journal.pbio.3002369 (PMC10681325; doi:10.1371/journal.pbio.3002369)
Supplement: S15 Fig — Using marker genes of the ESC as gold standards, ROC curves of ESC-associated gene module identified by scapGNN (A) or genomap (B) in the ESC dataset with different strengths of dropout noise. The data underlying this figure can be found in S2 Data. (PDF) [file pbio.3002369.s016.pdf]

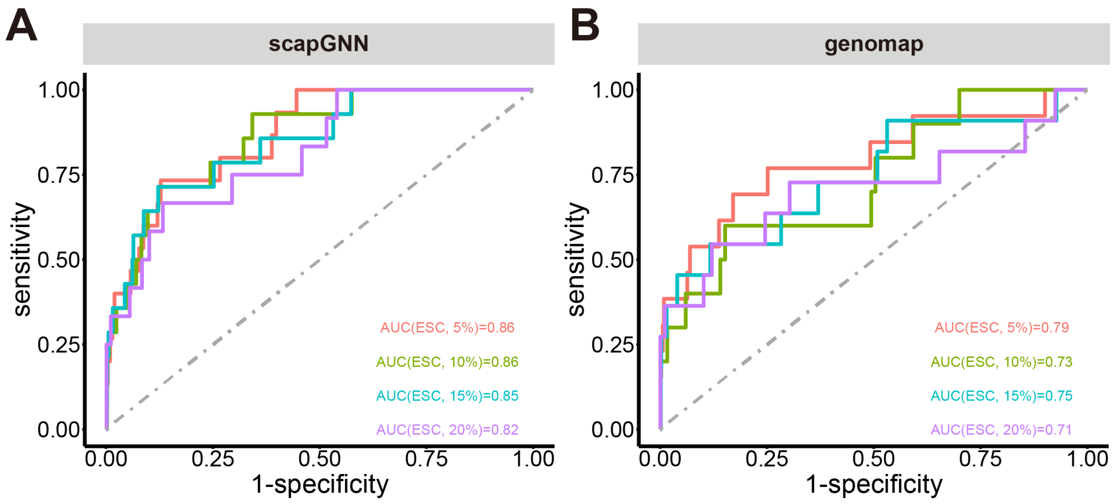

S15 Fig. Robustness analysis of the gene modules. Using marker genes of the ESC as gold standards, ROC curves of ESC-associated gene module identified by scapGNN (**A**) or genomap (**B**) in the ESC dataset with different strengths of dropout noise. The data underlying this figure can be found in S2 Data.
