# Supplementary material for: scapGNN: A graph neural network–based framework for active pathway and gene module inference from single-cell multi-omics data
Source: PLoS Biol. 2023 Nov 13;21(11):e3002369. doi: 10.1371/journal.pbio.3002369 (PMC10681325; doi:10.1371/journal.pbio.3002369)
Supplement: S16 Fig — The data underlying this figure can be found in S8 Data. (PDF) [file pbio.3002369.s017.pdf]

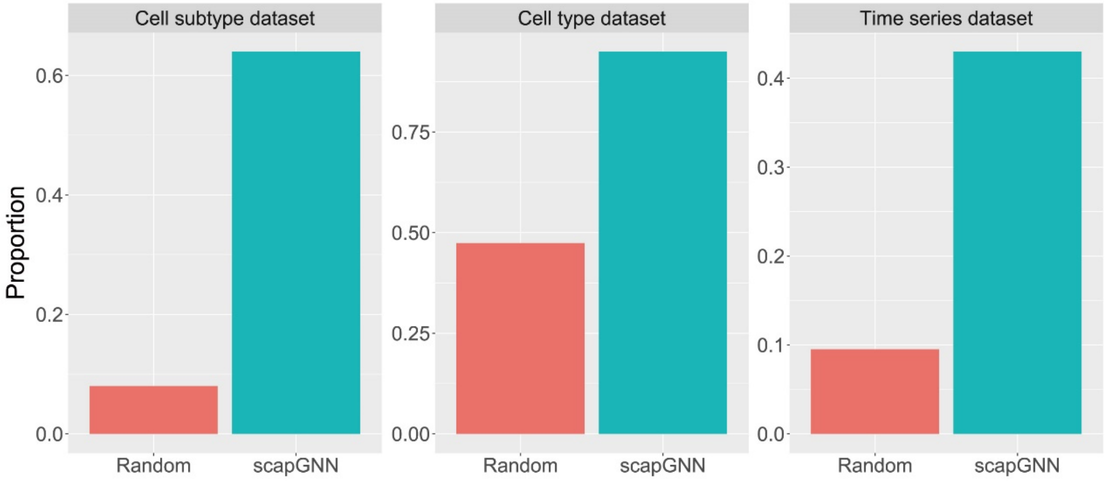

S16 Fig. Functionally modular evaluation for cell phenotype–associated gene modules of activated stellate cells in the cell type dataset, eProg1b cells in the cell subtype dataset, and 36-h cells in the time series dataset. The data underlying this figure can be found in S8 Data.
