# Supplementary material for: scapGNN: A graph neural network–based framework for active pathway and gene module inference from single-cell multi-omics data
Source: PLoS Biol. 2023 Nov 13;21(11):e3002369. doi: 10.1371/journal.pbio.3002369 (PMC10681325; doi:10.1371/journal.pbio.3002369)
Supplement: S17 Fig — UMAP visualizations of the AUCell (A), Pagoda2 (B), and UniPath (C) on cell subtype datasets with different strengths of dropout noise. (D) AUC between the zero-valued rates and 3 cell clustering accuracy (ARI, NMI, and SW) quantification indicators on 16 scRNA-seq data sets. (E) Proportion of ESC cells with corresponding marker gene set in the top 1 to 5 pathway scores using the 4 pathway enrichment methods under different strengths of dropout noise on the ESC dataset. The data underlying this figure can be found in S3 Data. ARI, adjusted rand index; AUC, area under the recovery curve; ESC, embryonic stem cell; NMI, normalized mutual information; scRNA-seq, single-cell RNA sequencing; SW, silhouette width; UMAP, Uniform Manifold Approximation and Projection. (PDF) [file pbio.3002369.s018.pdf]

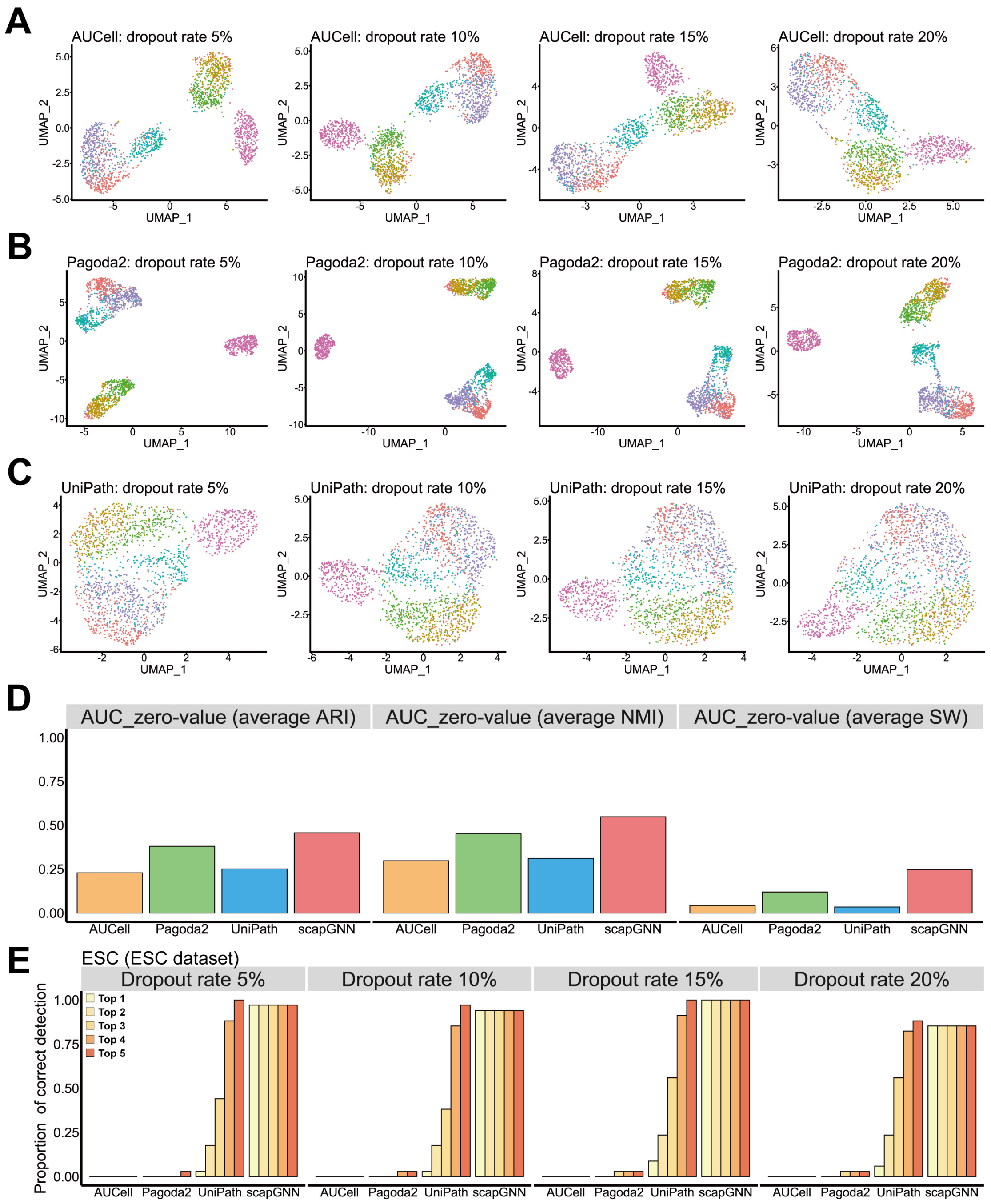

S17 Fig. Robustness evaluation. UMAP visualizations of the AUCell (**A**), Pagoda2 (**B**), and UniPath (**C**) on cell subtype datasets with different strengths of dropout noise. (**D**) AUC between the zero-valued rates and three cell clustering accuracy (ARI, NMI, and SW) quantification indicators on 16 scRNA-seq data sets. (**E**) Proportion of ESC cells with corresponding marker gene set in the top one to five pathway scores using the four pathway enrichment methods under different strengths of dropout noise on the ESC dataset. The data underlying this figure can be found in S3 Data.
