# Supplementary material for: scapGNN: A graph neural network–based framework for active pathway and gene module inference from single-cell multi-omics data
Source: PLoS Biol. 2023 Nov 13;21(11):e3002369. doi: 10.1371/journal.pbio.3002369 (PMC10681325; doi:10.1371/journal.pbio.3002369)
Supplement: S18 Fig — (A) Proportion of astrocytes with corresponding marker gene set in the top 1 to 5 pathway scores using the 4 pathway enrichment methods under different strengths of dropout noise. (B) The proportion of astrocytes with the marker gene set of astrocytes appeared in the top 5 enriched terms when astrocytes were grouped with layer 6b (L6b) or vasoactive intestinal polypeptide (Vip) cells. Untreated means that the original data were used. The data underlying this figure can be found in S4 Data. (PDF) [file pbio.3002369.s019.pdf]

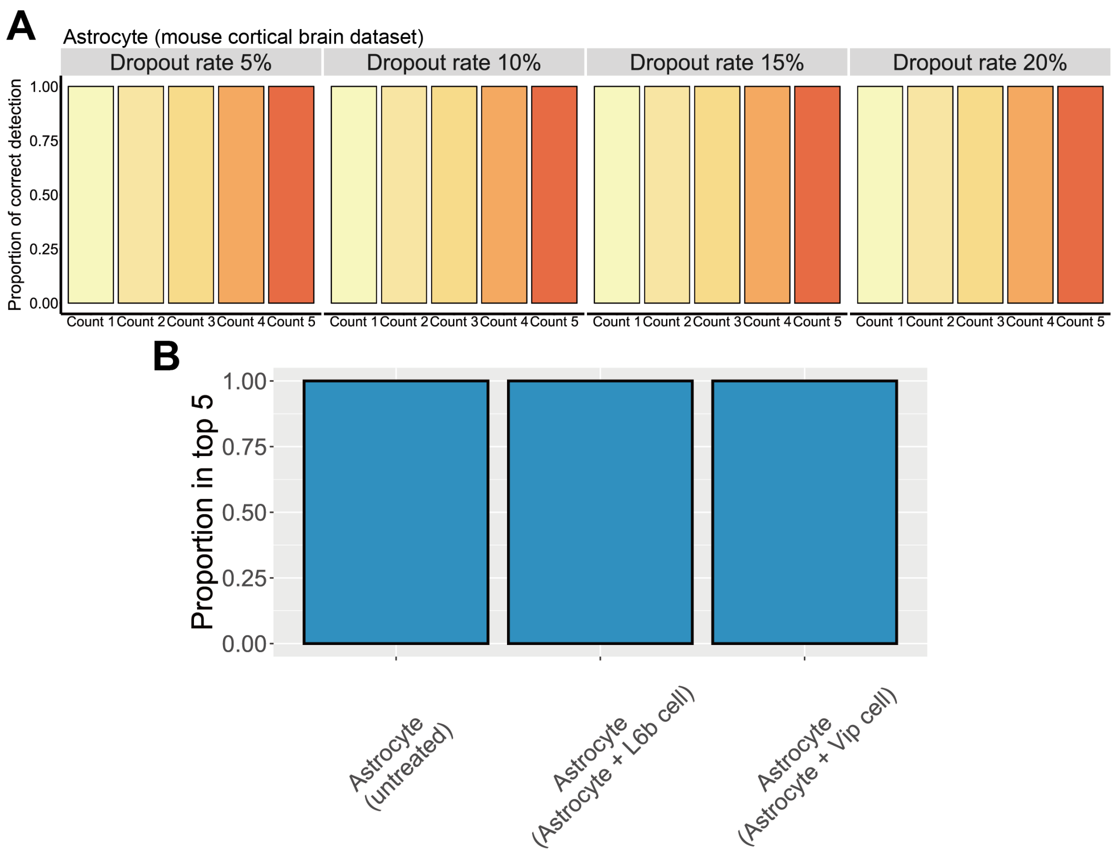

S18 Fig. Evaluation of the stability of scapGNN in scATAC-seq data of the mouse cortical brain dataset for pathway activity scoring. (**A**) Proportion of astrocytes with corresponding marker gene set in the top one to five pathway scores using the four pathway enrichment methods under different strengths of dropout noise. (**B**) The proportion of astrocytes with the marker gene set of astrocytes appeared in the top five enriched terms when astrocytes were grouped with layer 6b (L6b) or vasoactive intestinal polypeptide (Vip) cells. Untreated means that the original data were used. The data underlying this figure can be found in S4 Data.
