# Supplementary material for: scapGNN: A graph neural network–based framework for active pathway and gene module inference from single-cell multi-omics data
Source: PLoS Biol. 2023 Nov 13;21(11):e3002369. doi: 10.1371/journal.pbio.3002369 (PMC10681325; doi:10.1371/journal.pbio.3002369)
Supplement: S21 Fig — UMAP visualization of the gene expression matrix from scRNA-seq, the gene activity score matrix from scATAC-seq, and the pathway activity score matrix from single-cell multi-omics integration of scapGNN for the mouse brain cortex dataset (A), mouse skin dataset (B), and PBMC multi-omics dataset (C). (D) Bar graph of the 3 cell clustering accuracy indicators for scapGNN, and the state-of-the-art single-cell multi-omics integration methods on the mouse brain cortex dataset. (E) Bar graph of the cell clustering accuracy indicator for single-cell transcriptomic and single-cell epigenomic data from the mouse brain cortex, mouse skin, and PBMC multi-omics datasets. The data underlying this figure can be found in S5 Data. PBMC, peripheral blood mononuclear cell; scATAC-seq, single-cell ATAC sequencing; scRNA-seq, single-cell RNA sequencing; UMAP, Uniform Manifold Approximation and Projection. (PDF) [file pbio.3002369.s022.pdf]

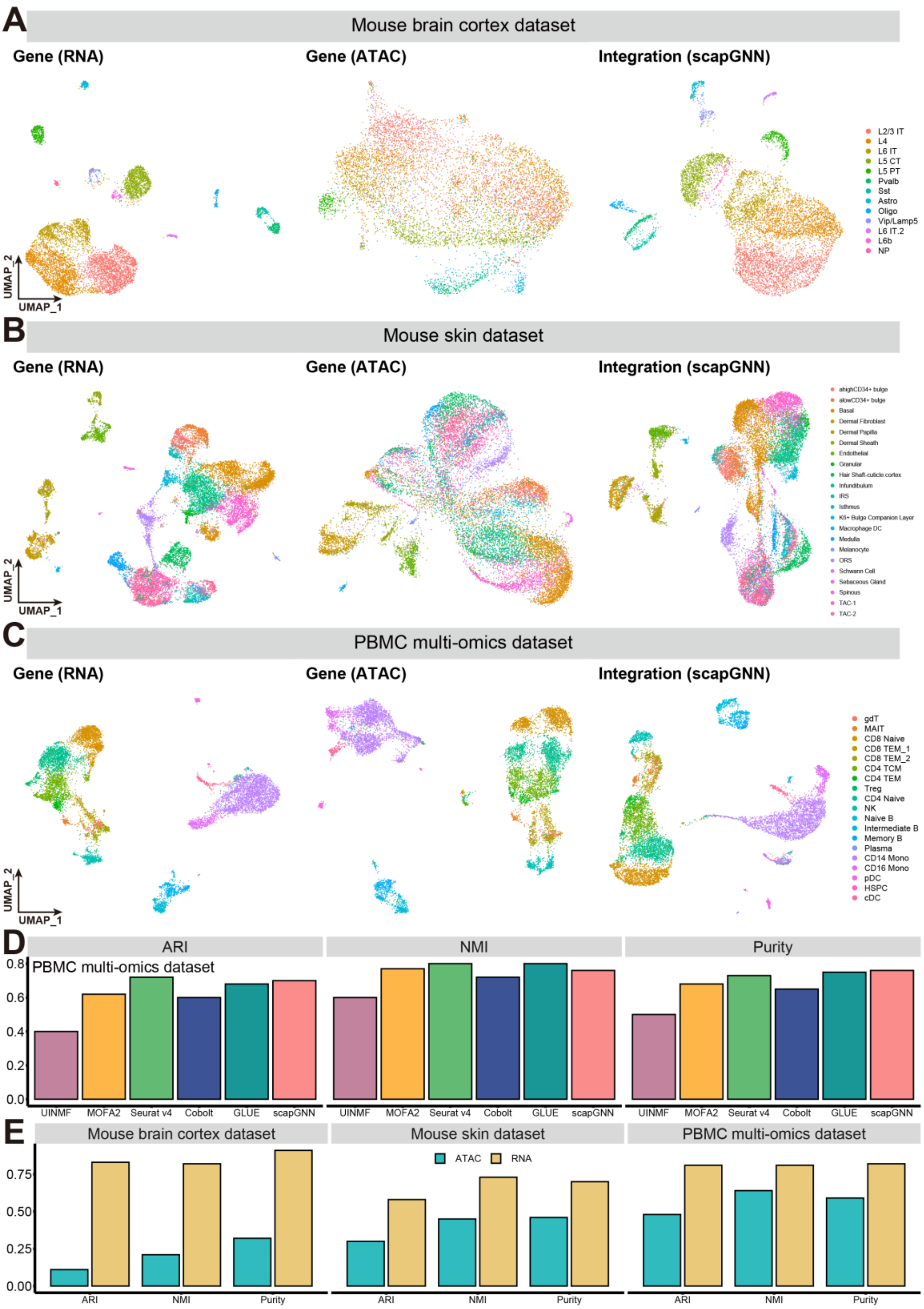

S21 Fig. Performance of scapGNN for cell clustering in single-cell multi-omics data integration. UMAP visualization of the gene expression matrix from scRNA-seq, the gene activity score matrix from scATAC-seq, and the pathway activity score matrix from single-cell multi-omics integration of scapGNN for the mouse brain cortex dataset (**A**), mouse skin dataset (**B**), and PBMC multi-omics dataset (**C**). (**D**)

Bar graph of the three cell clustering accuracy indicators for scapGNN, and the state-of-the-art single-cell multi-omics integration methods on the mouse brain cortex dataset. (E) Bar graph of the cell clustering accuracy indicator for single-cell transcriptomic and single-cell epigenomic data from the mouse brain cortex, mouse skin, and PBMC multi-omics datasets. The data underlying this figure can be found in S5 Data.
