# Supplementary material for: scapGNN: A graph neural network–based framework for active pathway and gene module inference from single-cell multi-omics data
Source: PLoS Biol. 2023 Nov 13;21(11):e3002369. doi: 10.1371/journal.pbio.3002369 (PMC10681325; doi:10.1371/journal.pbio.3002369)
Supplement: S22 Fig — Latent embedding of scDART and visualization using PCA on the mouse skin dataset. Cells are colored with cell type (A) and inferred pseudotime (B). Red arrows are TAC populations pointing to the medulla, IRS, and hair shaft cuticle/cortex cells. The data underlying this figure can be found in S5 Data. IRS, inner root sheath; PCA, principal component analysis; TAC, transit-amplifying cell. (PDF) [file pbio.3002369.s023.pdf]

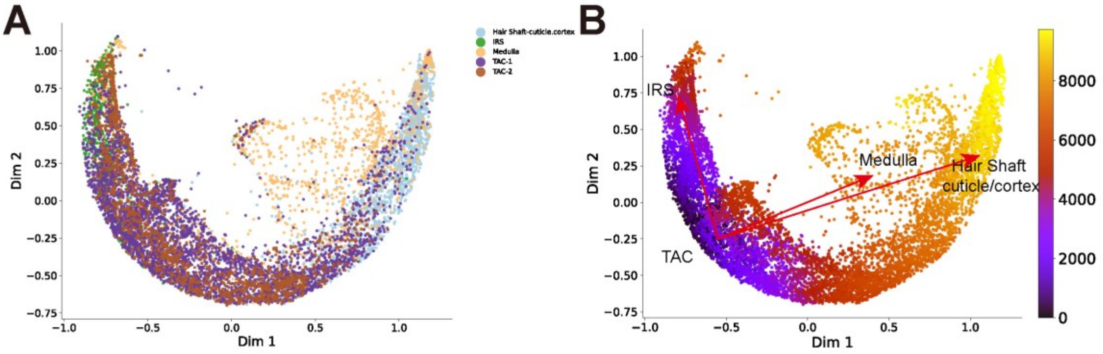

S22 Fig. Performance of scapGNN for pseudotime inference in single-cell multi-omics data integration. Latent embedding of scDART and visualization using principal component analysis (PCA) on the mouse skin dataset. Cells are colored with cell type (**A**) and inferred pseudotime (**B**). Red arrows are TAC populations pointing to the medulla, IRS, and hair shaft cuticle/cortex cells. The data underlying this figure can be found in S5 Data.
