# Supplementary material for: scapGNN: A graph neural network–based framework for active pathway and gene module inference from single-cell multi-omics data
Source: PLoS Biol. 2023 Nov 13;21(11):e3002369. doi: 10.1371/journal.pbio.3002369 (PMC10681325; doi:10.1371/journal.pbio.3002369)
Supplement: S23 Fig — (A) Proportion of cells with T- or B cell receptor signaling pathway in the top 5 pathways for the PBMC multi-omics dataset. (B) ROC curves of gene modules of T and B cells in the PBMC multi-omics dataset with T- and B cell receptor signaling pathway genes as gold standards. (C) Proportion of cells that detected the correct marker gene sets using scapGNN in the top 1 to 5 of the single-cell multi-omics–supported pathway activity scores. (D) ROC curves of gene modules of the endothelial cells and GM12878 with marker gene sets for each cell type as gold standards. The endothelial cells are from the mouse skin dataset, and GM12878 is from the GM12878 dataset. The data underlying this figure can be found in S5 Data. (PDF) [file pbio.3002369.s024.pdf]

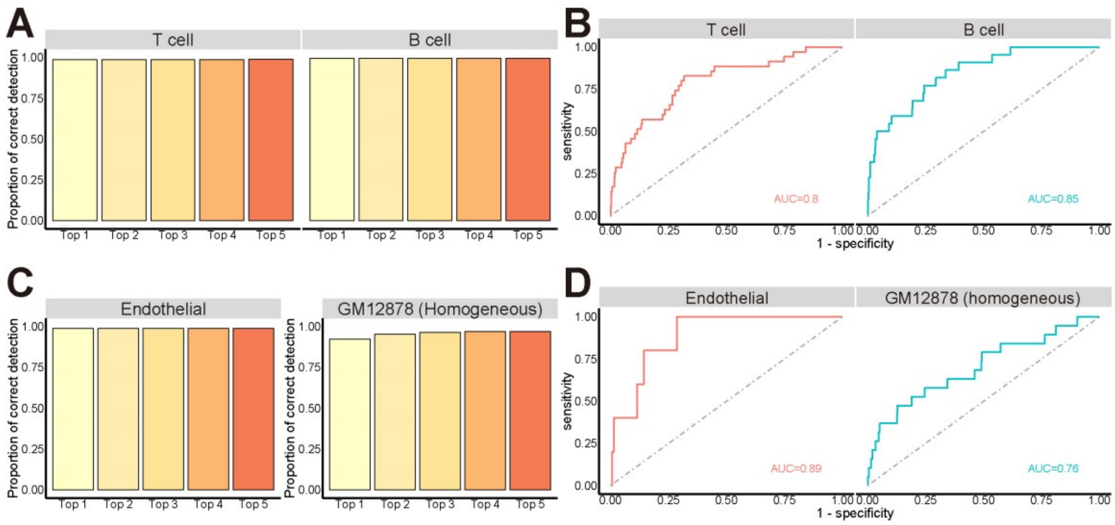

S23 Fig. scapGNN was capable of accurately identifying active pathways in single-cell multi-omics data. (**A**) Proportion of cells with T- or B-cell receptor signaling pathway in the top five pathways for the PBMC multi-omics dataset. (**B**) ROC curves of gene modules of T and B cells in the PBMC multi-omics dataset with T- and B-cell receptor signaling pathway genes as gold standards. (**C**) Proportion of cells that detected the correct marker gene sets using scapGNN in the top one to five of the single-cell multi-omics–supported pathway activity scores. (**D**) ROC curves of gene modules of the endothelial cells and GM12878 with marker gene sets for each cell type as gold standards. The endothelial cells are from the mouse skin dataset, and GM12878 is from the GM12878 dataset. The data underlying this figure can be found in S5 Data.
