# Supplementary material for: scapGNN: A graph neural network–based framework for active pathway and gene module inference from single-cell multi-omics data
Source: PLoS Biol. 2023 Nov 13;21(11):e3002369. doi: 10.1371/journal.pbio.3002369 (PMC10681325; doi:10.1371/journal.pbio.3002369)
Supplement: S24 Fig — The proportion of T cells with T-cell receptor signaling pathway appeared in the top 5 enriched terms when T cells were grouped with monocytes. Untreated means that the raw data were used. The data underlying this figure can be found in S5 Data. (PDF) [file pbio.3002369.s025.pdf]

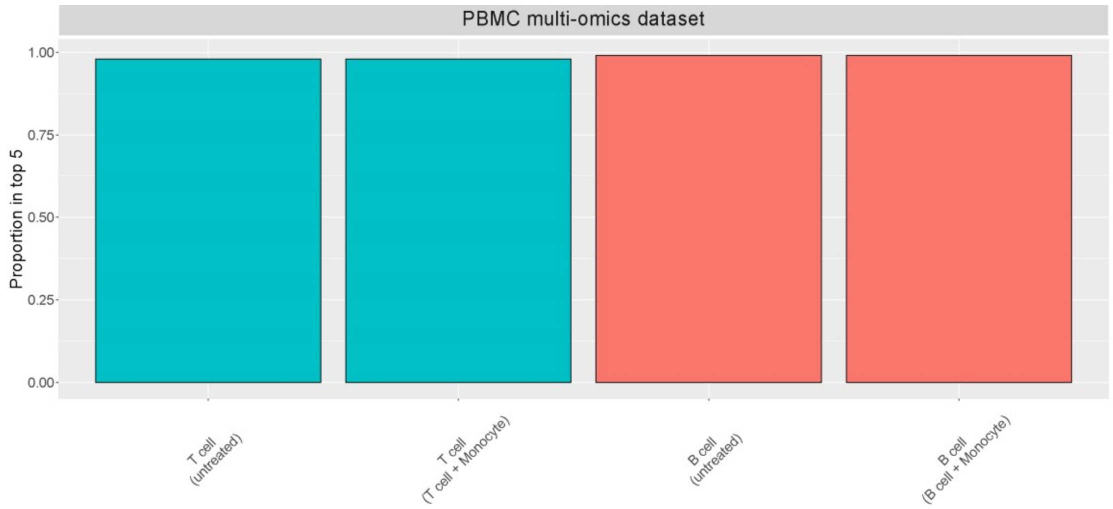

S24 Fig. Stability analysis of the scapGNN in identifying the marker pathway in the single-cell multi-omics integration. The proportion of T cells with T-cell receptor signaling pathway appeared in the top five enriched terms when T cells were grouped with monocytes. Untreated means that the raw data were used. The data underlying this figure can be found in S5 Data.
