# Supplementary material for: scapGNN: A graph neural network–based framework for active pathway and gene module inference from single-cell multi-omics data
Source: PLoS Biol. 2023 Nov 13;21(11):e3002369. doi: 10.1371/journal.pbio.3002369 (PMC10681325; doi:10.1371/journal.pbio.3002369)
Supplement: S25 Fig — Violin plots of Gli2 (A) and Gli3 (B) activity in scRNA-seq and scATAC-seq data from the mouse skin dataset. (C) UMAP plots colored by the single-cell multi-omics supported pathway activity scores of the hedgehog signaling pathway. (D) Network of cell phenotype–associated gene modules during the proliferation of TACs in hair follicle tissue. Red boxes mark the locations of Gli2 and Gli3. The data underlying this figure can be found in S5 Data. scATAC-seq, single-cell ATAC sequencing; scRNA-seq, single-cell RNA sequencing; TAC, transit-amplifying cell; UMAP, Uniform Manifold Approximation and Projection. (PDF) [file pbio.3002369.s026.pdf]

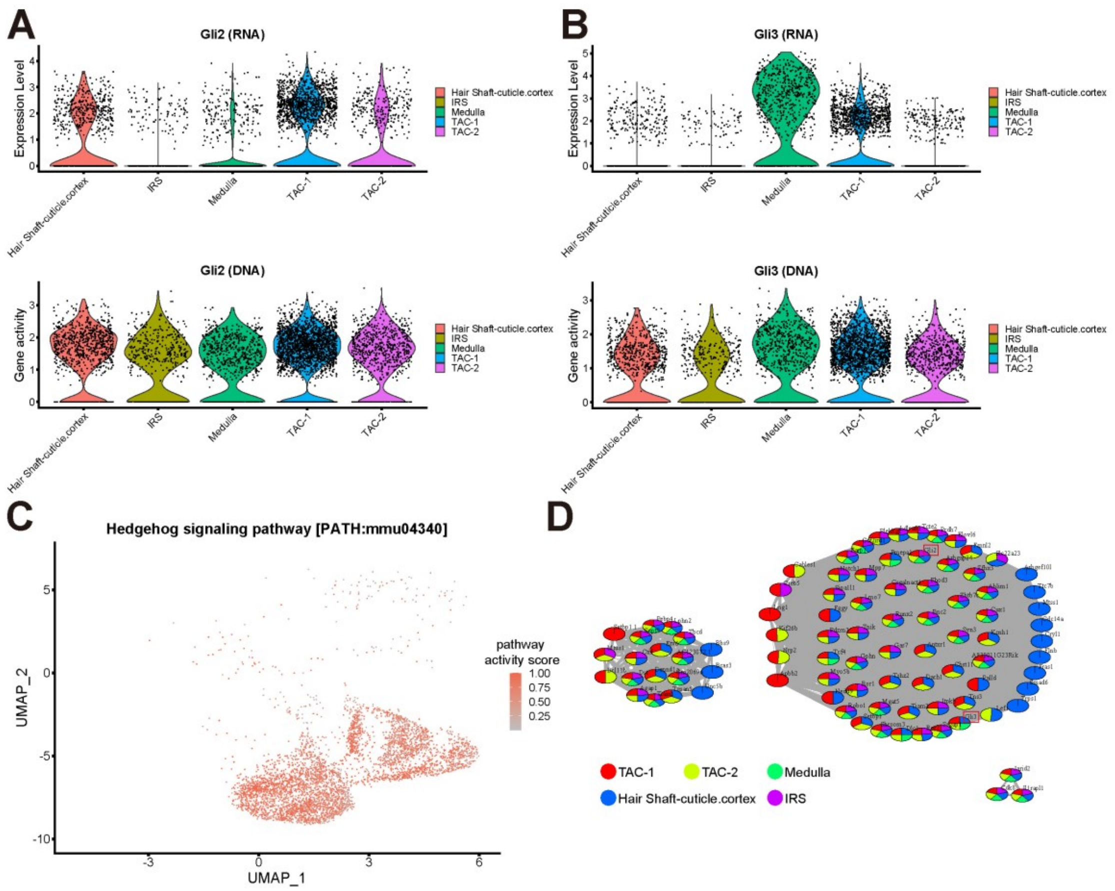

S25 Fig. Developmental process–dependent and hedgehog signaling pathways in TACs. Violin plots of Gli2 (**A**) and Gli3 (**B**) activity in scRNA-seq and scATAC-seq data from the mouse skin dataset. (**C**) UMAP plots colored by the single-cell multi-omics supported pathway activity scores of the hedgehog signaling pathway. (**D**) Network of cell phenotype–associated gene modules during the proliferation of TACs in hair follicle tissue. Red boxes mark the locations of Gli2 and Gli3. The data underlying this figure can be found in S5 Data.
