# Supplementary material for: scapGNN: A graph neural network–based framework for active pathway and gene module inference from single-cell multi-omics data
Source: PLoS Biol. 2023 Nov 13;21(11):e3002369. doi: 10.1371/journal.pbio.3002369 (PMC10681325; doi:10.1371/journal.pbio.3002369)
Supplement: S26 Fig — The data underlying this figure can be found in S6 Data. (PDF) [file pbio.3002369.s027.pdf]

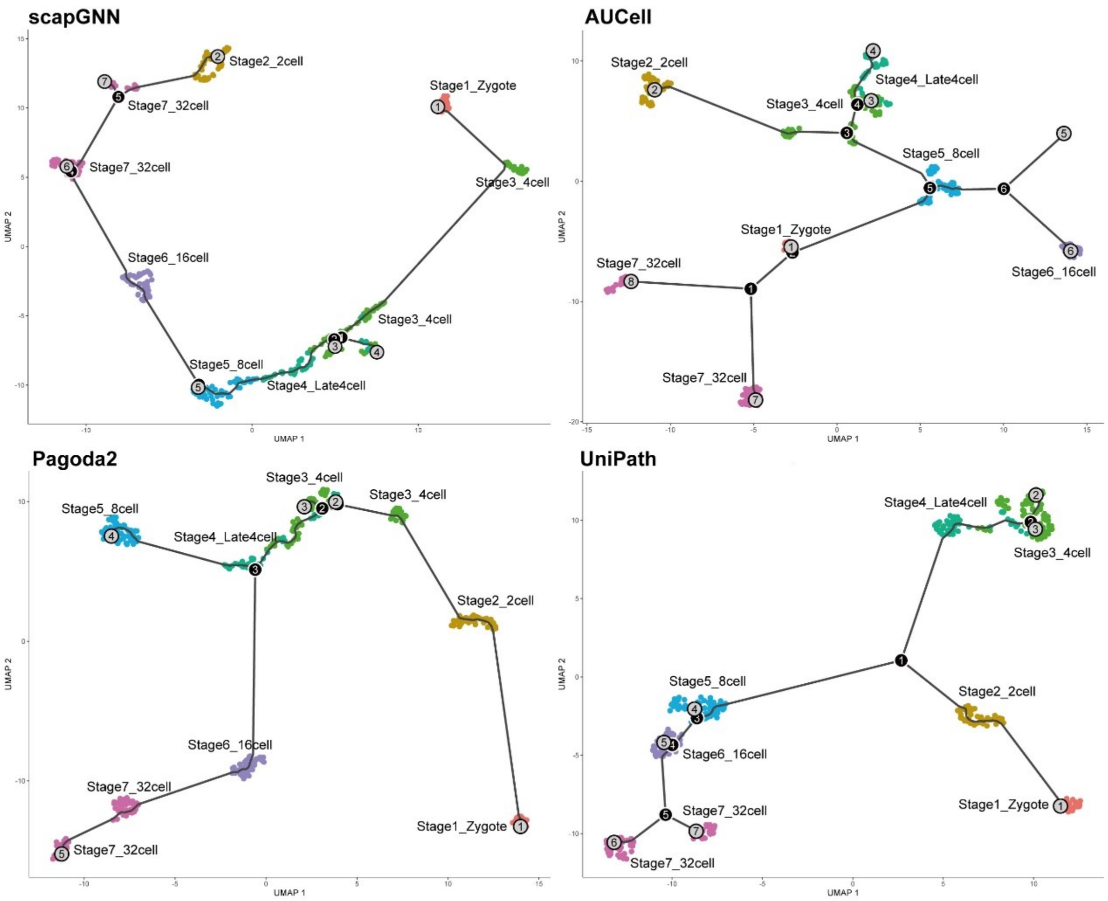

S26 Fig. Cell differentiation trajectory analysis of mouse early embryo development using pathway activity scores from scapGNN, AUCell, Pagoda2, and UniPath. The data underlying this figure can be found in S6 Data.
