# Supplementary material for: scapGNN: A graph neural network–based framework for active pathway and gene module inference from single-cell multi-omics data
Source: PLoS Biol. 2023 Nov 13;21(11):e3002369. doi: 10.1371/journal.pbio.3002369 (PMC10681325; doi:10.1371/journal.pbio.3002369)
Supplement: S27 Fig — (A) Gene association network of oxidative phosphorylation pathway in the early pachytene and steps 3–4 spermatids. The node size reflects the importance of the genes, and the width of the edges between nodes indicates the strength of association between genes in the network. (B) Dynamic expression of the PPAR signaling pathway during early embryonic development as counted by the EmExplorer database. (C) Cell community association network of human testis data. The width of the edges indicates the strength of association between cell communities. (D) Network of cell type–associated gene modules between Sertoli cells and spermatogenic cells in the human testis dataset. In the network, the sector area of a node indicates the strength of association between a gene and a cell phenotype, and the width of the edges between nodes indicates the strength of association between genes. The data underlying this figure can be found in S6 Data. (PDF) [file pbio.3002369.s028.pdf]

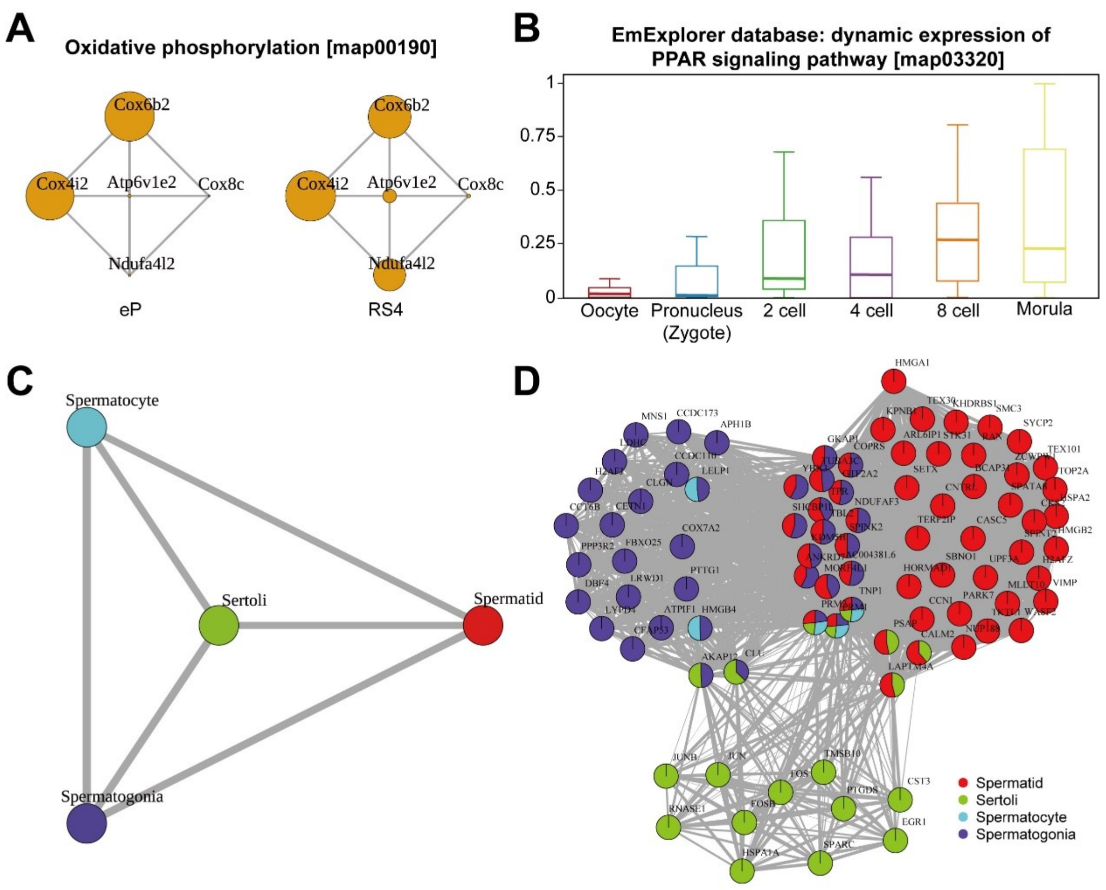

S27 Fig. An extension of the application in reproductive biology. (**A**) Gene association network of oxidative phosphorylation pathway in the early pachytene and steps 3–4 spermatids. The node size reflects the importance of the genes, and the width of the edges between nodes indicates the strength of association between genes in the network. (**B**) Dynamic expression of the PPAR signaling pathway during early embryonic development as counted by the EmExplorer database. (**C**) Cell community association network of human testis data. The width of the edges indicates the strength of association between cell communities. (**D**) Network of cell type–associated gene modules between Sertoli cells and spermatogenic cells in the human testis dataset. In the network, the sector area of a node indicates the strength of association between a gene and a cell phenotype, and the width of the edges between nodes indicates the strength of association between genes. The data underlying this figure can be found in S6 Data.
