# Supplementary material for: scapGNN: A graph neural network–based framework for active pathway and gene module inference from single-cell multi-omics data
Source: PLoS Biol. 2023 Nov 13;21(11):e3002369. doi: 10.1371/journal.pbio.3002369 (PMC10681325; doi:10.1371/journal.pbio.3002369)
Supplement: S28 Fig — Rbakdn (A) and Spata33 (B) were the 2 genes with the largest association scores with Fabp9. Cypt12 (C) and Hmgb4 (D), 2 genes with smaller association scores with Fabp9, served as controls. The data underlying this figure can be found in S6 Data. (PDF) [file pbio.3002369.s029.pdf]

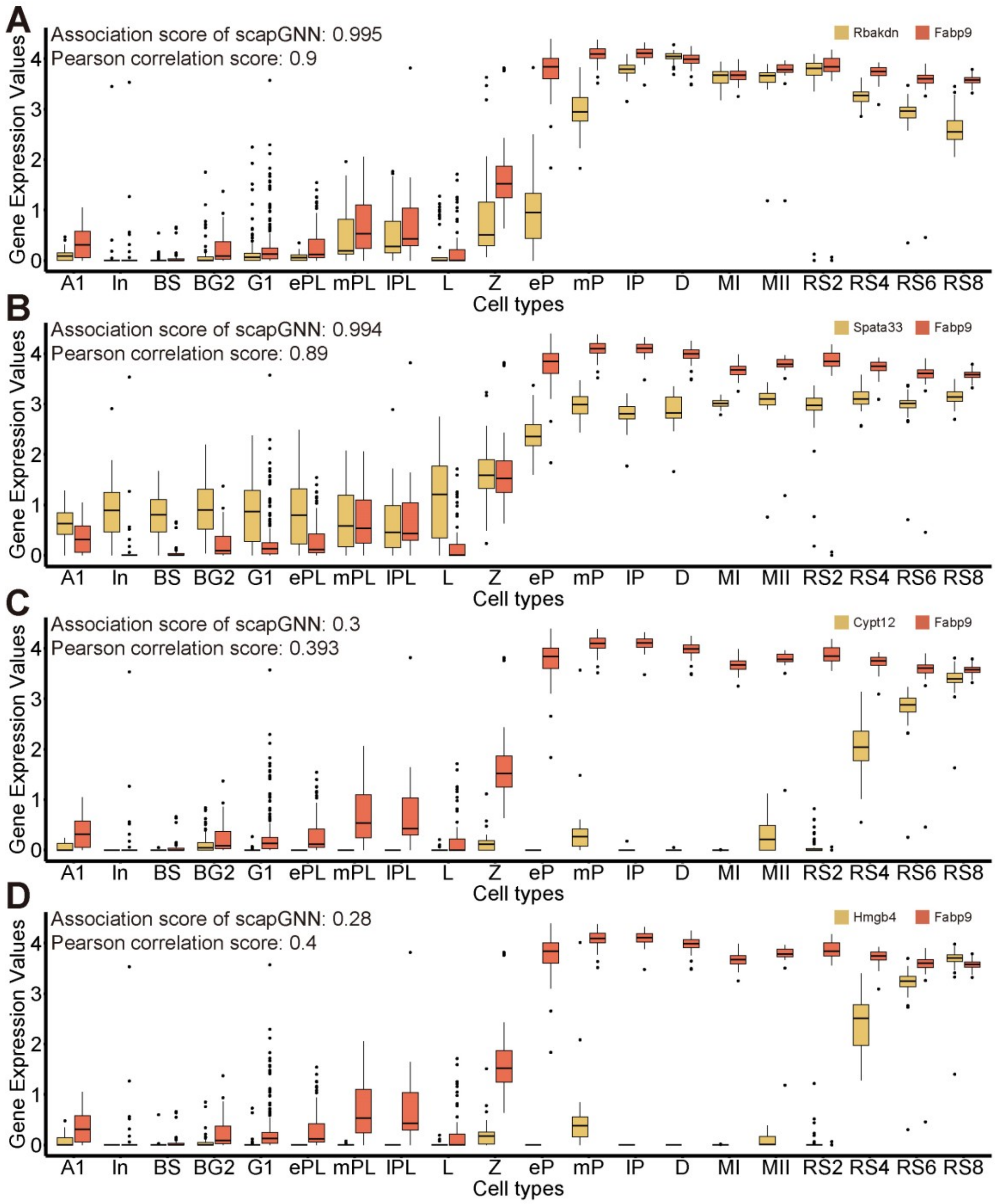

S28 Fig. Box plots of expression values of genes associated with *Fabp9* in each stage of spermatogenesis. *Rbakdn* (**A**) and *Spata33* (**B**) were the two genes with the largest association scores with *Fabp9*. *Cypt12* (**C**) and *Hmgb4* (**D**), two genes with smaller association scores with *Fabp9*, served as controls. The data underlying this figure can be found in S6 Data.
