# Supplementary material for: scapGNN: A graph neural network–based framework for active pathway and gene module inference from single-cell multi-omics data
Source: PLoS Biol. 2023 Nov 13;21(11):e3002369. doi: 10.1371/journal.pbio.3002369 (PMC10681325; doi:10.1371/journal.pbio.3002369)
Supplement: S30 Fig — The annotations in the columns of the heat map indicate the phenotype to which the cell belongs. The row annotations indicate that the pathway is significantly different in the phenotype of the cells. The data underlying this figure can be found in S8 Data. (PDF) [file pbio.3002369.s031.pdf]

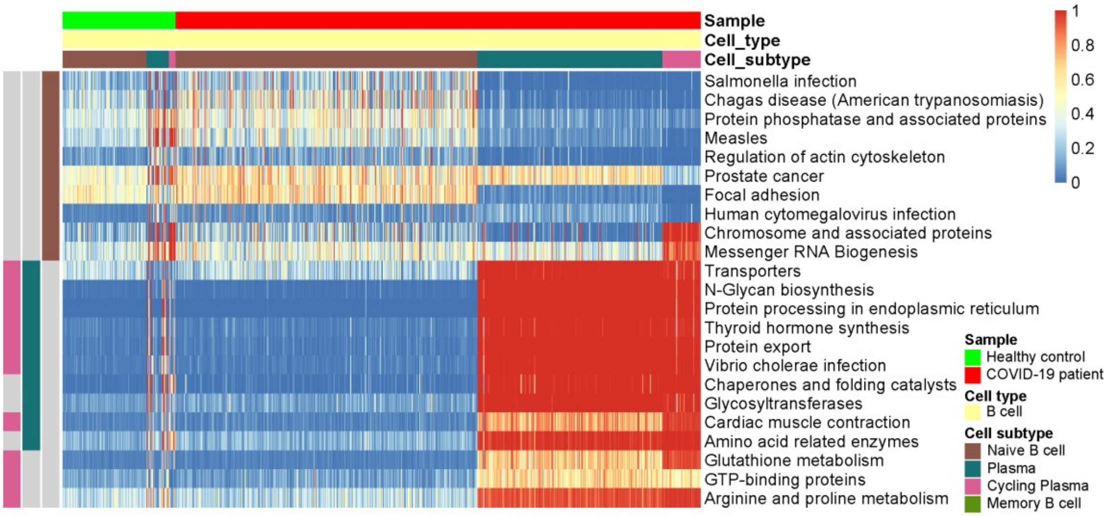

S30 Fig. Heatmap of the activity scores of the B-cell-associated differential pathway between the healthy controls and patients with COVID-19. The annotations in the columns of the heat map indicate the phenotype to which the cell belongs. The row annotations indicate that the pathway is significantly different in the phenotype of the cells. The data underlying this figure can be found in S8 Data.
