# Supplementary material for: scapGNN: A graph neural network–based framework for active pathway and gene module inference from single-cell multi-omics data
Source: PLoS Biol. 2023 Nov 13;21(11):e3002369. doi: 10.1371/journal.pbio.3002369 (PMC10681325; doi:10.1371/journal.pbio.3002369)
Supplement: S31 Fig — (A) Runtime of the GNN module of scapGNN in different cell-scale scRNA-seq datasets. (B) Time (min) of pathway activity scores for scapGNN in different cell-scale scRNA-seq datasets. (C) Time (s) of pathway activity scores for AUCell, Pagoda2, and UniPath in different cell-scale scRNA-seq datasets. The data underlying this figure can be found in S8 Data. (PDF) [file pbio.3002369.s032.pdf]

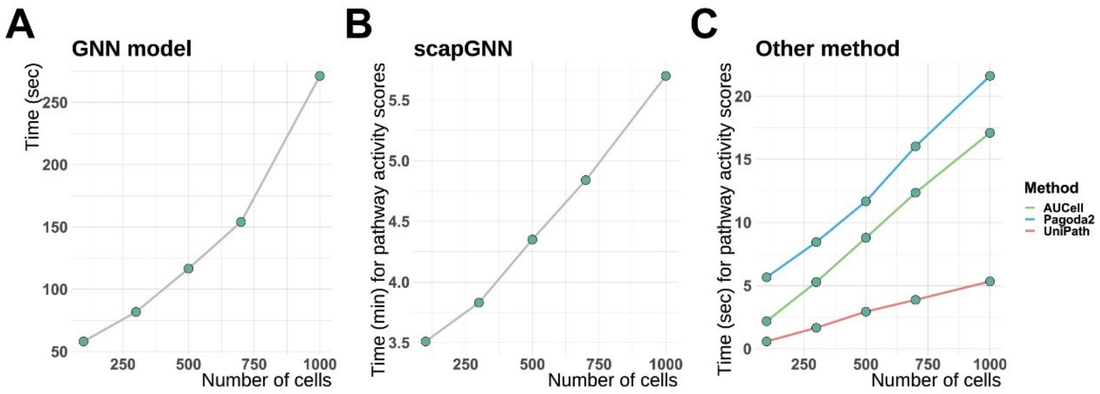

S31 Fig. Evaluation of runtime. (**A**) Runtime of the GNN module of scapGNN in different cell-scale scRNA-seq datasets. (**B**) Time (min) of pathway activity scores for scapGNN in different cell-scale scRNA-seq datasets. (**C**) Time (s) of pathway activity scores for AUCell, Pagoda2, and UniPath in different cell-scale scRNA-seq datasets. The data underlying this figure can be found in S8 Data.
