# Supplementary material for: scapGNN: A graph neural network–based framework for active pathway and gene module inference from single-cell multi-omics data
Source: PLoS Biol. 2023 Nov 13;21(11):e3002369. doi: 10.1371/journal.pbio.3002369 (PMC10681325; doi:10.1371/journal.pbio.3002369)
Supplement: S32 Fig — Genes, initially 2,000 highly variable genes and then increasing in count, were used to calculate individual cell pathway activity scores for the cell type dataset. (A) Cell clustering indicators based on pathway activity scores at different gene numbers. (B) Proportion of endothelial cells with the corresponding marker gene set in the top 5 for different gene counts. The data underlying this figure can be found in S8 Data. (PDF) [file pbio.3002369.s033.pdf]

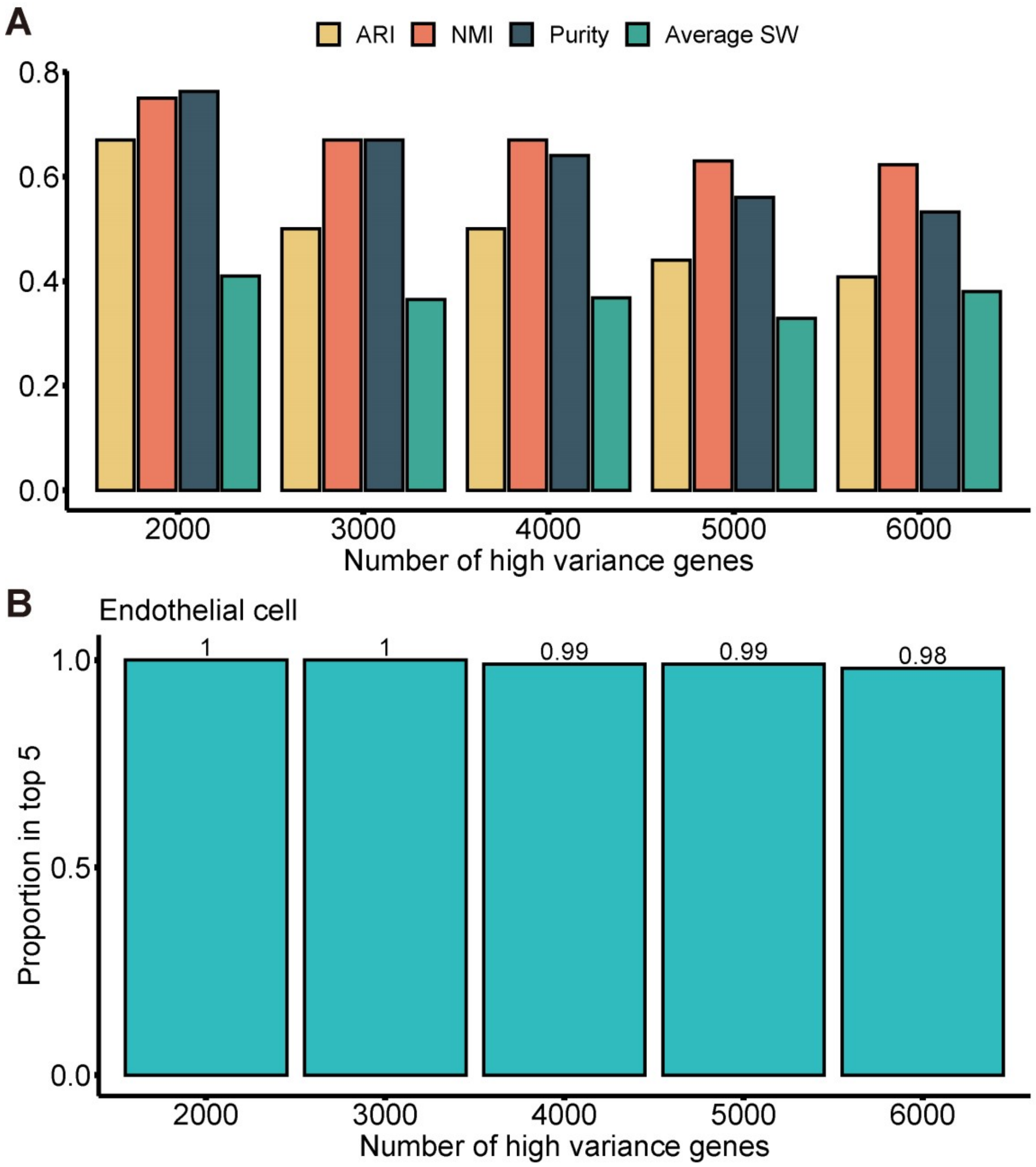

S32 Fig. Effect of increasing the number of genes in heterogeneous data on the performance of scapGNN. Genes, initially 2000 highly variable genes and then increasing in count, were used to calculate individual cell pathway activity scores for the cell type dataset. (**A**) Cell clustering indicators based on pathway activity scores at different gene numbers. (**B**) Proportion of endothelial cells with the corresponding marker gene set in the top five for different gene counts. The data underlying this figure can be found in S8 Data.
