# Supplementary material for: scapGNN: A graph neural network–based framework for active pathway and gene module inference from single-cell multi-omics data
Source: PLoS Biol. 2023 Nov 13;21(11):e3002369. doi: 10.1371/journal.pbio.3002369 (PMC10681325; doi:10.1371/journal.pbio.3002369)
Supplement: S33 Fig — Genes, initially 2,000 highly variable genes and then increasing in count, were used to calculate individual cell pathway activity scores. (A) Proportion of T cells with the corresponding T-cell receptor signaling pathway in the top 5 for different gene counts. (B) Scatter plots show the relationship between the genes in the T-cell receptor signaling pathway and the 8,000 highly variant genes that were screened. (C) Proportion of K562 cells with the corresponding marker gene set in the top 5 for different gene counts. (D) Scatter plots showing the relationship between the genes in the marker gene set of the K562 cells and the 8,000 highly variant genes that were screened. The data underlying this figure can be found in S8 Data. (PDF) [file pbio.3002369.s034.pdf]

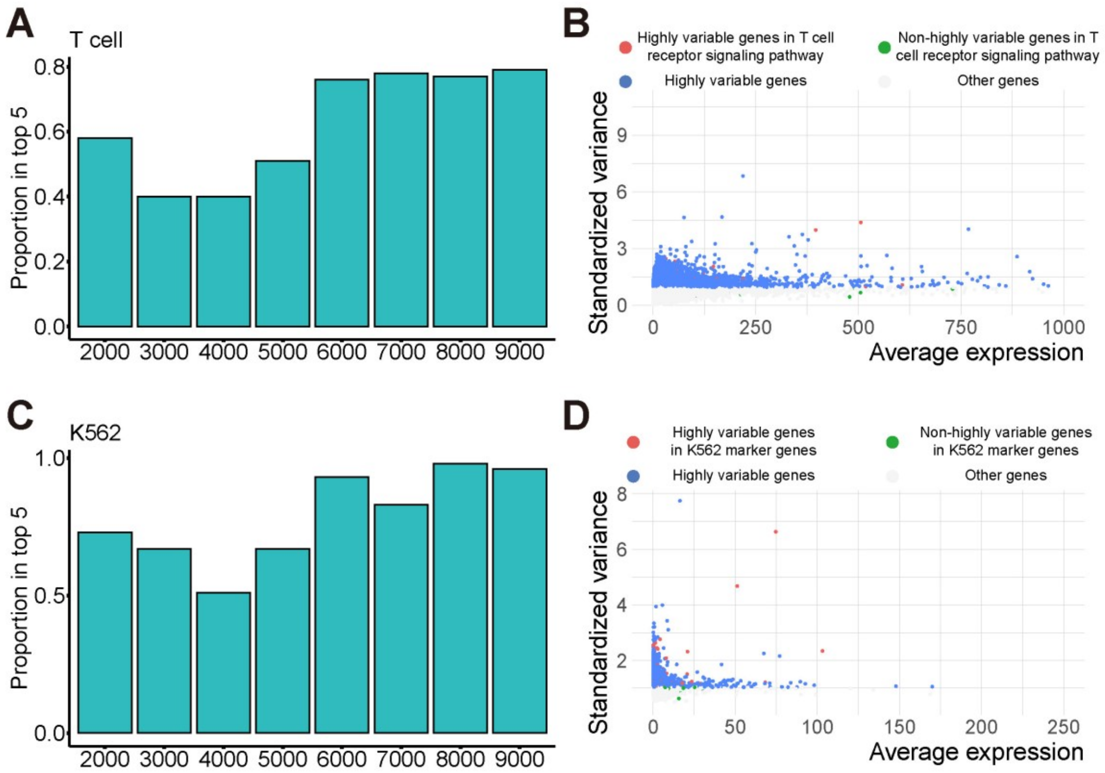

S33 Fig. Effect of increasing the number of genes in homogeneous data on the performance of scapGNN. Genes, initially 2000 highly variable genes and then increasing in count, were used to calculate individual cell pathway activity scores. (**A**) Proportion of T cells with the corresponding T-cell receptor signaling pathway in the top five for different gene counts. (**B**) Scatter plots show the relationship between the genes in the T-cell receptor signaling pathway and the 8000 highly variant genes that were screened. (**C**) Proportion of K562 cells with the corresponding marker gene set in the top five for different gene counts. (**D**) Scatter plots showing the relationship between the genes in the marker gene set of the K562 cells and the 8000 highly variant genes that were screened. The data underlying this figure can be found in S8 Data.
