# Supplementary material for: scapGNN: A graph neural network–based framework for active pathway and gene module inference from single-cell multi-omics data
Source: PLoS Biol. 2023 Nov 13;21(11):e3002369. doi: 10.1371/journal.pbio.3002369 (PMC10681325; doi:10.1371/journal.pbio.3002369)
Supplement: S34 Fig — Cell clustering indicators based on pathway activity scores and the proportion of endothelial cells with the corresponding marker gene set in the top 5 at different learning rates for DNNAE (A), number of iterations for DNNAE (B), learning rate for GAE (C), number of iterations for GAE (D), restart probability values (E), and number of perturbations (F). The data underlying this figure can be found in S8 Data. (PDF) [file pbio.3002369.s035.pdf]

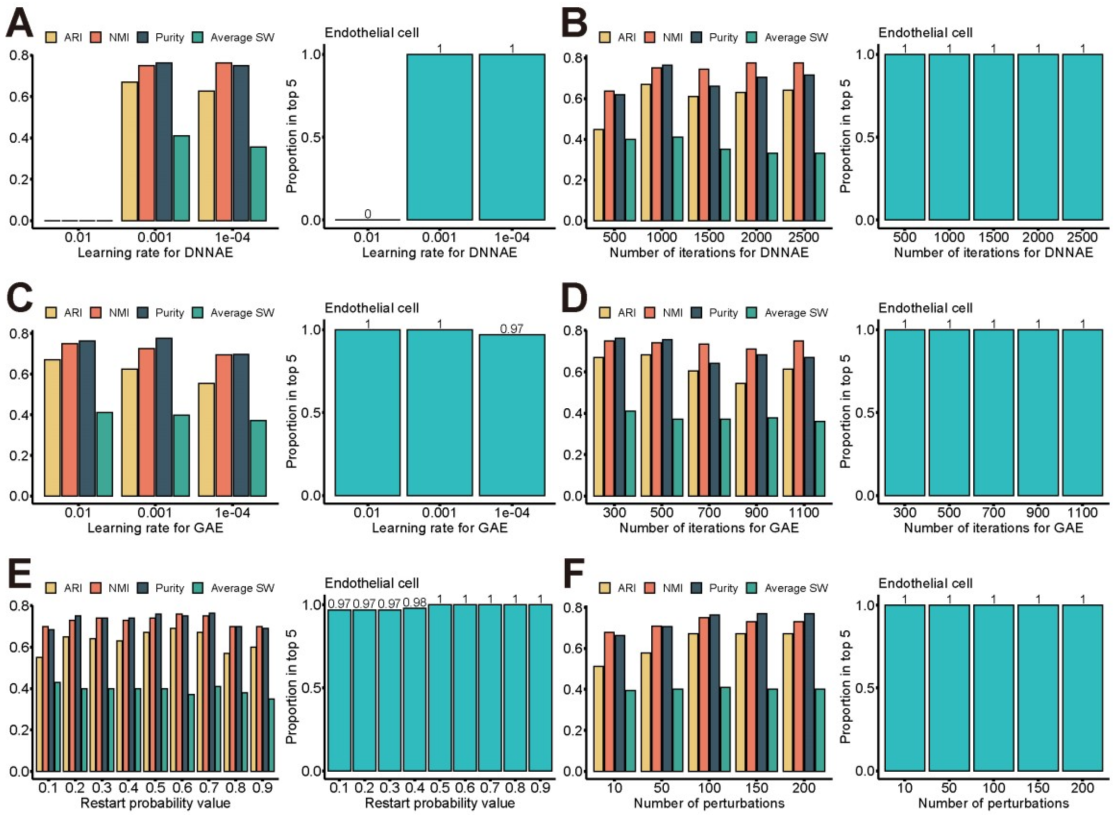

S34 Fig. Hyperparameter evaluation of the scapGNN using the cell type dataset. Cell clustering indicators based on pathway activity scores and the proportion of endothelial cells with the corresponding marker gene set in the top five at different learning rates for DNNAE (**A**), number of iterations for DNNAE (**B**), learning rate for GAE (**C**), number of iterations for GAE (**D**), restart probability values (**E**), and number of perturbations (**F**). The data underlying this figure can be found in S8 Data.
