# Supplementary material for: scapGNN: A graph neural network–based framework for active pathway and gene module inference from single-cell multi-omics data
Source: PLoS Biol. 2023 Nov 13;21(11):e3002369. doi: 10.1371/journal.pbio.3002369 (PMC10681325; doi:10.1371/journal.pbio.3002369)
Supplement: S35 Fig — C, adjacency matrix of the cell–cell association network; K, gene–cell association matrix; G, adjacency matrix for the gene–gene association network. (PDF) [file pbio.3002369.s036.pdf]

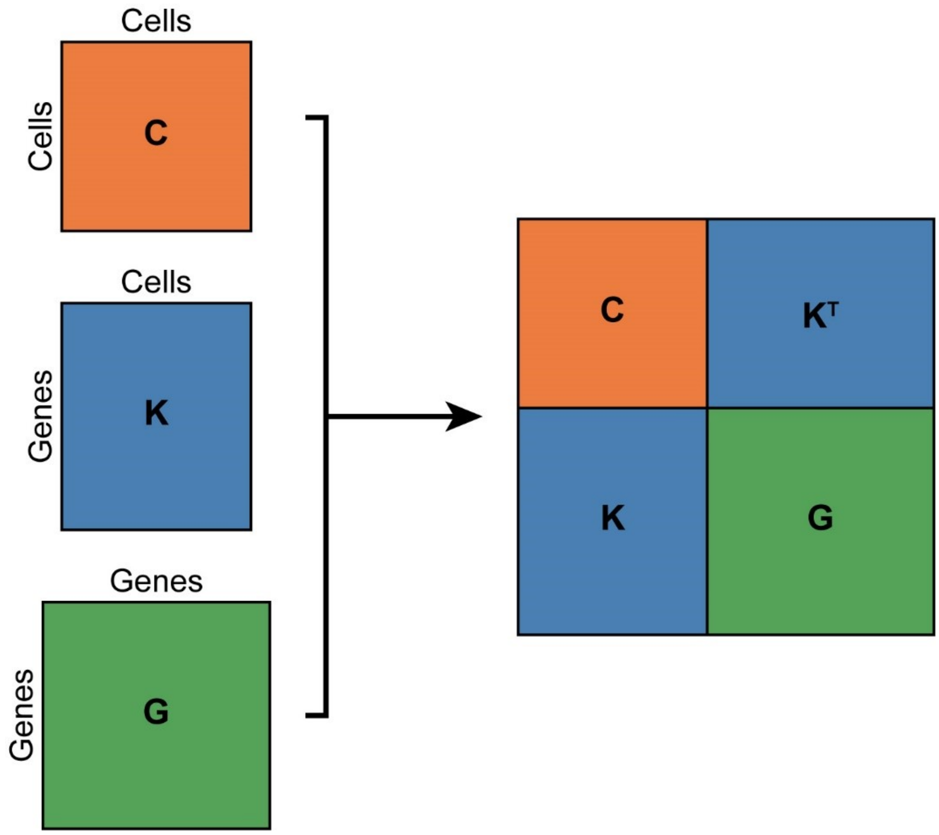

S35 Fig. Flowchart of constructing gene–cell association networks by integrating adjacency matrices. C, adjacency matrix of the cell–cell association network; K, gene–cell association matrix; G, adjacency matrix for the gene–gene association network.
