# Supplementary material for: scapGNN: A graph neural network–based framework for active pathway and gene module inference from single-cell multi-omics data
Source: PLoS Biol. 2023 Nov 13;21(11):e3002369. doi: 10.1371/journal.pbio.3002369 (PMC10681325; doi:10.1371/journal.pbio.3002369)
Supplement: S36 Fig — (A) Number of genes contained in marker gene sets. (B) Number of genes contained in marker gene sets for the cell types used in evaluation analysis. The data underlying this figure can be found in S8 Data. (PDF) [file pbio.3002369.s037.pdf]

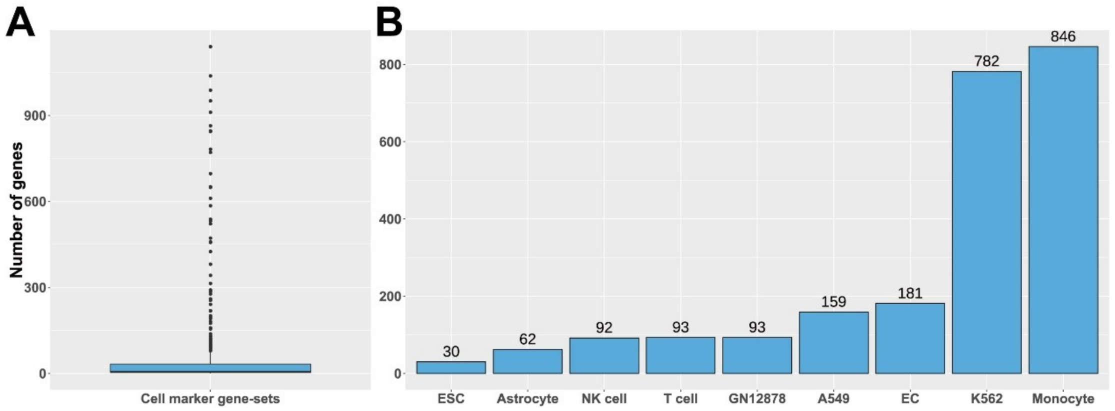

S36 Fig. Distribution of the number of genes in the cell type marker gene set. (**A**) Number of genes contained in marker gene sets. (**B**) Number of genes contained in marker gene sets for the cell types used in evaluation analysis. The data underlying this figure can be found in S8 Data.
