# Supplementary material for: scapGNN: A graph neural network–based framework for active pathway and gene module inference from single-cell multi-omics data
Source: PLoS Biol. 2023 Nov 13;21(11):e3002369. doi: 10.1371/journal.pbio.3002369 (PMC10681325; doi:10.1371/journal.pbio.3002369)
Supplement: S1 Table — (DOCX) [file pbio.3002369.s038.docx]

**S1 Table.** scRNA-seq datasets for the scapGNN application.

| **Accession** | **Protocol** | **Dataset** | **Homogeneous dataset** | **Number of Cells** |
| --- | --- | --- | --- | --- |
| GSE84133 [1] | inDrop | Cell type dataset | No | 1886 |
| GSE76381 [2] | 10x Genomics | Cell subtype dataset | No | 1715 |
| GSE75748 [2] | SMARTer | Time series dataset | No | 758 |
| GSE118767 [3] | Multiple protocols | Batch effects dataset | No | 4440 |
| GSE65525 [4] | CEL-Seq2 | K562 dataset | Yes | 239 |
| GSE118773 [5] | 10x Genomics | A549 dataset | Yes | 5254 |
| GSE81861 [6] | SMARTer | GM12878 dataset | No | 561 |
| GSE36552 [7] | Tang et.al. | ESC dataset | No | 124 |
| GSE81861 [6] | SMARTer | T cell and B cell dataset | No | 561 |
| GSE98638 [8] | Smart-seq2 | T cell dataset | Yes | 273 |
| Zheng et. al [9] | 10x Genomics | B cell dataset | Yes | 128 |
| GSE75748 [10] | SMARTer | EC dataset | No | 1018 |
| GSE108097 [11] | Microwell-seq | Mouse cell atlas dataset | No | 61637 |
| GSE126074 [12] | SNARE-seq | Mouse brain cortex dataset (scRNA-seq) | No | 10309 |
| GSE107644 [13] | Smart-seq2 | Mouse spermatogenesis dataset | No | 1203 |
| GSE136714[14] | scNOMeRe-seq | Mouse early embryo development dataset | No | 401 |
| GSE149512 [15] | 10x Genomics | Human testis dataset | No | 3225 |
| CNP0001102 [16] | DNBelab C4 | COVID-19 dataset | No | 5929 |

**References**

1. Baron M, Veres A, Wolock SL, Faust AL, Gaujoux R, Vetere A, et al. A Single-Cell Transcriptomic Map of the Human and Mouse Pancreas Reveals Inter- and Intra-cell Population Structure. Cell systems. 2016;3(4):346-60.e4. Epub 2016/10/28. doi: 10.1016/j.cels.2016.08.011. PubMed PMID: 27667365; PubMed Central PMCID: PMCPMC5228327.

2. La Manno G, Gyllborg D, Codeluppi S, Nishimura K, Salto C, Zeisel A, et al. Molecular Diversity of Midbrain Development in Mouse, Human, and Stem Cells. Cell. 2016;167(2):566-80.e19. Epub 2016/10/08. doi: 10.1016/j.cell.2016.09.027. PubMed PMID: 27716510; PubMed Central PMCID: PMCPMC5055122.

3. Tian L, Dong X, Freytag S, KA LC, Su S, JalalAbadi A, et al. Benchmarking single cell RNA-sequencing analysis pipelines using mixture control experiments. Nat Methods. 2019;16(6):479-87. Epub 2019/05/28. doi: 10.1038/s41592-019-0425-8. PubMed PMID: 31133762.

4. Klein AM, Mazutis L, Akartuna I, Tallapragada N, Veres A, Li V, et al. Droplet barcoding for single-cell transcriptomics applied to embryonic stem cells. Cell. 2015;161(5):1187-201. Epub 2015/05/23. doi: 10.1016/j.cell.2015.04.044. PubMed PMID: 26000487; PubMed Central PMCID: PMCPMC4441768.

5. Wang C, Forst CV, Chou TW, Geber A, Wang M, Hamou W, et al. Cell-to-Cell Variation in Defective Virus Expression and Effects on Host Responses during Influenza Virus Infection. mBio. 2020;11(1). Epub 2020/01/16. doi: 10.1128/mBio.02880-19. PubMed PMID: 31937643; PubMed Central PMCID: PMCPMC6960286.

6. Li H, Courtois ET, Sengupta D, Tan Y, Chen KH, Goh JJL, et al. Reference component analysis of single-cell transcriptomes elucidates cellular heterogeneity in human colorectal tumors. Nature genetics. 2017;49(5):708-18. Epub 2017/03/21. doi: 10.1038/ng.3818. PubMed PMID: 28319088.

7. Yan L, Yang M, Guo H, Yang L, Wu J, Li R, et al. Single-cell RNA-Seq profiling of human preimplantation embryos and embryonic stem cells. Nature structural & molecular biology. 2013;20(9):1131-9. Epub 2013/08/13. doi: 10.1038/nsmb.2660. PubMed PMID: 23934149.

8. Zheng C, Zheng L, Yoo JK, Guo H, Zhang Y, Guo X, et al. Landscape of Infiltrating T Cells in Liver Cancer Revealed by Single-Cell Sequencing. Cell. 2017;169(7):1342-56.e16. Epub 2017/06/18. doi: 10.1016/j.cell.2017.05.035. PubMed PMID: 28622514.

9. Zheng GXY, Terry JM, Belgrader P, Ryvkin P, Bent ZW, Wilson R, et al. Massively parallel digital transcriptional profiling of single cells. Nature Communications. 2017;8(1):14049. doi: 10.1038/ncomms14049.

10. Chu LF, Leng N, Zhang J, Hou Z, Mamott D, Vereide DT, et al. Single-cell RNA-seq reveals novel regulators of human embryonic stem cell differentiation to definitive endoderm. Genome biology. 2016;17(1):173. Epub 2016/08/19. doi: 10.1186/s13059-016-1033-x. PubMed PMID: 27534536; PubMed Central PMCID: PMCPMC4989499.

11. Han X, Wang R, Zhou Y, Fei L, Sun H, Lai S, et al. Mapping the Mouse Cell Atlas by Microwell-Seq. Cell. 2018;172(5):1091-107.e17. Epub 2018/02/24. doi: 10.1016/j.cell.2018.02.001. PubMed PMID: 29474909.

12. Chen S, Lake BB, Zhang K. High-throughput sequencing of the transcriptome and chromatin accessibility in the same cell. Nat Biotechnol. 2019;37(12):1452-7. Epub 2019/10/16. doi: 10.1038/s41587-019-0290-0. PubMed PMID: 31611697; PubMed Central PMCID: PMCPMC6893138.

13. Chen Y, Zheng Y, Gao Y, Lin Z, Yang S, Wang T, et al. Single-cell RNA-seq uncovers dynamic processes and critical regulators in mouse spermatogenesis. Cell Res. 2018;28(9):879-96. Epub 2018/08/01. doi: 10.1038/s41422-018-0074-y. PubMed PMID: 30061742; PubMed Central PMCID: PMCPMC6123400.

14. Wang Y, Yuan P, Yan Z, Yang M, Huo Y, Nie Y, et al. Single-cell multiomics sequencing reveals the functional regulatory landscape of early embryos. Nat Commun. 2021;12(1):1247. Epub 2021/02/25. doi: 10.1038/s41467-021-21409-8. PubMed PMID: 33623021; PubMed Central PMCID: PMCPMC7902657.

15. Zhao L, Yao C, Xing X, Jing T, Li P, Zhu Z, et al. Single-cell analysis of developing and azoospermia human testicles reveals central role of Sertoli cells. Nat Commun. 2020;11(1):5683. Epub 2020/11/12. doi: 10.1038/s41467-020-19414-4. PubMed PMID: 33173058; PubMed Central PMCID: PMCPMC7655944.

16. Zhu L, Yang P, Zhao Y, Zhuang Z, Wang Z, Song R, et al. Single-Cell Sequencing of Peripheral Mononuclear Cells Reveals Distinct Immune Response Landscapes of COVID-19 and Influenza Patients. Immunity. 2020;53(3):685-96.e3. Epub 2020/08/14. doi: 10.1016/j.immuni.2020.07.009. PubMed PMID: 32783921; PubMed Central PMCID: PMCPMC7368915.
