# Supplementary material for: scapGNN: A graph neural network–based framework for active pathway and gene module inference from single-cell multi-omics data
Source: PLoS Biol. 2023 Nov 13;21(11):e3002369. doi: 10.1371/journal.pbio.3002369 (PMC10681325; doi:10.1371/journal.pbio.3002369)
Supplement: S2 Table — (DOCX) [file pbio.3002369.s039.docx]

**S2 Table.** State-of-the-art methods for the comparison of performance and manuals.

| **Method** | **Manual** |
| --- | --- |
| GSVA [1] | https://www.bioconductor.org/packages/release/bioc/html/GSVA.html |
| ssGSEA [2] | https://www.bioconductor.org/packages/release/bioc/html/GSVA.html |
| AUCell [3] | http://sctpa.bio-data.cn/sctpa/ |
| Pagoda2 [4] | http://sctpa.bio-data.cn/sctpa/ |
| UniPath [5] | https://reggenlab.github.io/UniPathWeb/ |
| genomap [6] | https://github.com/xinglab-ai/genomap |
| UINMF [7] | http://htmlpreview.github.io/?https://github.com/welch-lab/liger/blob/master/vignettes/SNAREseq_walkthrough.html |
| MOFA2 [8] | https://raw.githack.com/bioFAM/MOFA2_tutorials/master/R_tutorials/10x_scRNA_scATAC.html |
| Seurat v4 [9] | https://satijalab.org/seurat/articles/weighted_nearest_neighbor_analysis.html |
| Cobolt [10] | https://github.com/epurdom/cobolt/blob/master/docs/tutorial.ipynb |
| GLUE [11] | https://scglue.readthedocs.io/zh_CN/latest/tutorials.html |
| scDART [12] | https://github.com/PeterZZQ/scDART/blob/master/Examples/demo.ipynb |

**References**

1. Hänzelmann S, Castelo R, Guinney J. GSVA: gene set variation analysis for microarray and RNA-seq data. BMC bioinformatics. 2013;14:7. Epub 2013/01/18. doi: 10.1186/1471-2105-14-7. PubMed PMID: 23323831; PubMed Central PMCID: PMCPMC3618321.

2. Lee E, Chuang H-Y, Kim J-W, Ideker T, Lee D. Inferring Pathway Activity toward Precise Disease Classification. PLoS computational biology. 2008;4(11):e1000217. doi: 10.1371/journal.pcbi.1000217.

3. Aibar S, González-Blas CB, Moerman T, Huynh-Thu VA, Imrichova H, Hulselmans G, et al. SCENIC: single-cell regulatory network inference and clustering. Nat Methods. 2017;14(11):1083-6. Epub 2017/10/11. doi: 10.1038/nmeth.4463. PubMed PMID: 28991892; PubMed Central PMCID: PMCPMC5937676.

4. Lake BB, Chen S, Sos BC, Fan J, Kaeser GE, Yung YC, et al. Integrative single-cell analysis of transcriptional and epigenetic states in the human adult brain. Nat Biotechnol. 2018;36(1):70-80. Epub 2017/12/12. doi: 10.1038/nbt.4038. PubMed PMID: 29227469; PubMed Central PMCID: PMCPMC5951394.

5. Chawla S, Samydurai S, Kong SL, Wu Z, Wang Z, Tam WL, et al. UniPath: a uniform approach for pathway and gene-set based analysis of heterogeneity in single-cell epigenome and transcriptome profiles. Nucleic Acids Res. 2021;49(3):e13. Epub 2020/12/05. doi: 10.1093/nar/gkaa1138. PubMed PMID: 33275158; PubMed Central PMCID: PMCPMC7897496.

6. Islam MT, Xing L. Cartography of Genomic Interactions Enables Deep Analysis of Single-Cell Expression Data. Nature Communications. 2023;14(1):679. doi: 10.1038/s41467-023-36383-6.

7. Kriebel AR, Welch JD. UINMF performs mosaic integration of single-cell multi-omic datasets using nonnegative matrix factorization. Nature Communications. 2022;13(1):780. doi: 10.1038/s41467-022-28431-4.

8. Argelaguet R, Arnol D, Bredikhin D, Deloro Y, Velten B, Marioni JC, et al. MOFA+: a statistical framework for comprehensive integration of multi-modal single-cell data. Genome biology. 2020;21(1):111. doi: 10.1186/s13059-020-02015-1.

9. Hao Y, Hao S, Andersen-Nissen E, Mauck WM, Zheng S, Butler A, et al. Integrated analysis of multimodal single-cell data. Cell. 2021;184(13):3573-87.e29. doi: <https://doi.org/10.1016/j.cell.2021.04.048>.

10. Gong B, Zhou Y, Purdom E. Cobolt: integrative analysis of multimodal single-cell sequencing data. Genome biology. 2021;22(1):351. Epub 2021/12/30. doi: 10.1186/s13059-021-02556-z. PubMed PMID: 34963480; PubMed Central PMCID: PMCPMC8715620.

11. Cao ZJ, Gao G. Multi-omics single-cell data integration and regulatory inference with graph-linked embedding. Nat Biotechnol. 2022;40(10):1458-66. Epub 2022/05/03. doi: 10.1038/s41587-022-01284-4. PubMed PMID: 35501393; PubMed Central PMCID: PMCPMC9546775.

12. Zhang Z, Yang C, Zhang X. scDART: integrating unmatched scRNA-seq and scATAC-seq data and learning cross-modality relationship simultaneously. Genome biology. 2022;23(1):139. doi: 10.1186/s13059-022-02706-x.
