# Supplementary material for: scapGNN: A graph neural network–based framework for active pathway and gene module inference from single-cell multi-omics data
Source: PLoS Biol. 2023 Nov 13;21(11):e3002369. doi: 10.1371/journal.pbio.3002369 (PMC10681325; doi:10.1371/journal.pbio.3002369)
Supplement: S3 Table — (DOCX) [file pbio.3002369.s040.docx]

**S3 Table.** Benchmark datasets for performance evaluation of cell clustering.

| **Accession** | **Protocol** | **Species** | **Organ** | **Zero-valued rate** | **Average gene number** | **Number of Cells** | **UMI** |
| --- | --- | --- | --- | --- | --- | --- | --- |
| GSE109999 [1] | CEL-Seq2 | Mouse | Blood | 0.868 | 1951.4 | 383 | Yes |
| GSE171907 [2] | 10x Genomics | Mouse | Lung | 0.932 | 1254.3 | 2991 | Yes |
| GSE183320 [3] | 10x Genomics | Human | Choroids | 0.835 | 2298.8 | 1494 | Yes |
| GSE67835 [4] | SMARTer | Human | Brain | 0.767 | 4230.1 | 466 | No |
| GSE75688 [5] | Fluidigm C1 | Human | Breast and lymph node | 0.892 | 6039.8 | 527 | No |
| GSE75748 (cell lines) [6] | SMARTer | Human | Stem cell | 0.496 | 9616.9 | 1018 | No |
| GSE75748 (timeseries) [6] | SMARTer | Human | Stem cell | 0.547 | 8687.1 | 758 | No |
| GSE76381 (ES) [7] | 10x Genomics | Human | Midbrain | 0.766 | 3625.5 | 1715 | Yes |
| GSE76381 (MouseEm) [7] | 10x Genomics | Mouse | Midbrain | 0.791 | 2920.7 | 1907 | Yes |
| GSE81252 [8] | SMARTer | Human | Liver | 0.611 | 5820.2 | 465 | No |
| GSE81608 [9] | SMARTer | Human | Pancreas | 0.856 | 5693.3 | 1600 | No |
| GSE84133 [10] | inDrop | Mouse | Pancreas | 0.863 | 1627.3 | 1886 | Yes |
| GSE87375 [11] | Smart-seq2 | Mouse | Pancreas | 0.719 | 7758.6 | 917 | No |
| GSE52583 [12] | Fluidigm C1 | Mouse | Lung | 0.743 | 3604.6 | 201 | No |
| E-MTAB-5061 [13] | Smart-seq2 | Human | Pancreas | 0.718 | 4733.1 | 3514 | No |
| GSE74672 [14] | Fluidigm C1 | Mouse | Hypothalamus | 0.793 | 2960.1 | 2881 | No |

**References**

1. Tian L, Su S, Dong X, Amann-Zalcenstein D, Biben C, Seidi A, et al. scPipe: A flexible R/Bioconductor preprocessing pipeline for single-cell RNA-sequencing data. PLoS computational biology. 2018;14(8):e1006361. Epub 2018/08/11. doi: 10.1371/journal.pcbi.1006361. PubMed PMID: 30096152; PubMed Central PMCID: PMCPMC6105007.

2. Xu J, Xu L, Sui P, Chen J, Moya EA, Hume P, et al. Excess neuropeptides in lung signal through endothelial cells to impair gas exchange. Developmental cell. 2022;57(7):839-53.e6. Epub 2022/03/19. doi: 10.1016/j.devcel.2022.02.023. PubMed PMID: 35303432.

3. Choroidal Endothelial and Macrophage Gene Expression in Atrophic and Neovascular Macular Degeneration [Internet]. 2022 [cited Feb 19].

4. Darmanis S, Sloan SA, Zhang Y, Enge M, Caneda C, Shuer LM, et al. A survey of human brain transcriptome diversity at the single cell level. Proceedings of the National Academy of Sciences of the United States of America. 2015;112(23):7285-90. Epub 2015/06/11. doi: 10.1073/pnas.1507125112. PubMed PMID: 26060301; PubMed Central PMCID: PMCPMC4466750.

5. Chung W, Eum HH, Lee HO, Lee KM, Lee HB, Kim KT, et al. Single-cell RNA-seq enables comprehensive tumour and immune cell profiling in primary breast cancer. Nat Commun. 2017;8:15081. Epub 2017/05/06. doi: 10.1038/ncomms15081. PubMed PMID: 28474673; PubMed Central PMCID: PMCPMC5424158.

6. Chu LF, Leng N, Zhang J, Hou Z, Mamott D, Vereide DT, et al. Single-cell RNA-seq reveals novel regulators of human embryonic stem cell differentiation to definitive endoderm. Genome biology. 2016;17(1):173. Epub 2016/08/19. doi: 10.1186/s13059-016-1033-x. PubMed PMID: 27534536; PubMed Central PMCID: PMCPMC4989499.

7. La Manno G, Gyllborg D, Codeluppi S, Nishimura K, Salto C, Zeisel A, et al. Molecular Diversity of Midbrain Development in Mouse, Human, and Stem Cells. Cell. 2016;167(2):566-80.e19. Epub 2016/10/08. doi: 10.1016/j.cell.2016.09.027. PubMed PMID: 27716510; PubMed Central PMCID: PMCPMC5055122.

8. Differences and similarities between human and chimpanzee neural progenitors during cerebral cortex development [Internet]. 2016 [cited Sep 26].

9. Xin Y, Kim J, Okamoto H, Ni M, Wei Y, Adler C, et al. RNA Sequencing of Single Human Islet Cells Reveals Type 2 Diabetes Genes. Cell metabolism. 2016;24(4):608-15. Epub 2016/09/27. doi: 10.1016/j.cmet.2016.08.018. PubMed PMID: 27667665.

10. Baron M, Veres A, Wolock SL, Faust AL, Gaujoux R, Vetere A, et al. A Single-Cell Transcriptomic Map of the Human and Mouse Pancreas Reveals Inter- and Intra-cell Population Structure. Cell systems. 2016;3(4):346-60.e4. Epub 2016/10/28. doi: 10.1016/j.cels.2016.08.011. PubMed PMID: 27667365; PubMed Central PMCID: PMCPMC5228327.

11. Qiu WL, Zhang YW, Feng Y, Li LC, Yang L, Xu CR. Deciphering Pancreatic Islet β Cell and α Cell Maturation Pathways and Characteristic Features at the Single-Cell Level. Cell metabolism. 2017;25(5):1194-205.e4. Epub 2017/05/04. doi: 10.1016/j.cmet.2017.04.003. PubMed PMID: 28467935.

12. Treutlein B, Brownfield DG, Wu AR, Neff NF, Mantalas GL, Espinoza FH, et al. Reconstructing lineage hierarchies of the distal lung epithelium using single-cell RNA-seq. Nature. 2014;509(7500):371-5. Epub 2014/04/18. doi: 10.1038/nature13173. PubMed PMID: 24739965; PubMed Central PMCID: PMCPMC4145853.

13. Segerstolpe Å, Palasantza A, Eliasson P, Andersson EM, Andréasson AC, Sun X, et al. Single-Cell Transcriptome Profiling of Human Pancreatic Islets in Health and Type 2 Diabetes. Cell metabolism. 2016;24(4):593-607. Epub 2016/09/27. doi: 10.1016/j.cmet.2016.08.020. PubMed PMID: 27667667; PubMed Central PMCID: PMCPMC5069352.

14. Romanov RA, Zeisel A, Bakker J, Girach F, Hellysaz A, Tomer R, et al. Molecular interrogation of hypothalamic organization reveals distinct dopamine neuronal subtypes. Nature neuroscience. 2017;20(2):176-88. Epub 2016/12/20. doi: 10.1038/nn.4462. PubMed PMID: 27991900.
