# Supplementary material for: scapGNN: A graph neural network–based framework for active pathway and gene module inference from single-cell multi-omics data
Source: PLoS Biol. 2023 Nov 13;21(11):e3002369. doi: 10.1371/journal.pbio.3002369 (PMC10681325; doi:10.1371/journal.pbio.3002369)
Supplement: S4 Table — (DOCX) [file pbio.3002369.s041.docx]

**S4 Table.** Single-cell clustering methods.

| **Method** | **Tool** | **Version** | **Available** |
| --- | --- | --- | --- |
| K-means | stats | 3.5.2 | <https://stat.ethz.ch/R-manual/R-devel/library/stats/html/stats-package.html> |
| Hierarchical clustering | fastcluster | 1.1.25 | <https://cran.r-project.org/web/packages/fastcluster/index.html> |
| Spectral clustering | SNFtool | 2.3.0 | <https://cran.r-project.org/web/packages/SNFtool/index.html> |
| DBSCAN | dbscan | 1.13 | <https://cran.r-project.org/web/packages/dbscan/index.html> |
| Seurat | Seurat | 4 | <https://cran.r-project.org/web/packages/Seurat/index.html> |
| SOUP | SOUP | 0.0.0.9 | https://github.com/lingxuez/SOUPR |
| CIDR | cidr | 0.1.5 | <https://github.com/VCCRI/CIDR> |
| pcaReduce | pcaReduce | 1.0 | <https://github.com/JustinaZ/pcaReduce> |
| SNN-Cliq | SNN-cliq | 0.0.0 | http://bioinfo.uncc.edu/SNNCliq |
| SC3 | SC3 | 1.10.1 | <http://bioconductor.org/packages/sc3> |
