# Supplementary material for: scapGNN: A graph neural network–based framework for active pathway and gene module inference from single-cell multi-omics data
Source: PLoS Biol. 2023 Nov 13;21(11):e3002369. doi: 10.1371/journal.pbio.3002369 (PMC10681325; doi:10.1371/journal.pbio.3002369)
Supplement: S5 Table — (DOCX) [file pbio.3002369.s042.docx]

**S5 Table.** Omics datasets for evaluation of pathway and gene module identification.

| **Accession** | **Protocol** | **Dataset** | **Homogeneous dataset** | **Number of Cells** |
| --- | --- | --- | --- | --- |
| [10x Genomics](https://www.10xgenomics.com/resources/datasets/fresh-cortex-from-adult-mouse-brain-p-50-1-standard-1-1-0) | scATAC-seq | Mouse cortical brain dataset | No | 5000 |
| [10x Genomics](https://support.10xgenomics.com/single-cell-multiome-atac-gex/datasets) | scATAC-seq | PBMC dataset | No | 11909 |
| GSE126074 [1] | SNARE-seq | Mouse brain cortex dataset (multi-omics) | No | 10309 |
| GSE140203 [2] | SHARE-seq | Mouse skin dataset (multi-omics) | No | 17411 |
| GSE140203 [2] | SHARE-seq | GM12878 dataset (multi-omics) | Yes | 1215 |
| [10x Genomics](https://support.10xgenomics.com/single-cell-multiome-atac-gex/datasets) | 10x multiome | PBMC multi-omics dataset | No | 11909 |

**References**

1. Chen S, Lake BB, Zhang K. High-throughput sequencing of the transcriptome and chromatin accessibility in the same cell. Nat Biotechnol. 2019;37(12):1452-7. Epub 2019/10/16. doi: 10.1038/s41587-019-0290-0. PubMed PMID: 31611697; PubMed Central PMCID: PMCPMC6893138.

2. Ma S, Zhang B, LaFave LM, Earl AS, Chiang Z, Hu Y, et al. Chromatin Potential Identified by Shared Single-Cell Profiling of RNA and Chromatin. Cell. 2020;183(4):1103-16.e20. doi: <https://doi.org/10.1016/j.cell.2020.09.056>.
