# Supplementary material for: scapGNN: A graph neural network–based framework for active pathway and gene module inference from single-cell multi-omics data
Source: PLoS Biol. 2023 Nov 13;21(11):e3002369. doi: 10.1371/journal.pbio.3002369 (PMC10681325; doi:10.1371/journal.pbio.3002369)
Supplement: S6 Table — (DOCX) [file pbio.3002369.s043.docx]

**S6 Table.** Pathways with significantly different in certain types of cells only in integrated multi-omics data of scRNA-seq and scATAC-seq from adult mouse brain.

| **Pathway** | **Cell type** | **Adjusted p-value (scapGNN, multi-omics)** | | **Reference** |
| --- | --- | --- | --- | --- |
| Glucagon signaling pathway | Astrocyte | | 2.29e-91 | [1, 2] |
| Linoleic acid metabolism | Astrocyte | | 3.27e-89 | [3, 4] |
| Lysine degradation | Astrocyte | | 1.39e-39 | [5] |
| Pattern recognition receptors | Astrocyte | | 1.11e-22 | [6] |
| Regulation of actin cytoskeleton | Astrocyte | | 1.33e-10 | [7] |
| ErbB signaling pathway | Astrocyte | | 2.48e-08 | [8, 9] |
| AMPK signaling pathway | Oligodendrocyte | | 1.21e-59 | [10] |
| Fatty acid degradation | Oligodendrocyte | | 1.65e-12 | [11, 12] |
| Arginine and proline metabolism | Oligodendrocyte | | 2.01e-11 | [13] |
| Notch signaling pathway | Oligodendrocyte | | 5.32e-08 | [14, 15] |

**References**

1. Timper K, Del Río-Martín A, Cremer AL, Bremser S, Alber J, Giavalisco P, et al. GLP-1 Receptor Signaling in Astrocytes Regulates Fatty Acid Oxidation, Mitochondrial Integrity, and Function. Cell metabolism. 2020;31(6):1189-205.e13. Epub 2020/05/21. doi: 10.1016/j.cmet.2020.05.001. PubMed PMID: 32433922; PubMed Central PMCID: PMCPMC7272126.

2. Shan Y, Tan S, Lin Y, Liao S, Zhang B, Chen X, et al. The glucagon-like peptide-1 receptor agonist reduces inflammation and blood-brain barrier breakdown in an astrocyte-dependent manner in experimental stroke. Journal of neuroinflammation. 2019;16(1):242. Epub 2019/11/30. doi: 10.1186/s12974-019-1638-6. PubMed PMID: 31779652; PubMed Central PMCID: PMCPMC6883580.

3. Turovsky EA, Varlamova EG, Gudkov SV, Plotnikov EY. The Protective Mechanism of Deuterated Linoleic Acid Involves the Activation of the Ca(2+) Signaling System of Astrocytes in Ischemia In Vitro. International journal of molecular sciences. 2021;22(24). Epub 2021/12/25. doi: 10.3390/ijms222413216. PubMed PMID: 34948013; PubMed Central PMCID: PMCPMC8706680.

4. Murphy MG. Effects of exogenous linoleic acid on fatty acid composition, receptor-mediated cAMP formation, and transport functions in rat astrocytes in primary culture. Neurochemical research. 1995;20(11):1365-75. Epub 1995/11/01. doi: 10.1007/bf00992513. PubMed PMID: 8786824.

5. Tapias A, Wang ZQ. Lysine Acetylation and Deacetylation in Brain Development and Neuropathies. Genomics, proteomics & bioinformatics. 2017;15(1):19-36. Epub 2017/02/06. doi: 10.1016/j.gpb.2016.09.002. PubMed PMID: 28161493; PubMed Central PMCID: PMCPMC5339409.

6. Li L, Acioglu C, Heary RF, Elkabes S. Role of astroglial toll-like receptors (TLRs) in central nervous system infections, injury and neurodegenerative diseases. Brain, behavior, and immunity. 2021;91:740-55. Epub 2020/10/12. doi: 10.1016/j.bbi.2020.10.007. PubMed PMID: 33039660; PubMed Central PMCID: PMCPMC7543714.

7. Sandsmark DK, Zhang H, Hegedus B, Pelletier CL, Weber JD, Gutmann DH. Nucleophosmin mediates mammalian target of rapamycin-dependent actin cytoskeleton dynamics and proliferation in neurofibromin-deficient astrocytes. Cancer research. 2007;67(10):4790-9. Epub 2007/05/19. doi: 10.1158/0008-5472.can-06-4470. PubMed PMID: 17510408.

8. Sharif A, Duhem-Tonnelle V, Allet C, Baroncini M, Loyens A, Kerr-Conte J, et al. Differential erbB signaling in astrocytes from the cerebral cortex and the hypothalamus of the human brain. Glia. 2009;57(4):362-79. Epub 2008/09/23. doi: 10.1002/glia.20762. PubMed PMID: 18803307.

9. Sharif A, Prevot V. ErbB receptor signaling in astrocytes: a mediator of neuron-glia communication in the mature central nervous system. Neurochemistry international. 2010;57(4):344-58. Epub 2010/08/06. doi: 10.1016/j.neuint.2010.05.012. PubMed PMID: 20685225.

10. Paintlia AS, Paintlia MK, Mohan S, Singh AK, Singh I. AMP-activated protein kinase signaling protects oligodendrocytes that restore central nervous system functions in an experimental autoimmune encephalomyelitis model. The American journal of pathology. 2013;183(2):526-41. Epub 2013/06/14. doi: 10.1016/j.ajpath.2013.04.030. PubMed PMID: 23759513; PubMed Central PMCID: PMCPMC3730772.

11. Cheng A, Jia W, Kawahata I, Fukunaga K. A novel fatty acid-binding protein 5 and 7 inhibitor ameliorates oligodendrocyte injury in multiple sclerosis mouse models. EBioMedicine. 2021;72:103582. Epub 2021/10/09. doi: 10.1016/j.ebiom.2021.103582. PubMed PMID: 34624687; PubMed Central PMCID: PMCPMC8502714.

12. Dimas P, Montani L, Pereira JA, Moreno D, Trötzmüller M, Gerber J, et al. CNS myelination and remyelination depend on fatty acid synthesis by oligodendrocytes. eLife. 2019;8. Epub 2019/05/08. doi: 10.7554/eLife.44702. PubMed PMID: 31063129; PubMed Central PMCID: PMCPMC6504237.

13. Dansu DK, Liang J, Selcen I, Zheng H, Moore DF, Casaccia P. PRMT5 Interacting Partners and Substrates in Oligodendrocyte Lineage Cells. Frontiers in cellular neuroscience. 2022;16:820226. Epub 2022/04/05. doi: 10.3389/fncel.2022.820226. PubMed PMID: 35370564; PubMed Central PMCID: PMCPMC8968030.

14. López-Juárez A, Titus HE, Silbak SH, Pressler JW, Rizvi TA, Bogard M, et al. Oligodendrocyte Nf1 Controls Aberrant Notch Activation and Regulates Myelin Structure and Behavior. Cell reports. 2017;19(3):545-57. Epub 2017/04/20. doi: 10.1016/j.celrep.2017.03.073. PubMed PMID: 28423318; PubMed Central PMCID: PMCPMC5828008.

15. Gómez Pinto LI, Rodríguez D, Adamo AM, Mathieu PA. TGF-β pro-oligodendrogenic effects on adult SVZ progenitor cultures and its interaction with the Notch signaling pathway. Glia. 2018;66(2):396-412. Epub 2017/10/28. doi: 10.1002/glia.23253. PubMed PMID: 29076551.
