# Supplementary material for: scapGNN: A graph neural network–based framework for active pathway and gene module inference from single-cell multi-omics data
Source: PLoS Biol. 2023 Nov 13;21(11):e3002369. doi: 10.1371/journal.pbio.3002369 (PMC10681325; doi:10.1371/journal.pbio.3002369)
Supplement: S29 Fig — (A) UMAP visualizations of the scapGNN-derived pathway activity matrix for healthy controls and patients with COVID-19. (B) Gene association network of thyroid hormone synthesis in patients with COVID-19. The node size reflects the importance of the genes, and the width of the edges between nodes indicates the strength of association between genes in the network. (C) Network of cell phenotype–associated gene modules in healthy controls, patients with COVID-19, and those with IAV. In the network, the sector area of a node indicates the strength of association between a gene and a cell phenotype, and the width of the edges between nodes indicates the strength of association between genes. The data underlying this figure can be found in S8 Data. COVID-19, coronavirus disease 2019; IAV, influenza A virus; UMAP, Uniform Manifold Approximation and Projection. (PDF) [file pbio.3002369.s030.pdf]

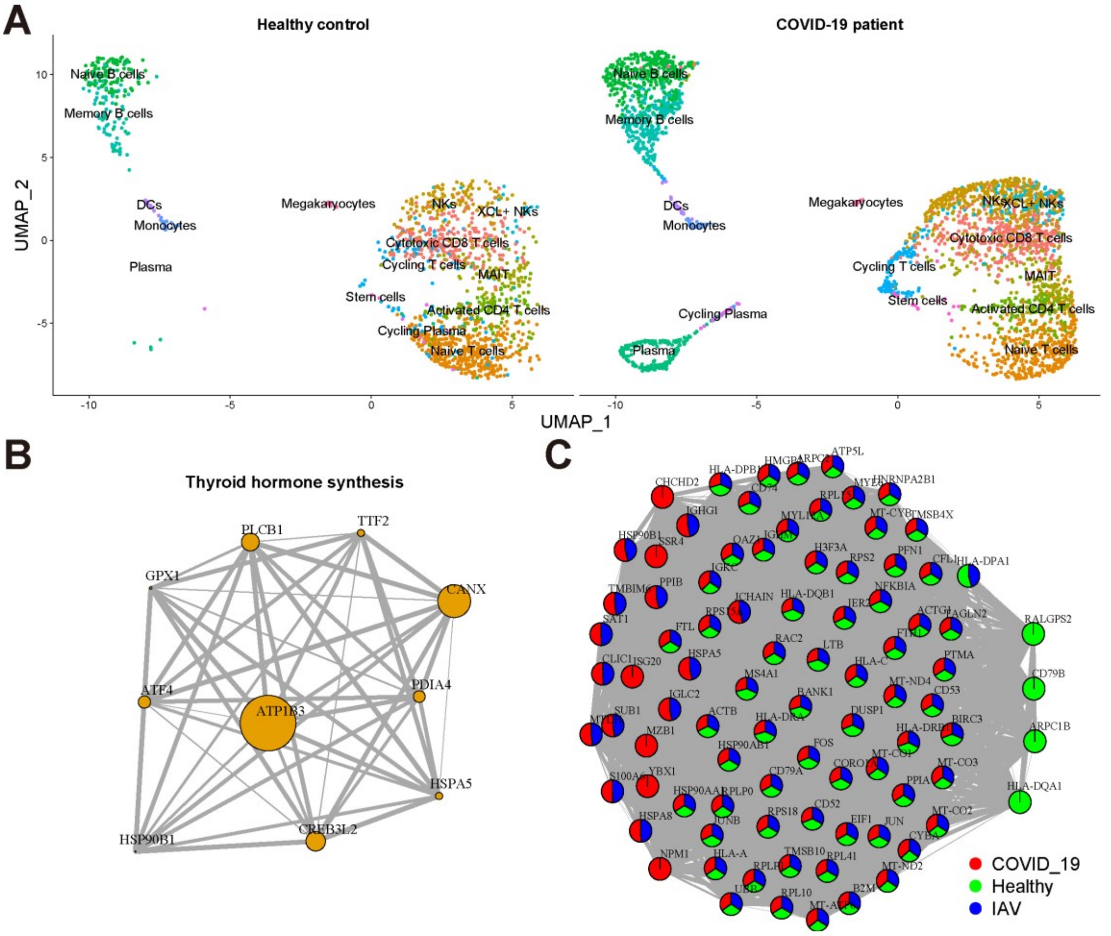

S29 Fig. Application of scapGNN to COVID-19 data. (**A**) UMAP visualizations of the scapGNN-derived pathway activity matrix for healthy controls and patients with COVID-19. (**B**) Gene association network of thyroid hormone synthesis in patients with COVID-19. The node size reflects the importance of the genes, and the width of the edges between nodes indicates the strength of association between genes in the network. (**C**) Network of cell phenotype–associated gene modules in healthy controls, patients with COVID-19, and those with IAV. In the network, the sector area of a node indicates the strength of association between a gene and a cell phenotype, and the width of the edges between nodes indicates the strength of association between genes. The data underlying this figure can be found in S8 Data.
