# Supplementary material for: scapGNN: A graph neural network–based framework for active pathway and gene module inference from single-cell multi-omics data
Source: PLoS Biol. 2023 Nov 13;21(11):e3002369. doi: 10.1371/journal.pbio.3002369 (PMC10681325; doi:10.1371/journal.pbio.3002369)
Supplement: Attachment — Submitted filename: Response.docx [file pbio.3002369.s052.docx]

Dear reviewers:

Thanks for the reviewers’ comments concerning our manuscript entitled “scapGNN: a graph neural network-based framework for active pathway and gene module inference from single-cell multi-omics data” (Manuscript ID: PBIOLOGY-D-23-00416R2). Those comments are all valuable and very helpful for revising and improving our paper, as well as the important guiding significance to our research. We have studied the comments carefully and have made corrections which we hope meet with approval. Revised portions are marked in RED in the paper. The main corrections in the paper and the response to the reviewer’s comments are as follows:

**Reply to Reviewer: 1**
*Reviewer #1: In this manuscript, the authors propose present scapGNN, a graph neural network (GNN)-based framework that calculates pathway activity scores, identifies cell phenotype-associated gene modules, and integrates multi-omics data at single-cell resolution. There exist a few scientific and presentation issues to be fixed.

Scientific issues:
1. Does selecting highly variable genes in data preprocessing eliminate some genes from the pathway and thus make the calculated activity score inaccurate, especially in homogeneous data?*

**REPLY:** Thanks for your comments, it makes a lot of sense to us. To assess the impact of highly variable gene selection on the performance of scapGNN and whether it leads to information loss, we performed benchmark experiments in which the number of highly variable genes was gradually increased. For heterogeneous single-cell data, cell clustering accuracy and marker gene set scoring decrease as the number of genes increases (S32 Fig). There are two main reasons for this: (i) not all genes contribute to cell-to-cell differences (DOI: 10.1093/bib/bby011); (ii) a major feature of single-cell data is the "dropout" which is due to the low amounts of mRNA in individual cells and inefficient mRNA capture, as well as the stochasticity of mRNA expression (DOI: 10.1038/s41467-020-14976-9). Too many genes can introduce technical noise. For homogeneous data, there is little variation in expression levels between cells for inherently expressed genes such as marker genes. A small number of highly variable genes can cause a loss of information (S33 Fig). Our results show that 8000 highly variable genes can represent information about intrinsic biological pathways or marker gene sets in cells while minimizing the introduction of noise (S33 Fig). Therefore, we applied the 8000 highly variable genes to the homogeneous data and updated the paper with the corresponding results. We have modified the following:

(1) Page 30, section "Data preprocessing" in the Methods.

"For heterogeneous data, not all genes in single-cell sequencing data contribute to cell-to-cell differences [84]. Due to dropout events in single-cell sequencing, too many genes can introduce technical noise (S32 Fig). We selected 2000 highly variable genes in processing heterogeneous single-cell data. However, for homogeneous data, intrinsically highly expressed genes, such as marker genes, tend to have smaller variances (S33 Fig). In order not to cause data loss, we selected 8000 highly variable genes in processing homogeneous single-cell data."

(2) Page 57, S32 Fig in the Supporting information.

(3) Page 57, S33 Fig in the Supporting information.

(4) Page 49, Fig 3 in the Manuscript.

*2. Since the application scenario is different from Kohler's study, please do experiments to show that different choices of r do not have a large impact on the result of the RWR algorithm.*

**REPLY:** Thank you for your suggestion. We set the restart probability *r* of RWR from 0.1 to 0.9 and tested the effect of different *r* values on the results. We found that the results of scapGNN were less affected by choosing different values of *r* (S34E Fig). An *r* value of 0.7 allows scapGNN to maintain relatively good performance. We have modified the following:

(1) Page 34, section "Calculating pathway activity scores and identifying gene modules" in the Methods.

"Kohler et al.’s research showed that $r$ had only a slight effect on the results of the RWR algorithm when it fluctuated between 0.1 and 0.9 [87]. This is also confirmed by our benchmark experiments for the *r* values (S34E Fig). In this study, we set $r=0.7$ which has relatively better performance (S34E Fig)."

(2) Page 57, S34 Fig in the Supporting information.

*3. Whether the three networks (gene-gene association network, cell-cell association network, gene-cell association network) on which the random walk is based can be compared at the same level?*

**REPLY:** Thank you for your question. Yes, the three networks can be compared on the same level because they are all based on the end-to-end autocoder. Our framework has a uniform data input and we set up the data transfer in the latent space to maintain consistency in the learning process of the autoencoders. Unsupervised autoencoders, which include the encoding-decoding process, have a key property that the learned representations or reconstitutions retain the essential original information and distribution. Additionally, we have performed a normalization step to ensure that the networks have a consistent scale.

*4. What’s the significance of* *constructing the cell community network? What can users use it to do? Please prove to construct it is significant.*

**REPLY:** Thank you for your question. One of the most important features in the network is the existence of the community or cluster structure which is defined as a group of nodes having similar affiliations different to rest of the network. In this study, we identify cell communities in the cell-cell association network by the community detection algorithm. Cells from the same community may tend to have similar biological functions. We merge cells that belong to the same cell type or cell community that we have identified to construct the cell community network. In this way, it will be feasible to demonstrate the strength of the association between different cell types or cell communities. Using the time series dataset as an example, the association between cells at the differentiation end (36 h, 72 h, and 96 h) of the definitive endoderm cells (DEC) was stronger (S6A Fig). This indicates that the cells at these timescales have diminished differentiation drive and more similar cellular functions. For the cell communities, we identified in the cell association network, early differentiatingDEC and late differentiating DEC tend to be in different clusters (S6B Fig).

We have modified the following:

(1) Page 10, section "The overview of the scapGNN framework" in the Results.

"We can also cluster the cells into communities using three community structure detection method algorithms in the cell-cell association network (Fig 1B and Methods). We merge the same cell types or identified cell communities to construct cell community networks from the cell-cell association network. Edges between nodes in this network indicate the strength of similar associations between cell types or cell communities."

(2) Page 12, section "The pathway activity score of scapGNN can represent cell heterogeneity" in the Results.

"Our framework has the ability to merge the same cell types or identified cell communities in the cell-cell association network to observe the strength of association between them. For the time series dataset, the association between cells at the differentiation end (36 h, 72 h, and 96 h) of the definitive endoderm cells (DEC) was stronger (S6A Fig). This indicates that the cells at these timescales have diminished differentiation drive and more similar cellular functions. For the cell communities we identified in the cell association network, early differentiatingDEC and late differentiating DEC tend to be in different clusters (S6B Fig)."

(3) Page 51, S6 Fig in the Supporting information.

*5. Why use multi-omics to explain mechanisms at the pathway level? Can't we effectively analyze pathways using only scRNA-seq data? There are not enough experiments to show that using multi-omics data can provide a better improvement than single-omics data. The proof based on only one set of data is not convincing. Moreover, the only two cell alignment methods (Cobolt and GLUE) which scapGNN is compared with in terms of cell clustering performance are not enough. There are many methods that can do cell alignments, such as Seuratv4, MOFA2.0, TotalVI, and Deepmaps. And due to these methods, all using deep learning models, these methods can all be compared by means of adjusting hyperparameters, which makes it more convincing that scapGNN is better than others. Besides the experiments done on scRNA-seq datasets, should be also used on multi-omics datasets, such as the ability to compute pseudo time, the ability to find subclusters, the ability to find the true active pathways on homogeneous and heterogeneous data, and the ability to identify cell phenotype-associated gene modules. Through these experiments, you can prove that the scapGNN model based on multi-omics data is better than the scapGNN model and other methods based on single-omics data.*

**REPLY:** Thank you for your suggestion. Multi-omics datasets represent distinct aspects of the central dogma of molecular biology. The analysis of single omics data and the integration of multi-omics data are separate studies with different purposes. The analysis of single omics data, such as scRNA-seq data, is certainly desirable. By inferring pathway activity from scRNA-seq data, we can identify the pathways associated with changes in mRNA levels in cells. The maintenance of human health and the development of disease are the results of complex biological processes working in synergy (DOI: 10.1177/1177932219899051). Some complex biological pathways that cross-talk with multiple omic layers are difficult to identify from a single-omics (DOI: 10.1016/bs.adgen.2015.11.004 and DOI: 10.1016/j.tig.2021.08.012). We can identify those pathways that are omics-specific or multi-omics-associated based on multi-omics-supported pathway activity.

We further added a single-cell multi-omics data integration method to compare performance. The parameters and procedures used for these single-cell multi-omics data integration methods are as recommended by their protocols, and detailed information can be found in S2 Table. Unfortunately, the Deepmaps method requires computer configurations far beyond those available to us when integrating single-cell multi-omics data. Therefore, we cannot implement the method for the time being. The TotalVI method appears to be suitable only for CITE-seq data, which cannot integrate scRNA-seq and scATAC-seq data. In addition, we evaluated the performance of scapGNN-inferred single-cell multi-omics-supported pathway activity scores in terms of pathway identification accuracy and cellular timing representation. The relevant revisions are as follows:

(1) Page 19, section "Inferring pathway activity scores by integrating scRNA-seq and scATAC-seq" in the Results.

"Next, we tested the performance of scapGNN to integrate single-cell multi-omics data. To evaluate the ability of the single-cell multi-omics supported pathway activity scores inferred by scapGNN to represent the cellular heterogeneity, We applied scapGNN to three single-cell multi-omics datasets with different sequencing platforms, different tissues, and species (S5 Table). UMAP plots of multi-omics supported pathway activity scores show that scapGNN can clearly distinguish between different cell types after integrating single-cell multi-omics information (S21A-C Fig). And compared to five state-of-the-art single-cell multi-omics integration methods (S2 Table), scapGNN shows excellent performance on several cell clustering indices (Fig 6A and B, S21D Fig).

In hair follicle tissue, transit-amplifying cells (TAC) can proliferate rapidly and produce three different mature cells, including the inner root sheath (IRS), hair shaft cuticle/cortex layer and medulla [48]. Recently, Jones et al. has applied SHARE-seq, an approach that enables the joint measurement of chromatin accessibility and gene expression from the same single cells to adult mouse skin tissue and resolved the differentiation process of TAC [48]. We applied scapGNN to integrate scRNA-seq and scATAC-seq data from the mouse skin dataset and to infer the pseudotime of TAC differentiation using Monocle 3 [49]. Compared to scDART (S2 Table), it is a method for integrating scRNA-seq and scATAC-seq data and can be used for trajectory inference on integrated data, scapGNN can show the differentiation trajectory of TAC more accurately (Fig 6C and S22 Fig). The pseudotime based on single-cell multi-omics supported pathway activity scores calculated by scapGNN has a higher similarity to the TAC differentiation pseudotime provided by Jones’s study [48] compared to scDART (Fig 6D).

We found that some significantly differential pathways were only found in the results by integrating multi-omics data, and those pathways have been documented to be involved in key biological functions of astrocytes and oligodendrocytes in the mouse brain cortex dataset (S6 Table). For T and B cells in the PBMC multi-omics dataset, the proportion of cells with T cell receptor signaling pathway or B cell receptor signaling pathway in the top 5 of the single-cell multi-omics supported pathway activity score ranked list was more than 0.95 (S23A Fig). For single-cell multi-omics data integration, scapGNN is also able to maintain the stability of pathway scoring (S24 Fig). Using genes in the T cell receptor signaling pathway or B cell receptor signaling pathway as the gold standard, the gene modules identified by scapGNN for T cells or B cells were able to accurately identify these genes with AUCs above 0.8 (S23B Fig). There was a consistent trend in the results for marker gene sets, with scapGNN being able to accurately identify the marker gene set corresponding to the cell type in both heterogeneous and homogeneous data (S23C Fig). ROC curves show that scapGNN can accurately identify highly active genes in the gene module using the marker gene set as the gold standard (S23D Fig). A number of studies have shown that the hedgehog signaling pathway is required for TAC developmental processes [48, 50, 51]. In particular, the transcriptome and epigenome activity of Gli2 and Gli3 in the hedgehog signaling pathway plays a critical role [50, 51]. This is confirmed by the violin plots of Gli2 and Gli3 activity in RNA and DNA in the mouse brain cortex dataset (S25A and B Fig). Consistent with this, scapGNN also detected high single-cell multi-omics supported pathway activity scores of the hedgehog signalling pathway during TAC development (S25C Fig). Gli2 and Gli3 also tend to co-expression during TAC development in the gene modules identified based on the combined gene-cell association network (S25D Fig).

These results demonstrate that scapGNN can effectively integrate single-cell multi-omics information at the pathway level to cluster cells, infer cell differentiation processes and accurately identify highly active pathways and cell phenotype-associated gene modules."

(2) Page 50, Fig 6 in the Manuscript.

(3) Page 54, S21 Fig in the Supporting information.

(4) Page 55, S22 Fig in the Supporting information.

(5) Page 55, S23 Fig in the Supporting information.

(6) Page 55, S24 Fig in the Supporting information.

(7) Page 55, S25 Fig in the Supporting information.

*6. The title of this article is “scapGNN: a graph neural network-based framework for active pathway and gene module inference from single-cell multi-omics data”. However, most of the benchmark experiments in this paper are based on scRNA-seq data, and the only case study is also based on scRNA-seq data. Please supplement the benchmark experiment and case study based on multi-omics data suffice to show that this article is designed for multi-omics data.*

**REPLY:** Thank you for your suggestion. There are no benchmark methods for single-cell multi-omics integration methods and, in particular, for integrating single-cell multi-omics data to infer pathway activity. However, to better evaluate the performance of scapGNN for single-cell multi-omics data integration, we applied scapGNN to three single-cell multi-omics datasets with different sequencing platforms, different tissues, and species (S5 Table). Six advanced single-cell multi-omics data integration methods were selected to compare the performances (S2 Table). We have systematically evaluated scapGNN in terms of cell clustering, cell pseudotime, and pathway identification accuracy for single-cell multi-omics data integration. The results show that scapGNN also has excellent performance for single-cell multi-omics data integration (Fig 6, S21 Fig, S22 Fig, and S23 Fig). For both single-cell mono-omics and integration of single-cell multi-omics data to infer pathway activity, our method is based on the constructed gene-cell association network. The benchmark results for scapGNN on single-omics data demonstrate that our approach to transform the gene-cell matrix into a gene-cell association network is feasible and that the gene-cell association network can capture gene-cell, gene-gene, and cell-cell relationships well. Therefore, the benchmark experiments of scapGNN in single-omics data are instructive for single-cell multi-omics data integration. Refer to Comment 5 for the relevant amendments.

*7. Are all the experiments in this paper based on the same set of parameters of scapGNN and other benchmark methods? Do other benchmark methods use their default parameters? Please provide a parameter description.*

**REPLY:** Thank you for your suggestion. In this study, scapGNN and other benchmark methods were used with a consistent set of parameters for all experiments. The methods we use for performance comparisons use either the default parameter settings or those recommended in their manuals. S2 Table summarises the specific parameters of each method and the link to their manuals. The relevant revisions are as follows:

(1) Page 58, S2 Table in the Supporting information.

*8. Please compare these methods’ performance on a grid of hyperparameters to prove this method outperforms other methods not just on a particular set of parameters and compare the robustness of the methods to the parameters.*

**REPLY:** Thank you for your suggestion. Based on your suggestion, we evaluated the hyperparameters of scapGNN in terms of both cell clustering and pathway identification. Except for DNNAE, which is unable to perform gradient descent at higher learning rates (≥0.01), scapGNN has shown stability in its parameter settings. Overall, scapGNN performs relatively well with the default parameter settings. The relevant revisions are as follows:

(1) Page 57, S34 Fig in the Supporting information.

*9. Prove that LTMG helps improve model performance in ablation experiments*

**REPLY:** Thank you for pointing this out. We remove the LTMG regularization using only the DNNAE and GAE results (scapGNN-LTMG). The scapGNN has improved performance compared to the scapGNN-LTMG (S11 Fig). The relevant revisions are as follows:

(1) Page 53, S12 Fig in the Supporting information.

*10. Version targeting single-cell pathway enrichment analysis (ssGSEA) are available in GSEA. Why doesn’t the user choose ssGSEA to get single-cell pathway enrichment? Please explain if there are any shortcomings in its method and do an experiment to compare with it.*

**REPLY:** Thank you for your suggestion. Some extension methods of GSEA, such as ssGSEA and GSVA, can assess changes in the activity of pathways in a single sample of bulk RNA-seq data. They rank gene expression within each sample separately and calculate the pathway activity score using K-S like random walk statistic. They are very sensitive to the library scale of the data. However, compared to RNA-seq data obtained from bulk cell populations, single-cell sequencing data are much sparser, noisier, and lower in library size (DOI: 10.1093/bib/bbab147). These will seriously compromise the accuracy and integrity of gene sequencing. Consistent with Su's study (DOI: 10.1016/j.csbj.2020.10.007), ssGSEA and GSVA perform poorly on single-cell datasets (S7 Fig). The relevant revisions are as follows:

(1) Page 52, S7 Fig in the Supporting information.

(2) Page 2, section "Introduction".

"Pathway activation analysis in single-cell studies for identifying biologically significant patterns that can help uncover the underlying causes of cell heterogeneity and dysfunction in human diseases [8, 9]. However, the current pathway enrichment analysis methods (e.g. GSEA [10], ssGSEA [11], and GSVA [12]) developed for bulk RNA-seq data have been reported to be inappropriate for single-cell sequencing data [13, 14]. Compared to RNA-seq data obtained from bulk cell populations, single-cell sequencing data are much sparser, noisier, and lower in library size due to the particular sequencing techniques and experiment protocols [15]. These will seriously compromise the accuracy and integrity of gene-level analyses in single-cell data [1]. Hence, there is an urgent need for an efficient method to parcel out the pathway activity of individual cells."

*11. Please explain the significance of constructing a pathway activity matrix on a single-cell level. Can use it to replace a single-cell expression matrix on single-cell data analysis? If so, please prove it will have a significantly better performance on downstream analysis such as cell clustering, and trajectory inference, than based on a single-cell expression matrix.*

**REPLY:** Thank you for your suggestion. We believe the main importance of performing pathway activity analysis in single-cell data is as follows: i) Single-cell data has a large number of drop-out events where a gene expression is supposed to exist but not detected due to the particular sequencing techniques and experiment protocols. This leads to significant difficulties in analyzing single-cell data at the gene level (DOI: 10.1038/s41576-019-0095-5). For example, most single-cell clustering methods only use genes as a feature of cells, ignoring the relationship between genes, which could make clustering methods more susceptible to noise, resulting in low accuracy and robustness. The noise on a single gene may have a relatively small impact on the entire pathway (DOI: 10.1093/bib/bbab147). ii) A canonical pathway, which is curated from reliable literature resources such as the KEGG database, is a collection of relationships between genes that regulate the same biological process. This allows the pathway to have better biological interpretation and gain insights into biological mechanisms for the cell (DOI: 10.1073/pnas.0506580102).

Our study demonstrates that the pathway activity score matrix can be used as an alternative to single-cell expression matrix for single-cell data analysis, such as cell clustering, and temporal sequencing analysis. Moreover, the pathway activity scores inferred by scapGNN can improve the performance of single-cell data analysis (Fig 2D and S4 Fig). The relevant revisions are as follows:

(1) Page 2, section "Introduction".

"Pathway activation analysis in single-cell studies for identifying biologically significant patterns that can help uncover the underlying causes of cell heterogeneity and dysfunction in human diseases [8, 9]. However, the current pathway enrichment analysis methods (e.g. GSEA [10], ssGSEA [11], and GSVA [12]) developed for bulk RNA-seq data have been reported to be inappropriate for single-cell sequencing data [13, 14]. Compared to RNA-seq data obtained from bulk cell populations, single-cell sequencing data are much sparser, noisier, and lower in library size due to the particular sequencing techniques and experiment protocols [15]. These will seriously compromise the accuracy and integrity of gene-level analyses in single-cell data [1]. Hence, there is an urgent need for an efficient method to parcel out the pathway activity of individual cells."

(2) Page 26, section "Discussion".

"Pathway enrichment analysis is a wide framework for condensing information from gene expression profiles into a pathway or signature summary [12]. Pathway-level analysis of single cells improves signal-to-noise ratio and biological interpretation relative to analysis based on gene features [1, 10]."

(3) Page 51, S4 Fig in the Supporting information.

*12. In S12 , did the experiments on T cell +epithelial cell and B cell +epithelial cell eliminate the batch effect? For the experiment in S12 Fig, why not use a PBMC dataset? The T cell +epithelial cell doesn’t have enough heterogeneity. Please do this experiment on a PBMC dataset that contains multiple cell types and prove it will also have a better performance based on a multi-omics dataset.*

**REPLY:** Thank you for your suggestion. In S12 (now S14 Fig), we assessed the stability of scapGNN pathway scoring. We recombine T cells or B cells with other cells into a new dataset. In these datasets, we reassessed the identification ability of scapGNN for both the T cell receptor signaling pathway and the B cell receptor signaling pathway. Our results show that scapGNN still maintains a consistent level of performance on these recombinant cell type datasets when compared to the original data (S14 Fig, S20 Fig, and S24 Fig). This suggests that scapGNN can accurately detect the true pathway of the corresponding cell and is not influenced by other cell types in the dataset. Following your suggestion, we have added test results of scRNA-seq data and single-cell multi-omics of the PBMC multi-omics dataset, as well as other cell types for combination with T and B cells. The relevant revisions are as follows:

(1) Page 15, section "scapGNN accurately identified active pathways and cell phenotype-associated gene modules" in the Results.

"We assessed the stability of scapGNN pathway scoring by grouping T cells or B cells with different cells. The results show that scapGNN can consistently identify the T cell receptor signaling pathway and the B cell receptor signaling pathway, even in different combinations of datasets (S14 Fig)."

(2) Page 20, section "Inferring pathway activity scores by integrating scRNA-seq and scATAC-seq" in the Results.

"For single-cell multi-omics data integration, scapGNN is also able to maintain the stability of pathway scoring (S24 Fig)."

(3) Page 53, S14 Fig in the Supporting information.

(4) Page 54, S20 Fig in the Supporting information.

(4) Page 55, S24 Fig in the Supporting information.

*13. On line 288, why use the biomarkers of cell clusters identified by Seurat v4 as the gold standard and not cell type marker genes in the databases? If the datasets don’t have cell type marker genes information in the databases, why not use datasets whose cell types have recognized marker information? This is also for robustness experiments.*

**REPLY:** Thank you for your suggestion. We have revised the content. We performed ROC analyses on homogeneous and heterogeneous data using marker pathways and marker genes as gold standards to assess the performance of scapGNN in identifying phenotype-associated gene modules (Fig 3C-E). In addition, we added a noise ratio to the data to test the accuracy of scapGNN in identifying gene modules associated with cellular phenotypes (S15 Fig). The relevant revisions are as follows:

(1) Page 16, section "scapGNN accurately identified active pathways and cell phenotype-associated gene modules" in the Results.

"For homogeneous and heterogeneous data, we calculated the association scores of cell types with genes using cells belonging to the same type as a seed. These association scores were sorted in descending order. Using the marker genes of the corresponding cell type or the genes of the T and B cell receptor signaling pathway as the gold standard, the receiver-operating characteristic (ROC) curve analysis was used to assess the accuracy of scapGNN in identifying phenotype-associated gene module. In addition, we used genomap, an entropy-based cartography method for discovering cell- and class-specific gene sets from scRNA-seq data, as a benchmark method (S2 Table) [21]. The genomap gets activation values for each gene in the specified cell type. However, the training of the genomap model relies on the known truth cell labels of the dataset. When the labels of the cell types are all the same (i.e., homogeneous data), the genomap gets zero activity values for all genes. Therefore, we can only compare the performance of scapGNN and genomap on heterogeneous data. The results, as shown in Fig 3C-E, indicate that scapGNN can identify cell type-associated active genes either by marker pathway or by marker gene as a gold standard. And the accuracy and robustness of scapGNN are better than genomap (Fig 3C-E and S15 Fig). An additional advantage of scapGNN over genomap is the ability to provide significance-level p-values for the association scores of each gene with cell phenotypes."

(2) Page 49, Fig 3 in the Manuscript.

(3) Page 53, S15 Fig in the Supporting information.

*14. Please compare with other software that do cell phenotype-related gene modules. This is also for robustness experiments.*

**REPLY:** Thank you for your suggestion. We used genomap, an entropy-based cartography method for discovering cell- and class-specific gene sets from scRNA-seq data, as a benchmark method. The genomap gets activation values for each gene in the specified cell type. However, the training of the genomap model relies on the known truth cell labels of the dataset. When the labels of the cell types are all the same (i.e., homogeneous data), the genomap gets zero activity values for all genes. Therefore, we can only compare the performance of scapGNN and genomap on heterogeneous data. And the accuracy and robustness of scapGNN are better than genomap (Fig 3C-E and S15 Fig). An additional advantage of scapGNN over genomap is the ability to provide significance level p-values for the association scores of each gene with cellular phenotypes. The relevant revisions are as follows:

(1) Page 16, section "scapGNN accurately identified active pathways and cell phenotype-associated gene modules" in the Results.

"For homogeneous and heterogeneous data, we calculated the association scores of cell types with genes using cells belonging to the same type as a seed. These association scores were sorted in descending order. Using the marker genes of the corresponding cell type or the genes of the T and B cell receptor signaling pathway as the gold standard, the receiver-operating characteristic (ROC) curve analysis was used to assess the accuracy of scapGNN in identifying phenotype-associated gene module. In addition, we used genomap, an entropy-based cartography method for discovering cell- and class-specific gene sets from scRNA-seq data, as a benchmark method (S2 Table) [21]. The genomap gets activation values for each gene in the specified cell type. However, the training of the genomap model relies on the known truth cell labels of the dataset. When the labels of the cell types are all the same (i.e., homogeneous data), the genomap gets zero activity values for all genes. Therefore, we can only compare the performance of scapGNN and genomap on heterogeneous data. The results, as shown in Fig 3C-E, indicate that scapGNN can identify cell type-associated active genes either by marker pathway or by marker gene as a gold standard. And the accuracy and robustness of scapGNN are better than genomap (Fig 3C-E and S15 Fig). An additional advantage of scapGNN over genomap is the ability to provide significance-level p-values for the association scores of each gene with cell phenotypes."

(2) Page 49, Fig 3 in the Manuscript.

(3) Page 53, S15 Fig in the Supporting information.

*15. What significant conclusion can the user get by identifying the differentially active pathways between the cell types? Or what can we do by using the differentially active pathways between the cell types?*

**REPLY:** Thank you for your question. scapGNN transforms gene expression matrix data into a pathway activity score matrix at the single cell level. This allows us to replace the analysis of individual genes at the pathway level. Biological pathways in distinct cell types have different activation patterns, which facilitates the understanding of cell functions. In single-cell studies, pathway activation analysis has become a powerful approach for the extraction of biologically relevant signatures to uncover the potential mechanisms of cell heterogeneity and dysfunction in human diseases (DOI: 10.1126/science.aau5324 and DOI: 10.1038/s41467-019-11738-0). For example, pathway signatures exhibit an activation difference in breast cancer (DOI: 10.1038/ncomms15081) and Alzheimer’s disease cells (DOI: 10.1038/s41593-019-0539-4).

In the present study, we found that the oxidative phosphorylation pathway had significantly higher activity from early pachytene (eP) spermatocytes to early round spermatids (RS2 to RS4) by analyzing differences between cell types (Fig 7B). During this time, spermatocyte need to break through the blood-testis barrier, which takes a lot of energy. Oxidative phosphorylation is the main energy supply pathway under normal physiological conditions, and it happens to provide energy for this process of spermatocyte differentiation. And the mechanism of action of the oxidative pathway on spermatocyte differentiation has been confirmed by existing studies (DOI: 10.7554/eLife.51601

).

The setting of the cellular phenotype is flexible in the analysis of variance. In addition to cell type, we were able to identify pathways that differed between sample phenotypes. We applied the scapGNN to the coronavirus disease 2019 (COVID-19) dataset, which includes healthy control and COVID-19 patient. We screened B-cell-associated differential pathways between healthy controls and COVID-19 patients. Consistent with previous studies, protein complex assembly, and protein transport-related pathways are activated in the B cells of COVID-19 patients, as B cells require high levels of protein synthesis to perform their functions (DOI: 10.1016/j.immuni.2020.07.009) (S30 Fig). We also found that thyroid hormone synthesis pathway activity was significantly elevated in COVID-19 patient relative to healthy control (S30 Fig). Many reports have shown that thyroid hormone has a protective effect on COVID-19 patient and that low serum levels may increase mortality (DOI: 10.3389/fendo.2021.779692 and DOI: 10.1007/s40618-021-01506-7).

Furthermore, due to the limitations of sequencing technology and the heterogeneity of cells, single-cell data contain many zero values, in which case differential analysis of individual genes can result in low accuracy and robustness. For single-cell analyses at the pathway level, the noise on a single gene may have a relatively small impact on the entire pathway (DOI: 10.1093/bib/bbab147).

*16. Other software uses cell phenotype-related gene modules to do cell type-related pathway enrichment or study the regulatory mechanisms related to cell type or others. The case study didn’t show what significant results users can get by using cell type-related gene modules.*

*17. In the case study, what biological significance do the genes that were found associated with multiple cell types represent? What research can users do with them?*

**REPLY:** Thank you for your suggestions. This response is to Comment 16 and 17. In contrast to pathways based on a priori knowledge, the gene modules were spontaneously identified by the RWR algorithm using cells with the same phenotype as the initial nodes. We quantified the association strength of each gene with the cell phenotype and obtained the significance level by perturbation analysis. The gene module is the set of genes that are significantly most important for the characterization of the identity of that cell phenotype. For multiple cell phenotypes, we also quantified the propensity of genes to be expressed between cell phenotypes and provide a visualization program for the network of cell phenotype-associated gene modules. We can gain insight into cell phenotype-specific genes, the co-expression of multiple cell phenotype genes, and the strength of gene-gene associations.

For example, genes such as *Prm1*, *Prm2*, and *Odf2* are specifically expressed at the RS8 stage in the spermatogenesis stage-associated gene modules network (Fig 7D). Existing studies have also reported these genes as round sperm-specific marker genes capable of characterizing it (DOI: 10.3390/cells10030666, DOI: 10.1074/jbc.272.15.10327, and DOI: 10.1016/j.jprot.2015.08.001). Genes associated with multiple cell types such as *Fabp9* that is highly expressed from the MI spermatocytes to the round spermatids stage. And *Fabp9* is essential for the formation of the normal shape of the sperm head (DOI: 10.1016/j.ydbio.2010.09.019). In addition, for the disease models, we constructed gene module networks for B cells of three cell phenotypes (healthy control, COVID-19, and IAV) (S29C Fig). We have identified potentially highly active gene signatures of COVID-19 and therapeutic targets such as *NPM1* and *ARPC1B*. The relevant revisions are as follows:

(1) Page 9, section "The overview of the scapGNN framework" in the Results.

"The gene module is the set of genes that are significantly most important for the characterization of the identity of that cell phenotype. For multiple cell phenotypes, we also quantified the propensity of genes to be expressed between cell phenotypes and provide a visualization program for the network of cell phenotype-associated gene modules. We can gain insight into cell phenotype-specific genes, or genes expressed in multiple cell phenotypes, and the strength of gene-gene associations. The foundation of the scapGNN framework is to infer the gene-cell association network based on gene expression features in cells. Hence, the biological significance conveyed by the associations between genes is the co-expression regulatory relationships."

(2) Page 23, section "Applications of scapGNN in spermatogenesis and early embryo development" in the Results.

"For example, *Fabp9* is high co-expressed from the MI spermatocytes to the round spermatids stage and is essential for the formation of the normal shape of the sperm head [63]. Among the network of spermatogenesis stage-associated gene modules, RBAK downstream neighbor (Rbakdn) and spermatogenesis associated 33 (Spata33) were the two genes with the highest association scores with *Fabp9*, and their expression patterns showed a clear co-occurrence with *Fabp9* (S28A and B Fig). Deletion of Spata33 was also reported to cause abnormalities in sperm formation with sperm midpiece defects and infertility [64]. In comparison, cysteine-rich perinuclear theca 12 (Cypt12) and high mobility group box 4 (Hmgb4) are two genes with a lower strength of association with *Fabp9*, and they did not have a consistent expression trend with *Fabp9* (S28C and D Fig)."

(3) Page 25, section "Investigation of the biological mechanisms in COVID-19 based on scapGNN.

"We further integrated the scRNA-seq data of influenza A virus (IAV)-infected patient and constructed gene modules networks for B cells of three cell phenotypes (healthy control, COVID-19, and IAV) using false discovery rate (FDR) < 0.05 (S29C Fig). Genes co-expressed in the three cellular phenotypes may be disease independent. COVID-19-specific genes may be potentially high-activity gene signatures of COVID-19. For example, NPM1 is present in the COVID-19-associated PPI network module and is associated with enhanced viral replication of COVID-19 [74, 75]. COVID-19 and IAV co-expressed genes may be associated with essential biological functions of B cells, for example IGHG3 and IGLC2 are B cell marker genes [76]. Interestingly, the absence of ARPC1B, a health-specific gene that is reduced in COVID-19, leads to immunodeficiency [77, 78]. This may provide new insights into the pathogenic mechanisms and treatment of COVID-19."

(5) Page 56, S28 Fig in the Supporting information.

(6) Page 56, S29 Fig in the Supporting information.

(7) Page 57, S30 Fig in the Supporting information.

*18. In line 414, “The cell phenotype-associated gene networks discovered the regulatory mechanisms between cell types” didn’t be proven. How can we get regulatory mechanisms from the gene-gene edge in the network of cell phenotype-associated gene modules? Can the edges between nodes give the user information on the regulatory relationship between genes? Please improve it.*

**REPLY:** Thank you for your suggestion. The foundation of the scapGNN framework is to infer the gene-cell association network based on gene expression features in cells. Hence, the biological significance conveyed by the associations between genes is the co-expression regulatory relationships in the context of a specific cell phenotype. In the case of the gene *Fabp9* in the network of spermatogenesis stage-associated gene modules, *Rbakdn* and *Spata33* were the two genes with the highest association scores with *Fabp9*, and their expression patterns showed a clear co-occurrence with *Fabp9* (S28A and B Fig). Deletion of Spata33 was also reported to cause abnormalities in sperm formation with sperm midpiece defects and infertility. In comparison, *Cypt12* and *Hmgb4* are two genes with a lower strength of association with *Fabp9*, and they did not have a consistent expression trend with *Fabp9* (S28C and D Fig). The relevant revisions are as follows:

(1) Page 9, section "The overview of the scapGNN framework" in the Results.

"The gene module is the set of genes that are significantly most important for the characterization of the identity of that cell phenotype. For multiple cell phenotypes, we also quantified the propensity of genes to be expressed between cell phenotypes and provide a visualization program for the network of cell phenotype-associated gene modules. We can gain insight into cell phenotype-specific genes, or genes expressed in multiple cell phenotypes, and the strength of gene-gene associations. The foundation of the scapGNN framework is to infer the gene-cell association network based on gene expression features in cells. Hence, the biological significance conveyed by the associations between genes is the co-expression regulatory relationships."

(2) Page 23, section "Applications of scapGNN in spermatogenesis and early embryo development" in the Results.

"For example, *Fabp9* is high co-expressed from the MI spermatocytes to the round spermatids stage and is essential for the formation of the normal shape of the sperm head [63]. Among the network of spermatogenesis stage-associated gene modules, RBAK downstream neighbor (Rbakdn) and spermatogenesis associated 33 (Spata33) were the two genes with the highest association scores with *Fabp9*, and their expression patterns showed a clear co-occurrence with *Fabp9* (S28A and B Fig). Deletion of Spata33 was also reported to cause abnormalities in sperm formation with sperm midpiece defects and infertility [64]. In comparison, cysteine-rich perinuclear theca 12 (Cypt12) and high mobility group box 4 (Hmgb4) are two genes with a lower strength of association with *Fabp9*, and they did not have a consistent expression trend with *Fabp9* (S28C and D Fig)."

(3) Page 56, S28 Fig in the Supporting information.

*Presentation issues:
1. W^l in equations (2) and (3) should be represented by different symbols.*

**REPLY:** Thanks for pointing that out. We have revised the relevant content as follows:

(1) Page 31, section "Deep neural network autoencoder" in the Methods.

"$E_{m\times d}^{g}=\sigma\left( W_{g}^{l}X \right)$	(2)

$E_{n\times d}^{c}=\sigma\left( {W_{c}^{l}X}^{T} \right)$	(3)

where $W_{g}^{l}$ and $W_{c}^{l}$ is the learnable weight of the $l$th hidden layer for gene embedding and cell embedding."

*2. The second item in equation(5) is problematic.*

**REPLY:** Thanks for pointing that out. We have amended the formula. The modifications are as follows:

(1) Page 232, section "Deep neural network autoencoder" in the Methods.

"The loss function is as follows:

$loss=\left( 1-a \right)||X^{'}-X{||}_{2}^{2}+a\left( \sum(X^{'}-{X)}^{2}^{\circ}S_{TRS} \right)$	(5)

where *a* is the regularization weight and *a*∈[0,1]. $^{\circ}$ denotes element-wise multiplication. $S_{TRS}\in R^{m\times n}$ is the transcriptional regulatory signal matrix."

*3. On page 28, “**We obtain the stationary probability vector by iterating repeatedly until the difference between p_(t+1) and p_t falls below 1×10^(-6).” How to evaluate the difference between p_(t+1) and p_t by a single value.*

**REPLY:** Thanks for pointing that out. We take the absolute value of the difference between the corresponding elements of two matrices ($p_{t+1}$ and $p_{t}$) and sum them to less than ${1\times10}^{-6}$. The relevant revisions are as follows:

(1) Page 35, section "Calculating pathway activity scores and identifying gene modules" in the Methods.

"We obtain the stationary probability vector by iterating repeatedly until the difference between $p_{t+1}$ and $p_{t}$ falls below ${1\times10}^{-6}$, i.e. $\sum\left| p_{t+1}-p_{t} \right|<{1\times10}^{-6}$."

*4. Do the edges between genes in the network of cell type-associated gene modules come from the gene-gene association network, and what does the width of an edge represent?*

**REPLY:** Thanks for pointing that out. In the network of cell phenotype-associated gene modules, the sector area of a node indicates the strength of association between a gene and a cell phenotype, and the width of the edges between nodes indicates the strength of association between genes. The relevant revisions are as follows:

(1) Page 50, Fig 7 in the Manuscript.

"Network of cell phenotype-associated gene modules during mouse spermatogenesis (**D**) and embryo development (**E**). In the network, the sector area of a node indicates the strength of association between a gene and a cell phenotype, and the width of the edges between nodes indicates the strength of association between genes."

(2) Page 56, S27 Fig in the Supporting information.

*5. Please explain clearly how to use single-cell pathway enrichment methods with cell clustering methods. Is it replacing the expression matrix with the pathway activity matrix?*

**REPLY:** Thank you for your suggestion. For cell clustering evaluation, we used the pathway activity score matrix obtained from different single-cell pathway activity scoring methods as input to the sciPath framework. The sciPath framework was developed to study the impact on accuracy and robustness of existing single-cell clustering methods by integrating pathways based on 10 state-of-the-art single-cell clustering methods and three accuracy indicators (DOI: 10.1093/bib/bbab147). The procedures and parameters organized by the sciPath framework were used. The computer codes are available at https://github.com/GaoLabXDU/sciPath. The relevant revisions are as follows:

(1) Page 37, section "Cell clustering performance evaluation" in the Methods.

"We used the pathway activity score matrix obtained from different single-cell pathway activity scoring methods as input to the sciPath framework. The 10 state-of-the-art single-cell clustering methods (S4 Table) in the sciPath framework were used to infer cell clustering from the pathway activity score matrix."

*6. Please provide the formula for calculating BCMI.*

**REPLY:** Thank you for your suggestion. We have added the change to the supplementary methods. The relevant revisions are as follows:

(1) Page 51, S1 Text in the Supporting information.

"Let *X* be the true cellular timestamps, the probability $P\left( X=x_{i} \right)=p_{i}, i\in\{1,\ldots,g\}$, *g* denotes the number of groups in *X*. Let Y be the inferred pseudotime with density function $f(y)$. For each $x_{i}$ , we have a continuous density function $f_{Y|X=x_{i}}=x_{i}\left( y \right),$ is simply denoted as $f_{i}(y)$. The mutual information is calculated as follows:

$I\left( X, Y \right)=\sum_{i=1}^{g} p_{i}\int_{y\mathfrak{\in R}}f_{i}(y)log(\frac{f_{i}(y)}{f(y)})dy$	(S1)

The jackknife, which is a nonparametric statistical procedure that can be thought of as a computational simplification of the bootstrap, was used to gives a correction for estimation bias. A high BCMI value indicates the pseudotime is more similar to the true time series.

For single-cell multi-omics data integration in time-series analyses using mouse skin dataset, the pseudotime provided by the Jones study, the scapGNN, and scDART inferred were continuous. Therefore, the Pearson correlation was used to assess the similarity."

*7. Please explain how* *Brown’s method works and why can use to integrate gene-gene, cell-cell, and gene-cell networks.*

**REPLY:** Thank you for your suggestion. Brown's method was first used to combine multiple dependent statistical tests (https://doi.org/10.2307/2529826). A growing number of studies have used it for tasks such as multi-omics integration or calculating pathway scores by combining the expression values of genes in the pathways (DOI:10.1038/s41467-019-13983-9 and doi: 10.1093/bioinformatics/btw438). Brown’s method considers dependencies between datasets and thus provides more conservative estimates of significance for genes that are supported by multiple similar omics datasets (DOI:10.1038/s41467-019-13983-9). The edge weights in the gene-cell association network serve as indicators of significance between nodes, such as the strength of co-expression between genes, the level of gene expression in cells, and the similarity between cells. After integrating gene-cell association networks from different omics, the weights of the edges account for the overall covariation of the weights from different sources of evidence. Furthermore, we used the RWR algorithm to calculate the proximity between genes in the pathway and each cell as the multi-omics information-supported pathway activity scores, and the proximity between cells with the same phenotype and each gene to identify cell phenotype-associated gene modules. The relevant revisions are as follows:

(1) Page 36, section "Integrating single-cell multi-omics data into pathway activity score matrix" in the Methods.

"Brown's method was first used to combine multiple dependent statistical tests [90]. A growing number of studies have used it for tasks such as multi-omics integration or calculating pathway scores by combining the expression values of genes in the pathways [29, 91]. Brown’s method considers dependencies between datasets and thus provides more conservative estimates of significance for genes that are supported by multiple similar omics datasets [29]. The edge weights in the gene-cell association network serve as indicators of significance between nodes, such as the strength of co-expression between genes, the level of gene expression in cells, and the similarity between cells. After integrating gene-cell association networks from different omics, the weights of the edges account for the overall covariation of the weights from different sources of evidence. Furthermore, we used the RWR algorithm to calculate the proximity between genes in the pathway and each cell as the multi-omics information-supported pathway activity scores, and the proximity between cells with the same phenotype and each gene to identify cell phenotype-associated gene modules."

*8. S8A does not significantly reflect that scapGNN can further eliminate the batch effect, especially for H1795 and H228 cell types. Please provide their separate UMAP plots or gray out the color of other types of cell points.*

**REPLY:** Thank you for your suggestion. We have divided the S8A Fig, now S10A Fig, by cell type. In the UMAP plot, one cell type in different batches is shown in different colors, while other cell types are shown in grey. The relevant revisions are as follows:

(1) Page 52, S10 Fig in the Supporting information.

*9. On line 281, “we separately set the cells of the four cell types (K562, A549, GM12878, and ESC)” have a problem. Are* *GM12878 and ESC not heterogeneous datasets? Please provide detailed information about the cell types in the datasets.*

**REPLY:** Thank you for pointing this out. The GM12878 and ESC are heterogeneous datasets. This is a confusion caused by the fact that we don't write clearly. We have revised the content as follows:

(1) Page 14, section "scapGNN accurately identified active pathways and cell phenotype-associated gene modules" in the Results.

"For homogeneous data (K562 and A549) and heterogeneous datasets (GM12878, and ESC), we counted the proportion of cells with the correct marker gene-sets of the four cell types detected in the top 5 of the pathway activity scores (S1 Table)."

*10. In Fig6, there should be UMAP visualization for scRNA-seq and scATAC-seq data.*

**REPLY:** Thank you for your suggestion. We have added the corresponding figure to Fig 6, now S21 Fig. We have revised the content as follows:

(1) Page 54, S21 Fig in the Supporting information.

*11. The legend of Fig7.E is wrong.*

**REPLY:** Thank you for pointing this out. We have corrected Fig 7E. We have revised the content as follows:

(1) Page 50, Fig 7 in the Manuscript.

*12. In line 413, S20C Fig can’t show Sertoli cells were tightly connected to spermatogenic cells.*

**REPLY:** Thank you for your question. The S20C Fig, now S27C Fig, is a cell community network. It is a fusion of nodes belonging to the same cell phenotype in the gene-cell association network. The edge weights between cell communities indicate the similarity between nodes. The weights of the edges in the graph are represented by the thickness. As shown in S6A Fig, the edges between similar nodes will be thicker. Sertoli cells are non-spermatogenesis stage cells. However, the weights of the edges between the Sertoli cell and the spermatogenesis-associated cell phenotype are similar to the weights of the edges between the spermatogenesis-associated cell phenotype. This suggests a significant association between Sertoli cells and the process of spermatogenesis.

*13. In line 416, PSAP is not in gene modules of spermatogenic cells but in Spermatic cells.*

**REPLY:** Thank you for pointing this out. We checked the available literature. Prosaposin (*PSAP*) is expressed by Sertoli cells and secreted into tissue fluid to regulate spermatogenesis (DOI: 10.3389/fendo.2022.852661, PMID: 8573994, and DOI: 10.1096/fj.14-269209). There are no studies showing the expression status of *PSAP* in cells at the stages of spermatogenesis. Although *PSAP* was not shown to be expressed in spermatogenic cells in the cell phenotype association gene module networks of S27D Fig, *PSAP* was nevertheless linked to nodes of genes expressed during the spermatogenic stage. This suggests that *PSAP* may be involved in the regulation of the expression of genes associated with the spermatogenesis stage.

*14. Fig 7A can’t clearly show scapGNN can better reconstruct the process of spermatogenesis and early embryonic development. Please provide the development sort of cell type labels and the pseudo time inferred from Molocle3 on the plot. And please draw red arrows for other methods.*

**REPLY:** Thank you for your suggestion. As the different cell types are too concentrated in some of the UMAP plots, we have added solid red arrows in the top legend to better indicate the direction of spermatogenesis. We have revised the content as follows:

(1) Page 50, Fig 7 in the Manuscript.

**Reply to Reviewer: 2**
*Reviewer #2: In the manuscript, the authors have proposed scapGNN, a graph neural network (GNN) -based framework that calculates pathway activity scores, identifies cell phenotype-associated gene modules, and integrates multi-omics data at single-cell resolution. The paper is overall well-written and easy to go through. The figures are also well-prepared. Although the problem is very important and timely, the paper has some issues that need to be addressed.*

*1. Abstract: In the abstract, lots of unnecessary sentences are added for what scapGNN does. However, it is not clear how it works. Please write as least two sentences on the novelty and major breakthrough scapGNN will bring to the data analysis pipeline. Please add a sentence on the improvement of quantitative performance of scapGNN with respect to other existing methods.*

**REPLY:** Thanks for your comments, it makes a lot of sense to us. We have completely revised the content of the summary. The revisions are as follows:

(1) Page 1, Abstract in the Manuscript.

"Although advances in single-cell technologies enables the characterization of multiple omics profiles in individual cell, extracting functional and mechanistic insight from such information remains a major challenge. Here, we present scapGNN, a graph neural network (GNN)-based framework that creatively transforms sparse single-cell profile data into the stable gene-cell association network for inferring single-cell pathway activity scores and identifying cell phenotype-associated gene modules from single-cell multi-omics data. Systematic benchmarking demonstrated that scapGNN is more accurate, robust, and scalable than state-of-the-art methods in various downstream single-cell analyses such as cell denoising, batch effect removal, cell clustering, cell trajectory inference, and pathway or gene module identification. scapGNN has been developed as a systematic R package that can be flexibly extended and enhanced for existing analysis processes and provides a new analytical platform for the study of single cells at the pathway and network level."

*2. Introduction: Many recent methods such as Refs.1-5 published in the last 2 years on the topic of deep genomic data analysis, gene co-expression network analysis and multi-omic data integration were not discussed. Please discuss the techniques (specially those developed on deep learning) and what are their limitations when compared to scapGNN.*

**REPLY:** Thank you for your suggestion. We have added the appropriate content and modified the introduction as shown below:

(1) Page 2, Introduction in the Manuscript.

"Moreover, these methods can only be based on pre-defined pathways or gene sets. Gene modules, which are structural sub-networks, have the same organizational patterns or functions and serve as building blocks of complex biological networks [19, 20]. Module-based analyses can achieve a higher-level understanding of the design and organization of biological systems. Genomap is an entropy-based cartography method to contrive the high-dimensional single-cell gene expression data into a configured image format and discover cell-specific gene sets [21]. Genomap simply calculates the average gene activity values for each gene in the specified cell type as the strength of association with the cell type. And genomap cannot provide significance-level p-values to screen for those genes that make up a meaningful gene set. How to autonomously and efficiently identify gene modules based on cell phenotype information is conducive to understanding the mechanism of cell state transitions and regulation of different cell phenotypes [22, 23].

With the development of deep learning techniques, many methods have been developed to extract low-dimensional features from high-dimensional single-cell data and integrated single-cell multi-omics data in a low-dimensional space. Cobolt constructed a multimodal variational autoencoder based on a hierarchical Bayesian generative model that projects the single-cell multi-omics data into shared latent space to perform visualization and clustering [24]. scDART is a deep learning framework that compresses scRNA-seq and scATAC-seq data into a shared space and aligns cells according to trajectories [25]. GLUE also enables single-cell multi-omics data integration by encoding cells into the latent space [26]. In contrast to the previous two methods, GLUE introduces the knowledge-based guidance graph via a graph autoencoder and extracts gene features to correct the alignment of cells in latent space. However, these methods share a common limitation: they all align cells from different omics data in latent space, which facilitates cell clustering and annotation, but has biological interpretations that make it difficult to extract deep mechanisms from the data. In addition, Cobolt and scDART rely on shared information, which leads to data loss, and only extracts low-dimensional features of cell and does not process genes, ignoring potential relationships between genes. scDART aligns cells in low-dimensional space according to cell trajectories, which may not be applicable to single-cell data without differentiation trajectories. GLUE introduces predefined knowledge-based guidance graphs, such as protein interaction networks, which may introduce noise beyond single-cell data. Some statistical frameworks, such as MOFA2 and UINMF, are also designed to integrate single-cell multi-omics data [27, 28]. MOFA2 builds on the Bayesian Group Factor Analysis framework to infer a low-dimensional representation of the data in terms of a small number of (latent) factors that capture the global sources of variability. UINMF derive a nonnegative matrix factorization algorithm for integrating single-cell datasets containing both shared and unshared features. These methods are still compressing data into low-dimensional features for data integration, which still fails to explain the biological mechanisms in single-cell multi-omics data. Integrating multi-omics data at the pathway and gene module level enables a comprehensive study of complex biological processes, highlights the interrelationship of relevant biomolecules and their functions, and can mine potential biological mechanisms that cannot be discovered by single omics data [29, 30]. Nevertheless, inferring active pathways and cell phenotype-associated gene modules supported by single-cell multi-omics data remains a gap.

Hence, we propose a uniform framework called scapGNN, a graph neural network (GNN)-based framework that infers and reconstructs gene-cell, gene-gene, and cell-cell association relationships for transforming sparse single-cell profile data into the stable gene-cell association network. Furthermore, the scapGNN integrates single-cell multi-omics data, calculates single-cell pathway activity scores, and identifies cell phenotype-associated gene modules by quantifying network information. The real and simulated single-cell datasets were used to benchmark the performance of scapGNN and demonstrate that it outperforms the state-of-art methods in multiple single-cell data analysis tasks."

*3. Benchmarking: I see that the benchmarking is done mostly with old techniques. Please compare your technique with at least 2 techniques that were published in last year or this year.*

**REPLY:** Thank you for your suggestion. We have added five new methods for benchmarking the performance of scapGNN, including UINMF, MOFA2, Seurat v4, scDART, and genomap. The revisions are as follows:

(1) Page 19, section "Inferring pathway activity scores by integrating scRNA-seq and scATAC-seq" in the Results.

"Next, we tested the performance of scapGNN to integrate single-cell multi-omics data. To evaluate the ability of the single-cell multi-omics supported pathway activity scores inferred by scapGNN to represent the cellular heterogeneity, we applied scapGNN to three single-cell multi-omics datasets with different sequencing platforms, different tissues, and species (S5 Table). UMAP plots of multi-omics supported pathway activity scores show that scapGNN can clearly distinguish between different cell types after integrating single-cell multi-omics information (S21A-C Fig). And compared to five state-of-the-art single-cell multi-omics integration methods (S2 Table), scapGNN shows excellent performance on several cell clustering indices (Fig 6A and B, S21D Fig).

In hair follicle tissue, transit-amplifying cells (TAC) can proliferate rapidly and produce three different mature cells, including the inner root sheath (IRS), hair shaft cuticle/cortex layer and medulla [48]. Recently, Jones et al. has applied SHARE-seq, an approach that enables the joint measurement of chromatin accessibility and gene expression from the same single cells to adult mouse skin tissue and resolved the differentiation process of TAC [48]. We applied scapGNN to integrate scRNA-seq and scATAC-seq data from the mouse skin dataset and to infer the pseudotime of TAC differentiation using Monocle 3 [49]. Compared to scDART (S2 Table), it is a method for integrating scRNA-seq and scATAC-seq data and can be used for trajectory inference on integrated data, scapGNN can show the differentiation trajectory of TAC more accurately (Fig 6C and S22 Fig). The pseudotime based on single-cell multi-omics supported pathway activity scores calculated by scapGNN has a higher similarity to the TAC differentiation pseudotime provided by Jones’s study [48] compared to scDART (Fig 6D).

We found that some significantly differential pathways were only found in the results by integrating multi-omics data, and those pathways have been documented to be involved in key biological functions of astrocytes and oligodendrocytes in the mouse brain cortex dataset (S6 Table). For T and B cells in the PBMC multi-omics dataset, the proportion of cells with T cell receptor signaling pathway or B cell receptor signaling pathway in the top 5 of the single-cell multi-omics supported pathway activity score ranked list was more than 0.95 (S23A Fig). For single-cell multi-omics data integration, scapGNN is also able to maintain the stability of pathway scoring (S24 Fig). Using genes in the T cell receptor signaling pathway or B cell receptor signaling pathway as the gold standard, the gene modules identified by scapGNN for T cells or B cells were able to accurately identify these genes with AUCs above 0.8 (S23B Fig). There was a consistent trend in the results for marker gene sets, with scapGNN being able to accurately identify the marker gene set corresponding to the cell type in both heterogeneous and homogeneous data (S23C Fig). ROC curves show that scapGNN can accurately identify highly active genes in the gene module using the marker gene set as the gold standard (S23D Fig). A number of studies have shown that the hedgehog signaling pathway is required for TAC developmental processes [48, 50, 51]. In particular, the transcriptome and epigenome activity of Gli2 and Gli3 in the hedgehog signaling pathway plays a critical role [50, 51]. This is confirmed by the violin plots of Gli2 and Gli3 activity in RNA and DNA in the mouse brain cortex dataset (S25A and B Fig). Consistent with this, scapGNN also detected high single-cell multi-omics supported pathway activity scores of the hedgehog signalling pathway during TAC development (S25C Fig). Gli2 and Gli3 also tend to co-expression during TAC development in the gene modules identified based on the combined gene-cell association network (S25D Fig).

These results demonstrate that scapGNN can effectively integrate single-cell multi-omics information at the pathway level to cluster cells, infer cell differentiation processes and accurately identify highly active pathways and cell phenotype-associated gene modules."

(2) Page 50, Fig 6 in the Manuscript.

(4) Page 54, S21 Fig in the Supporting information.

(5) Page 55, S22 Fig in the Supporting information.

(6) Page 55, S23 Fig in the Supporting information.

(7) Page 16, section "scapGNN accurately identified active pathways and cell phenotype-associated gene modules" in the Results.

"For homogeneous and heterogeneous data, we calculated the association scores of cell types with genes using cells belonging to the same type as a seed. These association scores were sorted in descending order. Using the marker genes of the corresponding cell type or the genes of the T and B cell receptor signaling pathway as the gold standard, the receiver-operating characteristic (ROC) curve analysis was used to assess the accuracy of scapGNN in identifying phenotype-associated gene module. In addition, we used genomap, an entropy-based cartography method for discovering cell- and class-specific gene sets from scRNA-seq data, as a benchmark method (S2 Table) [21]. The genomap gets activation values for each gene in the specified cell type. However, the training of the genomap model relies on the known truth cell labels of the dataset. When the labels of the cell types are all the same (i.e., homogeneous data), the genomap gets zero activity values for all genes. Therefore, we can only compare the performance of scapGNN and genomap on heterogeneous data. The results, as shown in Fig 3C-E, indicate that scapGNN can identify cell type-associated active genes either by marker pathway or by marker gene as a gold standard. And the accuracy and robustness of scapGNN are better than genomap (Fig 3C-E and S15 Fig). An additional advantage of scapGNN over genomap is the ability to provide significance-level p-values for the association scores of each gene with cell phenotypes."

(8) Page 49, Fig 3 in the Manuscript.

(9) Page 53, S15 Fig in the Supporting information.

*4. Discussion: The discussion section needs to be more rich. Specially, I would expect the authors to add some discussion on how scapGNN will enrich our understanding of single cell biology in case of new diseases such as COVID.*

**REPLY:** Thank you for your suggestion. We applied the scapGNN to the coronavirus disease 2019 (COVID-19) dataset, which includes healthy control and COVID-19 patient. We screened B-cell-associated differential pathways between healthy controls and COVID-19 patients. Consistent with previous studies, protein complex assembly, and protein transport related pathways are activated in the B cells of COVID-19 patients, as B cells require high levels of protein synthesis to perform their functions (DOI: 10.1016/j.immuni.2020.07.009) (S30 Fig). We also found that thyroid hormone synthesis pathway activity was significantly elevated in COVID-19 patient relative to healthy control (S30 Fig). Many reports have shown that thyroid hormone has a protective effect on COVID-19 patient and that low serum levels may increase mortality (DOI: 10.3389/fendo.2021.779692 and DOI: 10.1007/s40618-021-01506-7). We constructed gene module networks for B cells of three cell phenotypes (healthy control, COVID-19, and IAV) (S29 Fig). We have identified potentially highly active gene signatures of COVID-19 and therapeutic targets such as *NPM1* and *ARPC1B*. Due to the overwhelming amount of content, we have included the main content as a result and summarized them in the discussion. The relevant revisions are as follows:

(1) Page 24, section "Investigation of the biological mechanisms in COVID-19 based on scapGNN.

"Next, the scapGNN was applied to the coronavirus disease 2019 (COVID-19) dataset, which includes healthy control and COVID-19 patient (S29A Fig, S1 Table). We screened B-cell-associated differential pathways between healthy control and COVID-19 patient by taking the top 10 fold-change for each B-cell subtype in descending order, with the maximum p-value < 5 ×10^(-5) (S30 Fig). Coronavirus as an exogenous virus activates the host immune system. As expected, many of the differential pathways are associated with infections and immune diseases such as human cytomegalovirus infection and measles, suggesting that B cells have been activated to produce an immune response to exogenous viruses. Consistent with previous studies, protein complex assembly and protein transport-related pathways are activated in the B cells of COVID-19 patient, as B cells require high levels of protein synthesis to perform their functions [70]. The activity of the thyroid hormone synthesis pathway is significantly increased in COVID-19 patient (S30 Fig). Several reports have shown that thyroid hormone has a protective effect in COVID-19 patient and that low serum levels may increase mortality [71, 72]. Notably, the gene association network of the thyroid hormone synthesis pathway shows that ATP1B3 plays a key role in the pathway activity in COVID-19 patient (S29B Fig). Liu's study also found ATP1B3 to be one of the targets of COVID-19 infection [73].

We further integrated the scRNA-seq data of influenza A virus (IAV)-infected patient and constructed gene modules networks for B cells of three cell phenotypes (healthy control, COVID-19, and IAV) using false discovery rate (FDR) < 0.05 (S29C Fig). Genes co-expressed in the three cellular phenotypes may be disease independent. COVID-19-specific genes may be potentially high-activity gene signatures of COVID-19. For example, NPM1 is present in the COVID-19-associated PPI network module and is associated with enhanced viral replication of COVID-19 [74, 75]. COVID-19 and IAV co-expressed genes may be associated with essential biological functions of B cells, for example IGHG3 and IGLC2 are B cell marker genes [76]. Interestingly, the absence of ARPC1B, a health-specific gene that is reduced in COVID-19, leads to immunodeficiency [77, 78]. This may provide new insights into the pathogenic mechanisms and treatment of COVID-19."

(2) Page 28, Discussion in the Manuscript.

"The scapGNN also was applied to the COVID-19 dataset, which includes healthy control and COVID-19 patient. We are elucidating biological pathways closely associated with the onset and development of COVID-19 and identifying specific features of COVID-19 compared to IAV as well as potential therapeutic targets (S29 Fig and S30 Fig)."

(3) Page 56, S29 Fig in the Supporting information.

(4) Page 57, S30 Fig in the Supporting information.

References:
1. Islam, M.T., Xing, L. Cartography of Genomic Interactions Enables Deep Analysis of Single-Cell Expression Data. Nat Commun 14, 679 (2023).
2. Gao, C., Liu, J., Kriebel, A.R. et al. Iterative single-cell multi-omic integration using online learning. Nat Biotechnol 39, 1000-1007 (2021).
3. Islam, M.T., Wang, JY., Ren, H. et al. Leveraging data-driven self-consistency for high-fidelity gene expression recovery. Nat Commun 13, 7142 (2022).
4. Korsunsky, I., Millard, N., Fan, J. et al. Fast, sensitive and accurate integration of single-cell data with Harmony. Nat Methods 16, 1289-1296 (2019).
5. https://www.biorxiv.org/content/10.1101/2023.03.10.532124v1.abstract
