# Supplementary material for: scapGNN: A graph neural network–based framework for active pathway and gene module inference from single-cell multi-omics data
Source: PLoS Biol. 2023 Nov 13;21(11):e3002369. doi: 10.1371/journal.pbio.3002369 (PMC10681325; doi:10.1371/journal.pbio.3002369)
Supplement: Attachment — Submitted filename: Response.docx [file pbio.3002369.s053.docx]

Dear reviewers:

Thank for the reviewers’ comments and professional concerning our manuscript entitled “scapGNN: a graph neural network-based framework for active pathway and gene module inference from single-cell multi-omics data” (Manuscript ID: PBIOLOGY-D-23-00416R3). These opinions help to improve academic rigor of our paper, as well as the important guiding significance to our research. We have studied the comments carefully and have made corrections. Revised portions are marked in RED in the paper. Meanwhile, the manuscript has been reviewed and edited by language services of the International Science Editing (http://www.internationalscienceediting.com). The editing certificate (the file is Editing certificate.pdf) has also been uploaded into the system along with the revised manuscript. The main corrections in the paper and the response to the reviewer's comments are as follows:

*REVIEWS:*

*Reviewer #1: The author has given a very detailed replay to my comments but there are still two problems that need to be solved.*

*1. For Comment 5, two more multi-omics datasets used for evaluation should be added. There re only 3 multi-omics datasets and scapGNN has no advantage on one of the datasets. And, the scapGNN's cell clustering performance comparison between single omics and multi-omics cannot be done only through UMAP visualization, but also on cell clustering accuracy indicators.*

**REPLY:** We appreciate your constructive feedback, which will significantly improve the comprehensiveness and rigor of our study. It is important to use diverse datasets to evaluate the performance of scapGNN in single-cell multi-omics data integration. However, single-cell sequencing technologies that can detect multiple omics in the same cell are still an emerging field. Less data is currently available, which is also agreed by the editor in the editor’s *Specific points (ii)*. We are trying to contact relevant researchers to obtain single-cell multi-omics data that is not in a publicly available or usable format. Where possible, we will post the new results to a public repository such as GitHub.

Currently, three single-cell multi-omics datasets are used in our manuscript, include different species (human and mouse), different tissues (brain, skin, and peripheral blood), and different sequencing platforms (SNARE-seq, SHARE-seq, and 10x Multiome). These three datasets can represent different scenarios of single-cell multi-omics data analysis. Overall, scapGNN outperforms other state-of-the-art methods in cell clustering for single-cell multi-omics integration. For the mouse brain cortex dataset, scapGNN is the best in all three cell clustering indicators. For the other two datasets, no method can outperform scapGNN on all cell clustering indicators. For example, in the mouse skin dataset, Seurat's NMI score is higher than scapGNN, but ARI and Purity are lower than scapGNN. In addition to cell clustering, the greater strength of scapGNN is the integration of the gene level into the pathway level which makes the data analysis meaningful and interpretable. Based on combining gene-cell association networks from single-cell multi-omics, scapGNN also mines gene-gene association relationships and identifies cell phenotype-associated gene modules. Other methods only extract embedded features from single-cell multi-omics data to align the data in latent space. These methods are mainly designed to obtain cell clusters, but it is difficult to understand the biological mechanisms in depth.

Based on your suggestion, we have calculated the cell clustering indicators (ARI, NMI, and SW) for single omics and added them to the S21E Fig. The relevant revisions are as follows:

(1) Page 29, section Discussion.

"Single-cell sequencing technologies capable of detecting multiple omics in the same cell are still an emerging field with development of platforms such as SNARE-seq [77] and CITE-seq [79]. These technologies provided the basis for the application of scapGNN (Fig 6A and B, S21D and E Fig). It should be noted that based on the three cell clustering accuracy indicators (ARI, NMI, and SW) the quality of scATAC-seq data is inferior compared with that of the corresponding scRNA-seq data from multi-omics of the same cell, posing a challenge for methods that integrate single-cell multi-omics data (S21E Fig)."

*2. For Comment 12, why not use a PBMC dataset containing three and more cell types?*

**REPLY:** We gratefully appreciate for your comment. It is possible to evaluate the stability of scapGNN in identifying the pathway using a combination of more cell type compositions in the PBMC dataset. As in S14 Fig, we extracted more cell types from the original scRNA-seq data of the PBMC dataset to form a new dataset and found that scapGNN consistently and accurately identified either the T cell receptor signaling pathway or the B cell receptor signaling pathway. It should be noted that as more cell types are added, the structure of the newly constructed dataset becomes more similar to the original dataset, which makes the test less meaningful. The relevant revisions are as follows:

(1) Page 55, S14 Fig in the Supporting information.

*Reviewer #2: I will focus on the authors' response to my comments.*

*1. The authors added in the manuscript: "**Genomap simply calculates the average gene activity values for each gene in the specified cell type as the strength of association with the cell type. And genomap cannot provide significance-level p-values to screen for those genes that make up a meaningful gene set." The first sentence is scientifically incorrect. The second sentence is grammatically not good. For the first sentence, I would recommend the authors to read the referred paper carefully. For second sentence, I would recommend them taking help from grammar-correcting tools.*

**REPLY:** We feel sorry for our carelessness. We appreciate your thorough review and your attention to detail. We have reviewed the referenced paper more carefully to ensure that our characterization of genomap's calculation method is accurate and scientifically sound, and have amended the content accordingly. We have now worked on both the language and the readability, taking help from native English speakers for language corrections to ensure that the wording of our manuscript is clear, concise, and grammatically correct. The International Science Editing (http://www.internationalscienceediting.com) conducted edits on this manuscript. The editing certificate (the file is Editing certificate.pdf) has also been uploaded into the system along with the revised manuscript. The relevant revisions are as follows:

(1) Page 4, section Introduction.

"Genomap is an entropy-based cartography method to contrive the high-dimensional single-cell gene expression data into a configured image format and discover cell-specific gene sets [21]. It can compute cell type–specific gene importance scores by constructing the class-activation map. However, it does not evaluate the significance level of the cell type–specific gene importance."

*2. The authors discussed a few deep learning based techniques I referred in the response. However, I don't find them in the main manuscript.*

**REPLY:** We appreciate your comments. We did add a discussion of deep learning methods related to single-cell data analysis. We also compared the performance of these methods with scapGNN. However, indeed, we did not state this clearly in the manuscript but simply outlined it using the state-of-the-art method. We have revised the corresponding content to ensure the accuracy and consistency of our manuscript. We apologize for any confusion caused by this oversight and appreciate your understanding.

(1) Page 20, section "Inferring pathway activity scores by integrating scRNA-seq and scATAC-seq" in the Results.

"Compared with five state-of-the-art single-cell multi-omics integration methods (UINMF, MOFA2, Seurat, Cobolt, and GLUE), scapGNN showed better performance on several cell clustering indices (Fig 6A and B, S21D Fig, and S2 Table)."

*3. In Fig. S15, the authors compared the performance of genomap with scapGNN. However, the results look impractical. I would recommend the authors follow the example codes of genomap properly (specially the data standardization) to recreate the results. Please follow example 7 in https://github.com/xinglab-ai/genomap.*

**REPLY:** Thank you for pointing this out. Our implementation of genomap is based on the reference program file genoMapDemo.ipynb in version 1.0.4. The file can be downloaded from https://codeload.github.com/xinglab-ai/genomap/zip/refs/tags/v1.0.4. However, genomap has currently been updated to version 1.3.6. Example 7 in genomap version 1.3.6 is quite different from version 1.0.4. As you said in example 7 program of genomap version 1.3.6, there is no use of scipy.stats.zscore function to normalize the data. Therefore, we used the LogNormalize method to normalize the data and updated the corresponding results based on the example 7 program of genomap version 1.3.6. As shown in Fig 3C-E and S15 Fig, compared with version 1.0.4 in the previous version of the manuscript, genomap version 1.3.6 exhibits better identification of cell phenotype-related active genes and robustness especially for cell marker genes. The relevant revisions are as follows:

(1) Page 17, section "scapGNN accurately identified active pathways and cell phenotype-associated gene modules" in the Results.

"The results, as shown in Fig 3C-E, indicated that scapGNN could identify cell type–associated active genes using either marker pathway or marker gene as a gold standard. The genomap is also effective in identifying cell type-associated active genes, particularly for cell marker genes. Both scapGNN and genomap had good robustness for dropout noise (S15 Fig)."

(2) Page 51, Fig 3 in the Manuscript.

(3) Page 55, S15 Fig in the Supporting information.

**COMMENTS FROM THE ACADEMIC EDITOR**

*I agree with Reviewer #2 about the overall poor writing notably in the parts rewritten that resulted from the revision and the need to better evaluate genomap performance.*

**REPLY:** We sincerely appreciate the valuable comments. We have now worked on both the language and the readability, using the help of native English speakers for language corrections to ensure that the wording of our manuscript is clear, concise, and grammatically correct. The International Science Editing (http://www.internationalscienceediting.com) conducted edits on this manuscript. The editing certificate (the file is Editing certificate.pdf) has also been uploaded into the system along with the revised manuscript. Our implementation of genomap was based on the reference program file genoMapDemo.ipynb in version 1.0.4 in the previous version of the manuscript. The file can be downloaded from https://codeload.github.com/xinglab-ai/genomap/zip/refs/tags/v1.0.4. However, genomap has currently been updated to version 1.3.6. We have updated the corresponding results based on the genomap version 1.3.6. As shown in Fig 3C-E and S15 Fig, compared with version 1.0.4 in the previous version of the manuscript, genomap version 1.3.6 exhibits better identification of cell phenotype-related active genes and robustness especially for cell marker genes. The relevant revisions are as follows:

(1) Page 17, section "scapGNN accurately identified active pathways and cell phenotype-associated gene modules" in the Results.

"The results, as shown in Fig 3C-E, indicated that scapGNN could identify cell type–associated active genes using either marker pathway or marker gene as a gold standard. The genomap is also effective in identifying cell type-associated active genes, particularly for cell marker genes. Both scapGNN and genomap had good robustness for dropout noise (S15 Fig)."

(2) Page 51, Fig 3 in the Manuscript.

(3) Page 55, S15 Fig in the Supporting information.

*Even several of the revisions to Reviewer #1, although the additional analysis raised by the reviewer were performed, were not properly articulated in the new ms. (e.g. Point 1 on heterogenous and homogenous “marker” genes ; but also other points.)*

**REPLY:** Thank you for the comment. We have amended the relevant content and made the statement clearer. For example:

(1) Page 31, section "Data preprocessing" in the Materials and Methods.

"To assess the impact of highly variable gene selection on the performance of scapGNN, we performed benchmark experiments on different numbers of highly variable genes. For heterogeneous single-cell data, cell clustering accuracy and marker gene set scoring decrease as the number of genes increases (S32 Fig). Because low amounts of mRNA in individual cells, inefficient mRNA capture, as well as the stochasticity of mRNA expression can lead to dropout events in single-cell data [82], and not all genes contribute to cell-to-cell differences [83], too many genes can introduce technical noise. We selected 2000 highly variable genes in processing heterogeneous single-cell data. For homogeneous data, there is little variation in expression levels between cells for inherently expressed genes such as marker genes. A small number of highly variable genes can cause a loss of information (S33 Fig). Our results show that 8000 highly variable genes can represent information about intrinsic biological pathways or marker gene sets in cells while minimizing the introduction of noise (S33 Fig). We selected 8000 highly variable genes in processing homogeneous single-cell data."

(2) Page 37, section "Constructing the cell community network" in the Materials and Methods.

"An important feature of the network is the existence of the community or cluster structure which is defined as a group of nodes having similar affiliations but different to rest of the network. In this study, we identify cell communities in the cell-cell association network by the community detection algorithms such as edge-betweenness, leading eigen, and louvain. Cells from the same community may tend to have similar biological functions. We fused cells belonging to the same phenotype into one cell community node and merged edges between two cell communities by averaging weights to generate a cell community network. In this way, it will be feasible to show the strength of the association between different cell types or cell communities."

(3) Page 26, section Discussion.

"Single-cell data has a large number of drop-out events, leading to difficulties in data analysis at the gene level [76]. Most single-cell clustering methods only use genes as features of cells, ignoring the relationship between genes, which could make clustering methods more susceptible to noise, resulting in low accuracy and robustness. Pathway-level analysis is less affected by the noise on a single gene [1], and can provide better biological interpretation and insights [6]."

(4) Page 2, section Introduction.

"Biological pathways in distinct cell types have different activation patterns, which facilitates the understanding of cell functions. In single-cell studies, pathway activation analysis has become a powerful approach for the extraction of biologically relevant signatures to uncover the potential mechanisms of cell heterogeneity and dysfunction in human diseases [4, 5]."

*So the author should put effort in the writing (English and precise articulation)*

**REPLY:** Thank you for the comment. We have carefully revised the manuscript according to the comments and suggestions. According to advice, this manuscript was edited for proper English language, grammar, punctuation, spelling, and overall style by one or more of the highly qualified native English speaking editors at International Science Editing. The editing certificate (the file is Editing certificate.pdf) has also been uploaded into the system along with the revised manuscript.

*Specific points:*

*(i) I agree with Reviewer #1 that visual UMAP analysis is not solid way for performance comparison -this point can be addressed.*

**REPLY:** Thank you for the comment. Based on your suggestion, we have calculated the cell clustering indicators for single omics and added them to the S21E Fig. The relevant revisions are as follows:

(1) Page 29, section Discussion.

"Single-cell sequencing technologies capable of detecting multiple omics in the same cell are still an emerging field with development of platforms such as SNARE-seq [77] and CITE-seq [79]. These technologies provided the basis for the application of scapGNN (Fig 6A and B, S21D and E Fig). It should be noted that based on the three cell clustering accuracy indicators (ARI, NMI, and SW) the quality of scATAC-seq data is inferior compared with that of the corresponding scRNA-seq data from multi-omics of the same cell, posing a challenge for methods that integrate single-cell multi-omics data (S21E Fig)."

*(ii) What is questionable is the request for testing more muti-omics data sets. For single-cell analysis these are rare and of questionable quality – I am not aware of any good ones. Reviewer #2 had suggested to deemphasize the multi-omics aspect and that is more appropriate. The authors should adjust the text and discussion accordingly. Eg pointing to these challenges of getting good quality multi-omics datasets – which are only now appearing on he “market” and present the multiomics as additional possible filed of applying their algorithm.*

**REPLY:** We gratefully appreciate for your comment. As you mentioned, single-cell sequencing technologies that can detect multiple omics in the same cell are still an emerging field. The quality of multi-omics data obtained from the same cell is inferior, particularly for scATAC-seq data, posing a challenge for methods that integrate single-cell multi-omics data. The three single-cell multi-omics datasets used in our manuscript, include different species (human and mouse), different tissues (brain, skin, and peripheral blood), and different sequencing platforms (SNARE-seq, SHARE-seq, and 10x Multiome). These three datasets can represent different scenarios of single-cell multi-omics data analysis. Overall, scapGNN outperforms other state-of-the-art methods in cell clustering for single-cell multi-omics integration. The relevant revisions are as follows:

(1) Page 29, section Discussion.

"Single-cell sequencing technologies capable of detecting multiple omics in the same cell are still an emerging field with development of platforms such as SNARE-seq [77] and CITE-seq [79]. These technologies provided the basis for the application of scapGNN (Fig 6A and B, S21D and E Fig). It should be noted that based on the three cell clustering accuracy indicators (ARI, NMI, and SW) the quality of scATAC-seq data is inferior compared with that of the corresponding scRNA-seq data from multi-omics of the same cell, posing a challenge for methods that integrate single-cell multi-omics data (S21E Fig)."

*(iii) Similarly, PBMC sets with more cell types is readily available – but I wouldn’t make this essential since this is already a complex manuscript.*

**REPLY:** Thank you very much for your comments and for all the work you do on our manuscript. Our application of scapGNN to data with different combinations of cell types was to test the stability of scapGNN, and to show that its performance in detecting pathways is not affected by changes in cell type combinations. To better demonstrate the stability of scapGNN on data with combinations of different cell types, we extracted more cell types from the original scRNA-seq data of the PBMC dataset to form a new dataset, and found that scapGNN consistently and accurately identified either the T cell receptor signaling pathway or the B cell receptor signaling pathway (S14 Fig). It should be noted that as more cell types are added, the structure of the newly constructed dataset becomes more similar to the original dataset, which makes the test less meaningful. The relevant revisions are as follows:

(1) Page 55, S14 Fig in the Supporting information.
